# Changes in the recent habitat suitability of Euro-Mediterranean *Anopheles* species due to land-use and climate

**DOI:** 10.1186/s13071-025-07115-0

**Published:** 2025-11-27

**Authors:** Christian Merkenschlager, Freddy Bangelesa, Heiko Paeth, Elke Hertig

**Affiliations:** 1 Regional Climate Change and Health, Faculty of Medicine, University of Augsburg, Universitätsstr. 2, 86159 Augsburg, Germany; 2 Institute of Geography and Geology, Universtiy of Wuerzburg, Am Hubland, 97074 Würzburg, Germany; 3 Kinshasa School of Public Health, University of Kinshasa, P.O. Box 11850, Kinshasa, Democratic Republic of the Congo

**Keywords:** Anopheles, Habitat suitability, Climate change, Land-use change, Euro-Mediterranean area

## Abstract

**Background:**

Habitat suitability of *Anopheles* mosquitoes depends on appropriate climate and land-use conditions. *Anopheles* mosquitoes are the main vectors for malaria transmission in the Euro-Mediterranean region, and there are major concerns that these species will expand and/or shift their range due to the expected changes in climate and land-use. This study aims to identify the main climate and land-use drivers of changes in the habitat suitability for six different *Anopheles* species between 2000 and 2020 within the Euro-Mediterranean region.

**Methods:**

Boosted regression trees were applied to establish the link between climate and land-use predictors and habitat suitability. An ensemble of 16 models, based on different methods of selecting background points and statistical predictors, was applied to each species. The ensemble was evaluated by means of model skill and transferability to identify the best model. Taking contribution, interactions and response range into account, the most important predictors and those responsible for changes were identified.

**Results:**

The model ensembles agreed on the direction of change for four *Anopheles* species within the study area, with two of these showing an overall increase (*An. atroparvus, An. sacharovi*) of areas with suitable conditions and two showing a decrease (*An. messeae, An. sergentii*). Climate change was found to be the main driver of shifts in habitat suitability, with only a few models attributing changes mainly to land-use. The limited influence of land-use changes may be due to the spatial resolution being too coarse. For most species, temperature-related bioclimatic variables (BIO4, BIO5, BIO8) were the most important predictors of changes in habitat suitability. A superior method for either the specific background points or predictor selection did not emerge because the relative ranking of the corresponding models is dependent on the species analyzed.

**Conclusions:**

Between 2000 and 2020, rising temperatures were the main driver of changes in the habitat suitability of *Anopheles* mosquitoes in the Euro-Mediterranean region, with land-use changes having a relatively minor impact. In particular, regions to the north of the respective distribution area were found to be characterized by an increasing habitat suitability, while regions to the south showed decreasing trends. These trends may also impact the risk of local malaria transmission in these regions.

**Graphical abstract:**

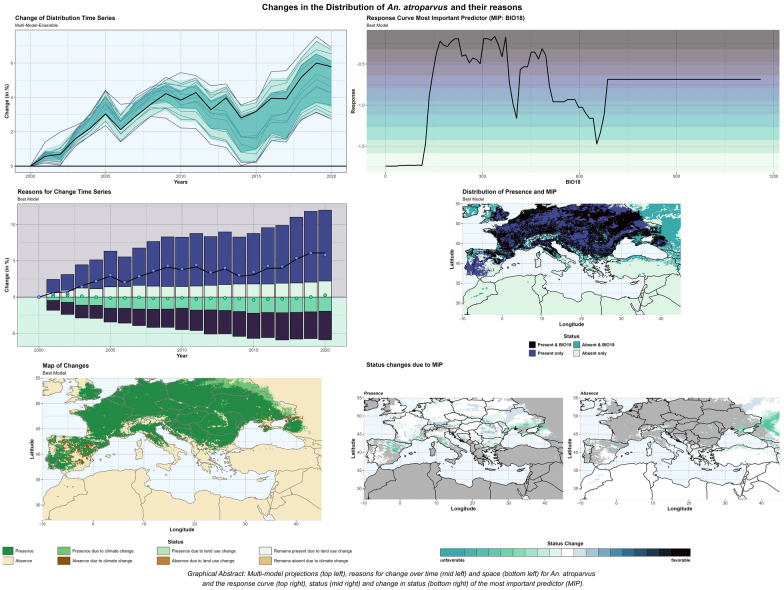

**Supplementary Information:**

The online version contains supplementary material available at 10.1186/s13071-025-07115-0.

## Background

*Anopheles* mosquitoes are the primary vectors of malaria, causing an estimated 249 million cases worldwide in 2022, of which 608,000 were fatal. It is noted that more than 50% of all deaths related to vector-borne diseases can be attributed to *Anopheles* species [1]. Mosquitoes and mosquito-borne diseases (MBDs) are commonly associated with tropical and subtropical regions [2], but some *Anopheles* species are also native to the Mediterranean area and Europe. Historical records indicate malaria was present in Europe as early as 500–400 BC [3], with autochthonous transmission until the end of World War II [4, 5]. Due to socio-economic improvements, such as wealth, life expectancy and urbanization [6], malaria was almost eradicated from Europe during the second half of the twentieth century [7], but in the recent past, autochthonous malaria transmissions were recorded in Germany, the Netherlands, Greece, Italy, France and Spain [8], indicating the potential for re-emergence under favorable conditions. Thus, despite centuries of control efforts, the control of MBDs remains a challenge of enormous public health importance [9, 10].

The potential for the introduction or re-emergence of MBDs in an area depends mainly upon receptivity, vector infectivity and vulnerability [11]. Receptivity takes into consideration the presence of a competent vector and suitable ecological and climatic conditions necessary for the vector’s survival and development. Vector infectivity is contingent upon the susceptibility of the vector to the *Plasmodium* species responsible for its infection. Vulnerability depends on the introduction and maintenance of a human reservoir [8]. In the present study we focus on receptivity. Globally, the geographic distribution and abundance of MBDs and mosquitoes are expected to increase due to changes in climate, land-use and/or socioeconomic conditions [12]. As mosquitoes are ectothermic, changing temperatures affect the transmission season, vector development and biting rate (e.g. [13, 14]), while changing precipitation patterns affect the aquatic stages of the mosquitoes [15]. On the other hand, changing land-use can reinforce or reduce the impacts of climate change. In particular, irrigation, deforestation and urbanization modify habitat suitability, but their impact varies by geographic region and local characteristics [12].

The invasion of mosquitoes into new areas and their reappearance in previously eradicated areas are due to several factors. While life-cycle dynamics, survival and reproduction rates are more dependent on climate [16], land-use [17] and host availability [18], mosquito dispersal is mainly dependent on high mobility, international trade and globalization [4]. The focus of this study is on the habitat suitability area (HSA) for which dispersal factors play only a minor role. The native *Anopheles* species studied are primarily anthropophilic, but also show zoophilic, mammalophilic or opportunistic feeding habits [19, 20]. Thus, the Mediterranean countries with a population density of 63.3 inhabitants per square kilometer [21] and a large population of grazing livestock, representing an important economic sector [22], are not limited in terms of host availability; thus, the HSA in these countries depends mainly on changes in climate and land-use.

Climate and environmental variables are key drivers of mosquito distribution [23], with climatic predictors believed to be the most dominant variables, as mosquitoes are highly dependent on specific temperature regimens [4] and water resources are inextricably linked to the immature stages of mosquitoes [17]. Bezirtzoglou et al. [24] state that weather affects the timing and intensity of outbreaks while climate affects the range of infectious diseases. However, depending on its immediacy and strength, land-use change may prove to be the most important driver of recent disease emergence and spread in some regions [12, 25].

Given the projections of climate change from climate models, it is very likely that higher temperatures will increase the length of the transmission season, expand or shift the geographic range of mosquitoes, accelerate the frequency of biting and egg-laying, and, consequently, increase the risk of MBDs [15, 26, 27]. In particular, temperate countries may be most threatened by the emergence and re-emergence of MBDs [28], while regions which currently have warmer climates may experience a decreasing risk as biting rates decrease once a certain upper temperature threshold has been surpassed [29]. In addition, the distribution and abundance of mosquitoes is also positively correlated with precipitation [30, 31], which impacts the reproduction and survival of mosquitoes [23]. However, when heavy rainfall occurs, the downpour can wash away breeding sites and destroy eggs, resulting in a reduced mosquito population [15]. As the Mediterranean region is considered to be a hotspot for climate change, with rising temperatures, increasing precipitation variability and, for some regions, reduced annual rainfall amounts [32], significant changes in the distribution of vectors and pathogens are expected [24].

With respect to land-use, human modification of the natural environment can promote the creation of new habitats where mosquitoes can thrive if no countermeasures are taken [17]. The influence of land-use changes on the distribution and population dynamics of mosquitoes depends considerably on several factors, including geographic region or type of change [12]. A central feature of agriculture within the Mediterranean area is irrigation. Irrigation practices can lead to the expansion of breeding habitats [33] and extension of the disease transmission season [34], even though the climatic conditions may remain unsuitable. Due to a wide range of habitats that vary in microclimate [35] and socioeconomic structure [36], the impact of urbanization on the risk of MBDs is complex, as both the retreat and spread of mosquitoes and MBDs can be observed [12]. The so-called urban heat island [37] leads to higher average temperatures compared to the rural environment and flattens the curve of the diurnal temperature range [36], affecting both the development of mosquitoes and the transmission of diseases [38]. In addition to temperature-related aspects, urban areas also favor anthropophilic mosquitoes due to a higher host availability [39] and the preference of to breed in artificial water-filled containers like bird baths, flowerpots or used tires [40]. *Anopheles* species mainly prefer natural breeding sites but they are also found in artificial containers [19]. Deforestation alters local environmental and climatic conditions that also affect habitat suitability for mosquitoes [41], particularly the reproduction, abundance and composition of mosquito species [17]. The direction of change depends on the respective mosquito species but, in general, deforestation favors vector mosquitoes [42]. One question that arises with deforestation is whether the increase in mosquitoes depends on deforestation directly or whether it is the result of subsequent land-use. Indeed, as deforestation is mostly due to the expansion of agricultural land, pastures or settlements, the availability of humans or livestock for blood-feeding mosquitoes increases with deforestation, thus improving habitat suitability [41].

In the present study we focused on the recent evolution (2000–2020) of the HSA of six different *Anopheles* species within the extended Mediterranean area: *Anopheles atroparvus* (van Thiel 1927), *Anopheles labranchiae* (Falleroni 1926), *Anopheles messeae* (Falleroni 1926), *Anopheles sacharovi* (Favre 1903), *Anopheles sergentii* and *Anopheles superpictus* (Grassi 1899). We used a set of 38 climatological and 14 land-use variables to run boosted regression tree (BRT) models to determine the HSA of the *Anopheles* species. Different methods of background point selection were tested, and we reduced the initial set of climatological predictors through different selection steps. In total, we constructed 16 different model setups for each species. By applying various tests of skill and applicability, we determined the best model for each species. Subsequently, the best models were projected to recent climatic conditions twice, once with varying land-use and once with constant land-use. Comparing the two runs allowed us to estimate the relative contribution of climate and land-use to the projected shifts in the HSA. Finally, we highlighted the most important predictors responsible for changes in HSA.

## Methods

### Study area

To include expansions of the HSA of the different mosquitoes within the Mediterranean area due to the expected climate change, for the present study we extended the study area northwards (10 °W–45 °E, 27 °N–55 °N). The total resolution was 0.1° × 0.1° (approx.  90 km^2^ per grid box). The region contains 154,000 grid boxes, of which 113,054 are over land areas. The first-order conservative remapping algorithm of the Climate Data Operator [43] was used to interpolate all datasets used in this study to the respective grid where necessary.

According to the Koeppen–Geiger classification, the Mediterranean is characterized by a warm climate with dry summers and wet winters (Cs-climate). The neighboring regions to the north represent regions characterized by a temperate oceanic climate (Cfb) in the west and by a warm and humid continental climate (Dfb) in the east of Europe. Overall, the Mediterranean region has a high population density of 63.3 inhabitants per square kilometer, with significantly higher densities in coastal areas. During the summer season, human density increases, especially along the coasts due to an influx of > 300 million tourists per year [44]. In addition to tourism, agriculture is an important economic factor in the Mediterranean region, with 237.6 million hectares across the Mediterranean countries classified as agricultural land, 10% of which is irrigated [21, 45]. Thus, irrigation is a central feature of Mediterranean agriculture, with massive investments made by all countries in the second half of the twentieth century in large dams, inter-basin transfers and public irrigation schemes [46]. Also, grazing livestock production remains an important economic factor in the Mediterranean agricultural sector [22].

#### Observational dataset

Observations of the dominant *Anopheles* vectors were downloaded from the Malaria Atlas Project (MAP) [47, 48] homepage. The maps generated from the MAP provide valuable insights into the anticipated geographical ranges of the dominant *Anopheles* species for the year 2010. In this context, “dominant” refers to vectors that have been identified as either main, dominant or important vectors [19]. These maps include probabilities of presences and are based on extensive research and data analysis, with the incorporation of factors such as climate, habitat suitability and historical species distribution patterns. The predicted distributions mentioned in the text are derived from observations made between 1985 and 2009, as reported by Sinka et al. [49]. These observations serve as the foundation for the projections made in this study regarding the HSAs.

#### Climatological dataset

Climatological data with an hourly temporal resolution and a spatial resolution of 0.1° × 0.1° were downloaded from the Copernicus Climate Data Store (CCDS). In the present study, we used the ERA5-Land reanalysis dataset from 1970 to 2020 [50, 51]. The first 19 bioclimatic variables (BIOs), based on the BIOCLIM program by Nix [52], were generated using the ERA5-Land dataset. Because mosquitoes are mobile species that can hide or move when local conditions are temporarily unsuitable, we calculated mean BIOs using 31-year periods (see the climatological approach in Merkenschlager et al. [53]) to represent long-term climatological conditions, since long-term climatological conditions are more important for the establishment of mosquitoes than annual aggregated values. The bioclimatic variables used in this study are listed in Additional file 1 Predictor variables: Table S1.1.

For invasive species, such as mosquito species belonging to genus *Aedes*, many studies have investigated temperature and precipitation thresholds for certain life-cycle stages (e.g. [54–56]). To the best of our knowledge, there are no similar studies involving *Anopheles* mosquitoes. However, since we believe that extreme variables (EXVs) based on certain thresholds are essential for survival, we used a set of extreme variables as predictors based on common thresholds. For example, we extracted the number of days (ND) and the longest period (LP) when the mean or maximum temperature was < 0 °C. For mosquito species tolerant to cold (e.g. *Anopheles messeae*) or warm (e.g. *Anopheles sergentii*), we also included ND and LP for minimum and maximum temperatures. For precipitation, we used predictors representing extremely wet or dry days or periods. An overview of the set of extreme variables is given in the Additional file 1 Predictor variables: Table S1.2 and further information is available in Merkenschlager et al. [57]. By considering these factors, we aimed to gain a more comprehensive understanding of the temperature preferences and adaptability of these mosquito species.

The set of climatological predictors was complemented by absolute humidity (AHUM) as moisture is an important factor determining breeding site availability and egg survival [58]. According to Dickens et al. [59], AHUM can be one of the most important predictors in arid regions.

 In total, we included 38 climatological predictors, represented by 19 BIOs, 18 EXVs and AHUM. To model the HSA of *Anopheles* species, we averaged the predictors over a 31-year period, with the last year of the period representing the year of interest. For example, to model the HSA of 2020, we used the predictor variables of the period 1990–2020.

#### Land-use dataset

Land-use and land cover data sets for the period 2000–2020 were obtained from the historical and future land-use and land cover change dataset, LUC version 1.1, provided by Hoffmann et al. [60, 61]. The datasets are based on the Land Use and Coverage Area frame Survey (LUCAS) of the European Soil Data Centre (ESDAC), which includes 16 land-use classes, 14 of which occur in the study area; tropical land-use classes do not occur in the extended Mediterranean area and two land-use classes were therefore not included in the analysis. Annual land-use fractions for the period 1950–2015 (historical) and 2016–2100 (future) were provided at a spatial resolution of 0.1° × 0.1°, covering the area between 56 °W and 84 °E and between 16 °N and 79 °N. The future projections are based on the Shared Socioeconomic Pathways scenario SSP585, and both datasets were provided by the World Data Center for Climate (WDCC) at the German Climate Computing Center (DKRZ). In contrast to climate-related predictors, we only considered the year of interest for land-use. For example, to model the HSA of 2020, we only used the land-use of 2020. The underlying rationale is that changes in land-use directly affect the HSA on a local scale. The land-use classes considered in the analysis are listed in Additional file 1 Predictor variables : Table S1.3.

### Boosted regression trees

We used BRT models due to their ability to handle complex interactions and non-linear relationships between predictors and species presence, and for their suitability for datasets incorporating pseudo-absence points [62]. BRTs combine regression trees and boosting, with the aim to minimize a loss function by optimizing the number of trees, learning rate and tree complexity [63]. In general, the higher the learning rate, the smaller the numbers of trees added to the model. To achieve an adequate number of trees (1000+), the learning rate must be small as it reduces the contribution of the trees to the model [62]. The tree complexity (number of nodes in a tree) should reflect the true interaction order of the modeled response, but this is usually unknown and should be set by independent data [64]. Tree complexity also affects the learning rate, and thus the optimal number of trees. The more complex the trees, the lower the learning rate has to be to minimize prediction error [63].

In the present study, we followed the approach of Elith et al. [62] using deviance reduction as the performance measure in order to determine learning rate, tree complexity and number of trees by means of a tenfold cross-validation approach. As the observational dataset of MAP was based on predicted probabilities—and not on observed presences and absences—we applied a threshold of 0.5 to discriminate between presences (≥ 0.5) and absences. We checked four different approaches for selecting pseudo-absences: (i) method I, random selection with no constraints (absences < 0.5); (ii) method II, sample selection, where we set the restriction that the composition of the main land-use classes across the pseudo-absence points was similar to that across the study area (absences < 0.5); (iii) method III, buffered selection using the MAP probabilities to draw a buffer zone around presence points (probabilities > 0 and < 0.5), with only pseudo-absences selected from outside the buffer range (absences = 0); and (iv) method IV, sampled + buffered combining the second and third selection method. The model was run with 10,000 randomly selected presences and pseudo-absences each, with half of the points used for calibration and the other half for validation (bag fraction = 0.5). If the number of presences was < 10,000, we used 75% of the presences and added additional pseudo-absences to achieve a total number of 20,000 grid boxes for model calibration. To achieve a sufficient number of trees, we started with a learning rate of 0.15 and a tree complexity of 5 and looped the model. In the case that the number of trees was insufficient, the learning rate and/or tree complexity were decreased accordingly; conversely, if the number of trees was excessive, the learning rate and/or tree complexity were increased. These adjustments were made until the number of trees fell within the range of 1200 to 2000. The loop was designed to never have a tree complexity < 3 or > 10.

In addition, different within-model predictor selection methods were applied on the data for model setup. Since expert knowledge on the different *Anopheles* species is limited, we ran the model with all available predictors using the ‘gbm.step’ function (version 2.9) from Elith et al. [62]; Additional file 1: Method I). As second step, we subsequently removed irrelevant predictors using the ‘gbm.simplify’ function (method II) that is also part of the supplement of Elith et al. [62]. Methods III and IV are limited to climatological predictors only and were used to further eliminate unserviceable predictors. We ran the model only with predictors that achieved a contribution > 1 and/or predictors that lay above the double median of the interactions (method III). According to Elith et al. [62], the residual variance indicates the relative strength of the interaction between two predictors. Thus, the respective interaction size of the model output was assigned to both predictors. Since each predictor can interact with more than one other predictor, we summed up all the interactions for each predictor. If the summarized interaction size was greater than the doubled median, the predictor was considered for further analysis. We chose the doubled median of the model output as the threshold since all interaction terms were ultimately considered twice. Finally, in method IV, we also removed correlated predictor variables by applying the Pearson correlation coefficient with a threshold of $\left|r\right|>0.7$. The correlated predictor with the highest contribution was kept (method IV). Since the set of predictors is rearranged for each tree, variables can be considered separately, and correlations between predictors should not matter [65]. However, due to the principle of parsimony (Ockham’s razor), we tried to eliminate predictors that carry similar information without sacrificing model skill. The model setup was performed in R (version 4.5.1) using RStudio (version 2025.05.1 + 513) and the “gbm” package (version 2.2.2) (R Core Team, R Foundation for Statistical Computing, Vienna).

For each model (*m*), overall model skill (OMS) was calculated by means of the True Skill Statistics (TSS) metric, the spatial Brier Skill Score (BSS), the area under the curve (AUC) and Cohen’s kappa (CK) with the spatial sorting bias (SBB) removed for the latter two metrics. The AUC and CK were calculated by the ‘evaluate’ function of the ‘dismo’ R-package (version 1.3–16). TSS and CK were both computed using as threshold value (to binarize model predictions), which maximized the TSS. However, the authors of some studies (e.g. [66, 67]) argue that CK and AUC inherently depend on prevalence, thereby introducing statistical artefacts to assessments of predictive accuracy. Hijmans [68] emphasizes that, in general, the larger the extent used to select background points, the higher the AUC. Since the area for selecting background points in our study was very large, we followed an approach recommended by the author to remove the spatial sorting bias through point-wise distance sampling. Except for ${\text{AUC}}_{\text{SSB}}$, all skill metrics fall within the range of 0 to 1, where 1 represents a model perfectly discriminating between presences and absences, and 0 represents a model that is no better than random chance. For ${\text{AUC}}_{\text{SSB}}$, a perfect model is also represented by 1, while a model that performs no better than chance is represented by a value of ≤ 0.5. Applying feature scaling with a minimum of 0.5 and a maximum of 1 to the ${\text{AUC}}_{\text{SSB}}$ makes the scale similar to those of the other metrics, with 1 representing a perfect model and 0 representing a model that is no better than random. This allows the OMS to be averaged over all metrics.

1 $${\text{OMS}}_{m}=\frac{{\text{TSS}}_{m}+{\text{AUC}}_{{\text{SSB}}_{m}}+{\text{CK}}_{{\text{SSB}}_{m}}+{\text{BSS}}_{m}}{4}$$

In addition, transferability was checked by using the area of applicability (AoA) of Meyer and Pebesma [69]. The AoA also ranges from 0 to 1, with 1 representing a model that is fully applicable to the entire study area. To identify the best model, we calculated a score based on distance of the OMS and AoA from the origin of a coordinate system (0, 0) divided by $\sqrt{2}$, which represents a perfect model. By dividing by $\sqrt{2}$, the score is also bounded by 0 and 1. Including the OMS and AoA in the score calculation rewards high values for both metrics. This ensures that the model adequately captures the relationships between environmental variables and the likelihood of species occurrence, while reducing the likelihood of overfitting.

2 $${\text{SCORE}}_{m}=\frac{\sqrt{{\text{OMS}}_{m}^{2}+{\text{AoA}}_{m}^{2}}}{\sqrt{2}}$$

We used the unweighted multi-model ensemble (MME) as well as the model with the highest score to present the results for the HSA of the *Anopheles* species. For each model setup, the considered predictors, their contribution and interactions are provided in the Additional file 2 Model evaluation: Tables S2.1.1, S2.1.2, S2.1.3.

#### Evaluation of the reasons for changes in the HSA

In the present study, we analyzed changes in terms of the causes of change, i.e. if the changes in the HSA were due to climate or land-use change. Therefore, we ran the model twice, once with constant land-use (CLU) and once with varying land-use (VLU). The first year of the time series (2000) represented the constant land-use of the CLU approach and the reference (REF) for both CLU and VLU. The results of both approaches were then compared to the results of the REF. An overview of the change reason categories is given in Table 1. We acknowledge that in some cases only the combined effect of land-use and climate leads to changes in the HSA. In these cases, we attributed the changes to land-use because land-use changes interact on a local scale and changes in land-use usually have a direct effect on mosquito distribution. In contrast, climate acts on a larger scale and its effects on mosquito dynamics are usually more delayed. In the following text, all results refer to the assessed HSA of the reference year 2000 (not the study area or the land area); i.e. the HSA of the reference always represents 100%.

**Table 1:** Categories of changes in the habitat suitability area (0 = absent, 1 = present)

*N*	REF	CLU	VLU	Category	Acronym^a^
1	1	1	1	Present	P
2	0	0	0	Absent	A
3	0	1	1	Change to present due to climate change	PCC
4	1	0	0	Change to absent due to climate change	ACC
5	0	0	1	Change to present due to land-use change	PLUC
6	1	1	0	Change to absent due to land-use change	ALUC
7	1	0	1	Remaining present due to land-use change	RPLUC
8	0	1	0	Remaining absent due to land-use change	RALUC

*CLU* Constant land-use,* REF* reference,* VLU* varying land-use

^a^P, Present; A, Absent; PCC, Change to present due to climate change; ACC, Change to absent due to climate change; PLUC, change to present due to land-use change, ALUC, change to absent due to land-use change; RPLUC, remains present due to land-use change; RALUC, remains absent due to land-use change

#### Identifying the most important predictor

To identify the most important predictor (MIP) for presence or absence per grid box (not necessarily the predictor responsible for changes), we considered the contribution, potential interactions and the response range. The model output statistics provided contribution and interaction information for each predictor, and the response range was extracted from each predictor's response curve. Specifically, we analyzed the absolute change in each predictor variable between the reference and the year of interest for each grid box. Subsequently, we transferred both absolute values (i.e. reference and year of interest) on the response curves and checked whether the model responded to these absolute changes. The difference in response between reference and year of interest represents the response range. The response ranges were incorporated into the analysis, since substantial absolute changes in the predictor could have small responses on the status of the respective species, and vice versa. All factors were then normalized by feature scaling (all terms vary between 0 and 1) to avoid a weighting in favor of different scales. For the normalization of the responses, we used absolute values. A weighting by means of the contribution was then included for the interactions of the predictors because some predictors contributed much (little) to the model but had low (high) interactions.

3 $${\text{MIP}}_{\text{GB}}=\underset{P}{\text{max}}\left\{\left({\text{CON}}_{{N}_{P}}+ {\text{CON}}_{{N}_{P}}\cdot {\text{INT}}_{{N}_{P}}\right)\cdot{R}_{{N}_{{P}_{GB}}}\right\}$$

With:

$\text{MIP}$is the most important predictor

${\text{CON}}_{N}$is the contribution (normalized).

${\text{INT}}_{N}$is the interaction (normalized).

${R}_{N}$is the response range (normalized).

$P$is the predictor.

$\text{GB}$is the grid box.

Using unweighted interactions to determine the most important predictors resulted in more (less) important predictors being less (more) pronounced. The product of the normalized factors represents the index for identifying the MIP. This ensured that if there were no observed changes or the predictor was not important, the product of the factors would be zero. Finally, for each grid box, the ${\text{MIP}}_{\text{GB}}$ was divided by the sum of all significant predictors and multiplied by 100 to give the importance in percentage. The MIP was analyzed separately for climate-related and land-use-related variables. To identify the most important predictor for change (MIPC), we used the same approach as described above but under consideration of the sign of change in the response.

All figures were created using the R package ‘ggplot2’ (version 3.5.2) under R and RStudio (R Core Team, R Foundation for Statistical Computing, Vienna).

## Results

### Model setup and evaluation

**Fig. 1:**
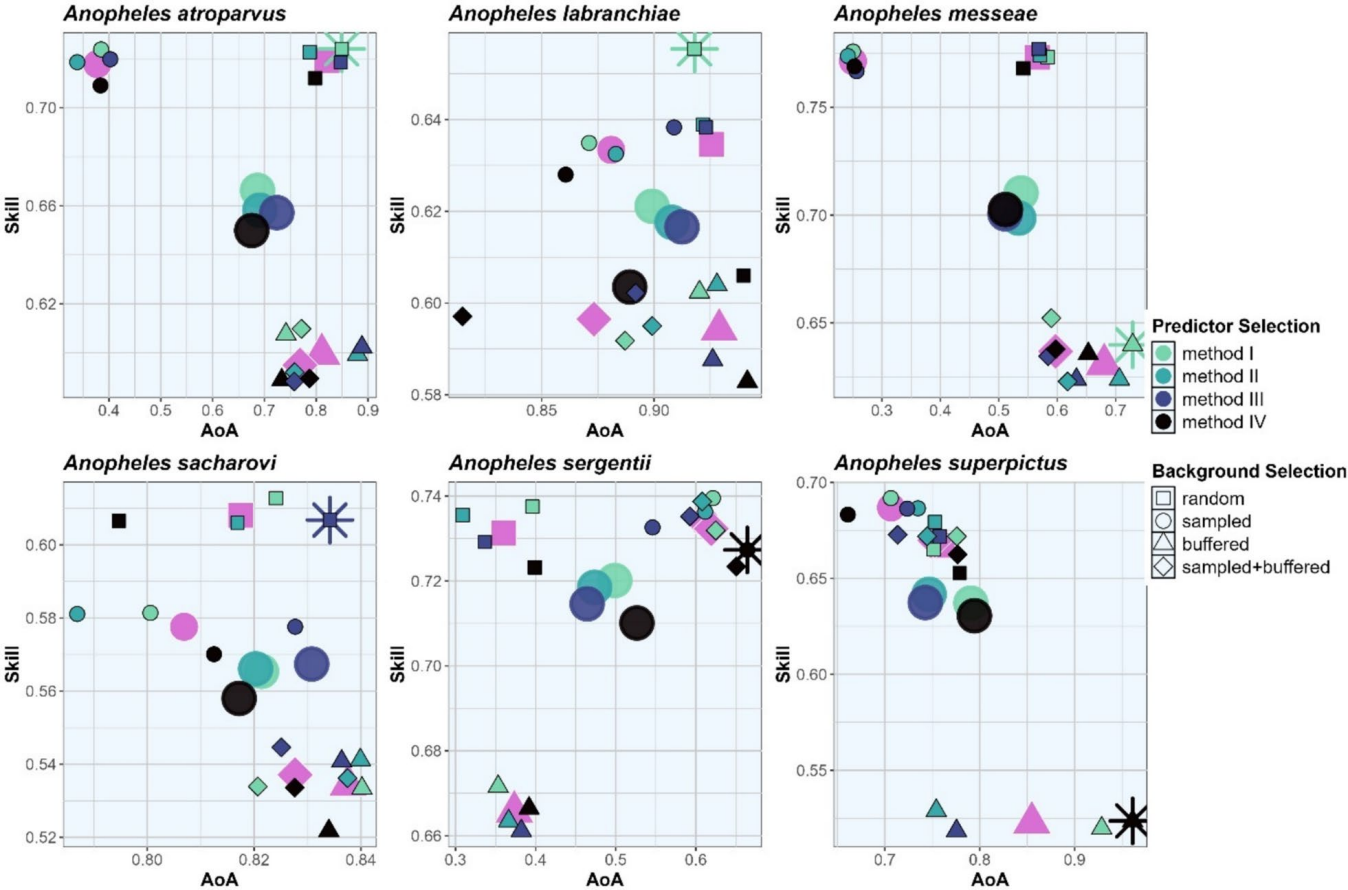
Overall model skill and area of applicability (*AoA*) of the established models for six different* Anopheles* species. Results are presented for each model setup (small symbols) and for the averaged model setup (large symbols) with respect to predictor (large colored points) and background selection (large magenta shapes) method. The best model performance is highlighted by an asterisk

Figure 1 presents the model skill and transferability (AoA) for the six *Anopheles* species considered in this study. It shows that the background point selection method (black symbols) has a greater effect on model skill and AoA than the predictor selection method (large colored circles). In general, random and sampled selection (squares and circles) provide higher skills, while buffered selection methods (triangles and diamonds) have a higher AoA. Considering both, skill and AoA, using all predictors or random background selection represents the best model for three *Anopheles* species and in combination for two species (*An. atroparvus*, *An. labranchiae*). For four species, the best model represents models with high skill and high AoA, while the best model of *An. messeae* and *An. superpictus* provides reduced skill in favor of higher AoA. Across all models and species, the skill is between 0.5984–0.8090 and the AoA is between 0.2418–0.9611, resulting in scores between 0.5760–0.8212. Over all species, *An. labranchiae* has the highest, and *An. messeae* the lowest score (Table 2). Results for all species and model setups are given in Additional file 2 Model evaluation: Tables S2.2.1–S2.2.6.

**Table 2:** Skill and transferability of the best boosted regression tree models for each* Anopheles* species

*Anopheles* species	Buffered	Sampled	Selection	TSS	AUC_SSB_	CK_SSB_	AoA	OMS^a^	SCORE^b^
*An. atroparvus*	No	No	I	0.8991	0.6950	0.5488	0.8492	0.7240	0.7891
*An. labranchiae*	No	No	I	0.9712	0.5500	0.4420	0.9180	0.6554	0.7976
*An. messeae*	Yes	No	I	0.9138	0.4754	0.3959	0.7291	0.6398	0.6859
*An. sacharovi*	No	No	III	0.9364	0.4980	0.3842	0.8342	0.6068	0.7294
*An. sergentii*	No	Yes	IV	0.9564	0.6390	0.4976	0.6642	0.7273	0.6964
*An. superpictus*	Yes	No	IV	0.9190	0.5986	0.2735	0.9611	0.5236	0.7739

*AoA* Area of applicability,* AUC*_*SSB*_ area under the curve with spatial sorting bias removed,* CK*_*SSB*_ Cohen’s kappa with spatial sorting bias removed,* TSS* True Skill Statistics

^a^The overall model skill (OMS) represents the average skill of TSS, AUC_SSB_ and CK_SSB_

^b^The SCORE summarizes OMS and transferability (AoA)

**Fig. 2:**
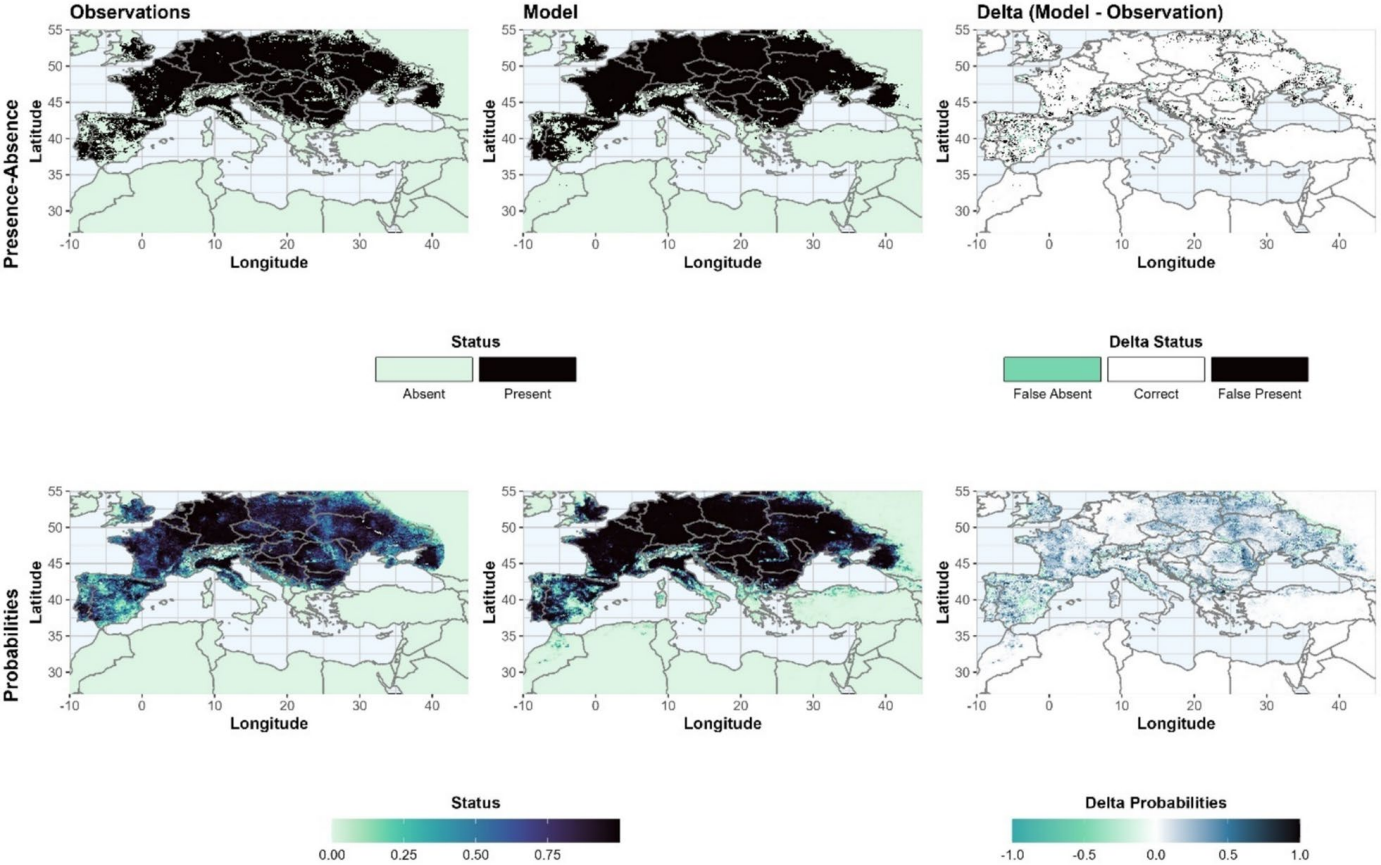
Presence-absence (top) and probabilities (bottom) for the observations (left) and the best model setup (middle) and the differences (right) for* Anopheles atroparvus* in 2010

For all species, the HSA of the best model setup is larger than the observed one (Additional file 2 Model evaluation: Figures S2.3.1–S2.3.6), but this applies especially to *An. messeae* where the HSA is 36.0% larger than the HSA of the observation (see Additional file 2: S2.3.3). The explanation for this is that the model identifies large parts of the southern edge as suitable, providing conditions similar to those in the presence areas of the observations. The model of *An. messeae* correctly represents 92.6% of the absences and 98.3% of the presences, with only 5.1% false presences or absences. In terms of HSA size, the modeled HSA of *An. atroparvus* is closest to the observed HSA (+ 9.0%), but 5.5% are false presences or absences. The lowest rate of false presences or absences can be observed for *An. labranchiae*, the species with the smallest HSA in our study region. In Fig. 2, observations, the results of the best model setup and the differences are shown for 2010 for *An. atroparvus*. In general, the model provides higher probabilities for the core of the observed HSA, but lower probabilities in southeastern Spain and at the eastern edge in Belarus and Ukraine. False absences are also concentrated in these regions and in mountainous areas, while false presences can be observed at the edges of the distribution area, where the observation represents a heterogeneous distribution of presences and absences.

### Changes within the HSA of *Anopheles* species between 2000–2020

**Fig. 3:**
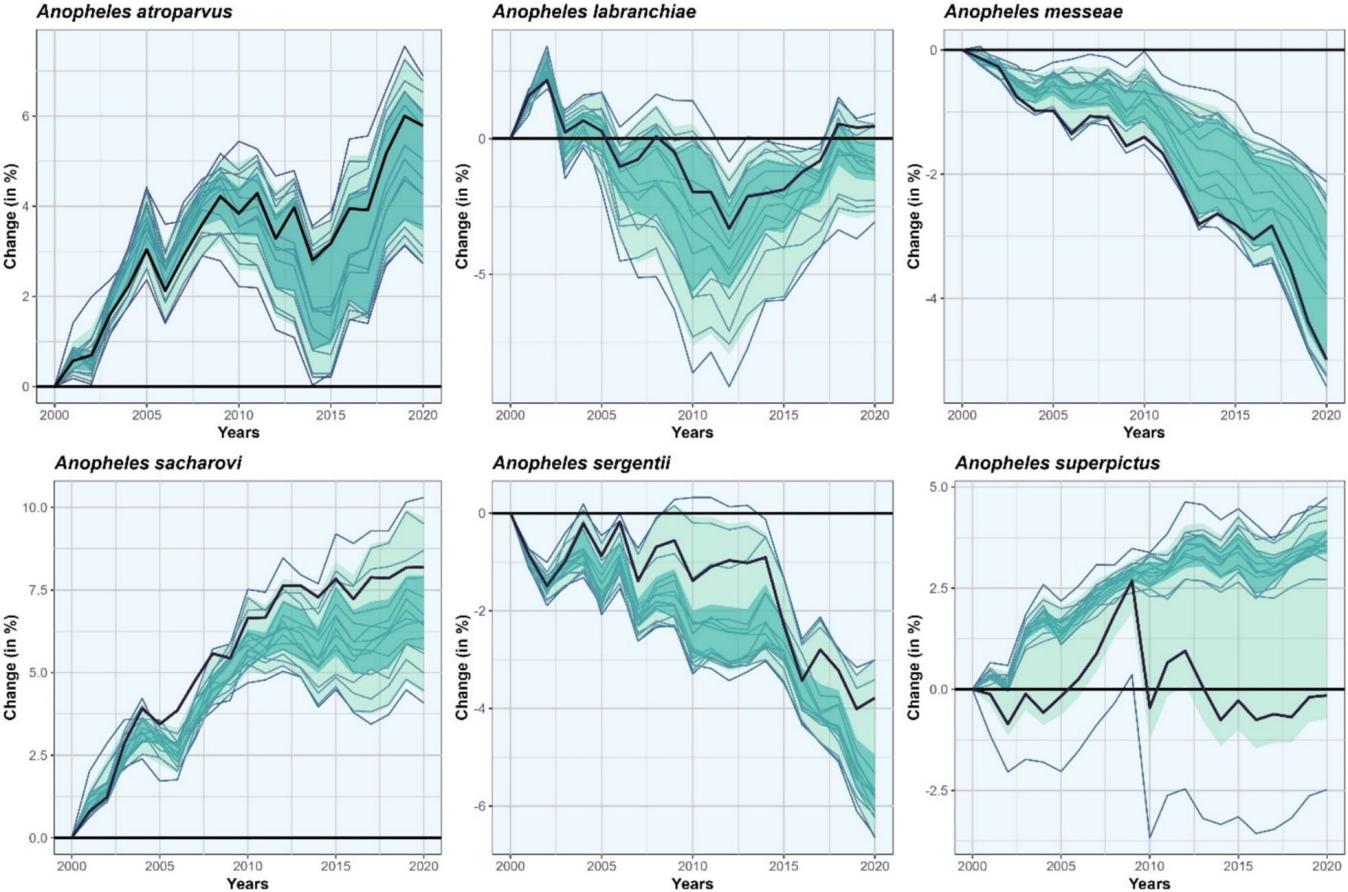
Changes in the habitat suitability area (HSA) of the* Anopheles* species considered in this study, between 2000 and 2020, with the HSA of 2000 as reference. Changes are shown for each model setup (blue lines) and the best model (thick dark lines). The interquartile range (25th–75th) is shaded in turquoise and the range between the 5th and 95th quartile is shaded in green

Changes in the HSA of the six different *Anopheles* species are shown in Fig. 3 for the entire MME. For *An. atroparvus* (+ 2.7 to + 6.9%) and *An. sacharovi* (+ 4.1 to + 10.3%) all models show an increase of the HSA, while the HSA of *An. messeae* (− 5.4 to − 2.1%) and of *An. sergentii* (− 6.7 to − 3.0%) are decreasing. For *An. labranchiae* (− 3.1 to + 0.9%) and *An. superpictus* (− 2.5% to + 4.7%) the MME does not show a consistent sign of change in HSA across the considered period. Considering only the best model setup, the largest increase in HSA between 2000 and 2020 is observed for *An. sacharovi* (+ 8.2%), while the largest decrease is observed for *An. messeae* (− 5.0%). For *An. superpictus*, the MME shows an opposite trend from 2008 onwards. While most models show a further increase, two models, including the best model, reach a turning point with a decreasing trend afterwards. Thus, the change of the best model is 3.8% below the median of the MME. For all other species, the MME shows a consistent trend with varying degrees of intensity.

### Reasons for change

**Fig. 4:**
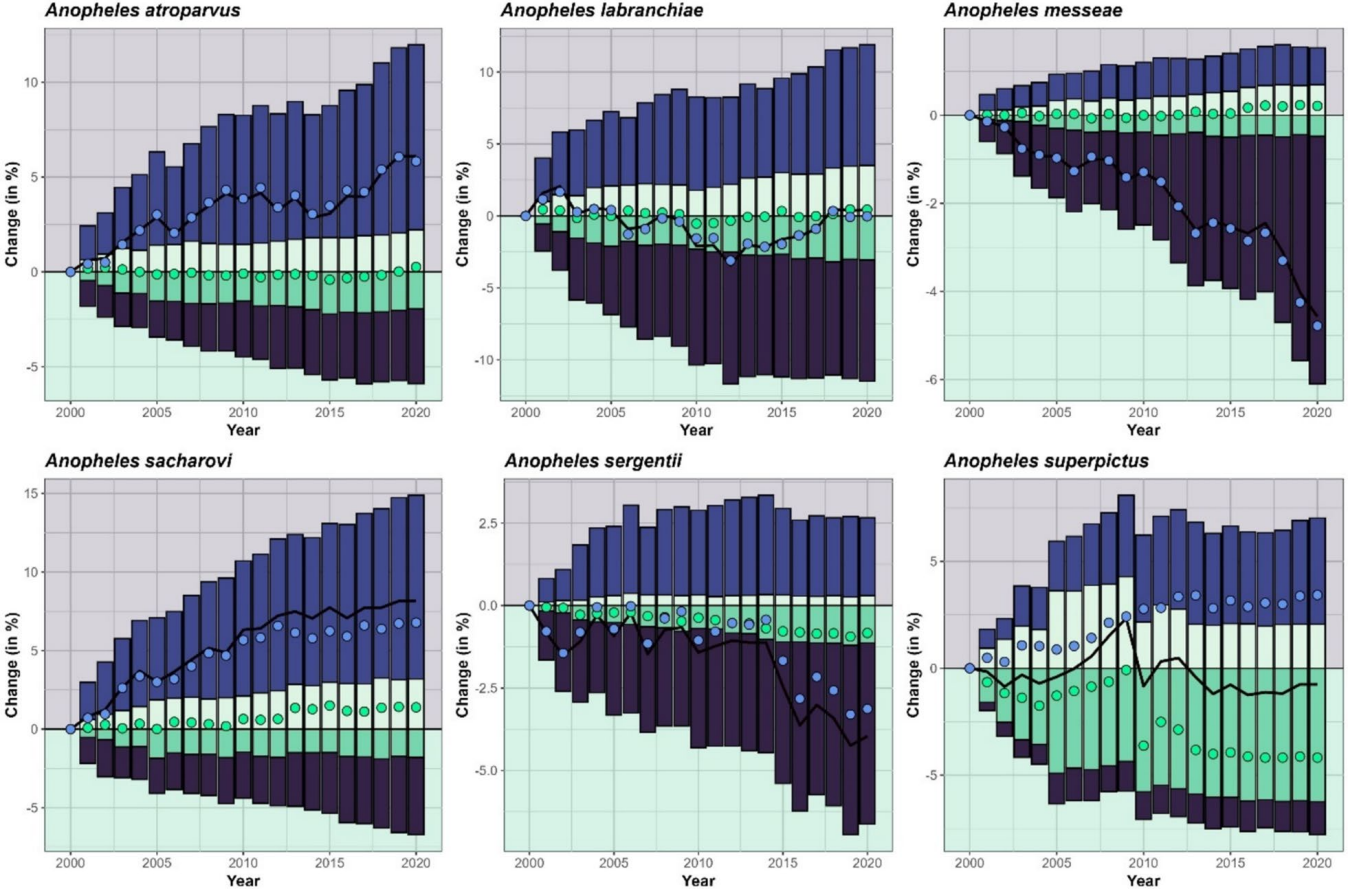
Changes in the habitat suitability area (HSA) between 2000 and 2020 due to climate (blue) and land-use (green) changes for different* Anopheles* species. Bars represent increases (light) and decreases (dark) modeled by means of the best model setup, and dots represent the respective net changes arising from both impact factors. The dark lines show the overall changes for each* Anopheles* species

With respect to the best model, the climate effect on changes of the species’ HSA was found to be much greater than the effect of land-use (Fig. 4). Only for *An. superpictus* is land-use seen to have a greater influence than climate. For *An. atroparvus* and *An. labranchiae*, the positive (i.e. expansion) and negative (i.e. shrinkage) effects of land-use on HSA almost cancel each other out (green dots), while for *An. messeae* and *An. sacharovi*, the expansion (light-green bars) exceeds the reduction (dark-green bars) due to land-use change, and vice versa for *An. sergentii* and *An. superpictus*. The influence of climate change on the development of the HSA ranges from 2.6- (*An. labranchiae*) to 5.6-fold (*An. sergentii*) higher than the influence of land-use; only for *An. superpictus* is the ratio in favor of land-use (1:1.3). Results for each species and model setup are given in Additional file 3 Reasons for change: Tables S3.1–S3.6.

The greatest influence of climate on HSA was observed for *An. labranchiae*, where 16.8% of the reference HSA in 2000 was found to change due to climate up to 2020. Since new presences (8.4%, light-blue bars in Fig. 4) and new absences (8.4%, dark-blue bars) due to climate change cancel each other out, the net change in the HSA for this species is almost zero (blue dots), but a spatial shift can be observed. *Anopheles sacharovi*, with an increase of 11.7% compared to a decrease of 4.9%, is the species that benefits most from climate change. In contrast, for *An. messeae*, a 0.8% expansion faces a 5.6% contraction due to climate change. In terms of land-use changes, *An. sacharovi* benefits the most (+ 1.4%), while *An. superpictus* shows the largest decrease (− 4.1%). In summary, the largest expansion in the HSA between 2000 and 2020 was observed for *An. sacharovi* (+ 8.2%), as climate and land-use changes favor expansion. The largest reduction in our study area was observed for *An. messeae* (− 4.6%). Although changes in land-use would favor the spread of the species, changes in climate lead to a sharp decline in the HSA.

**Fig. 5:**
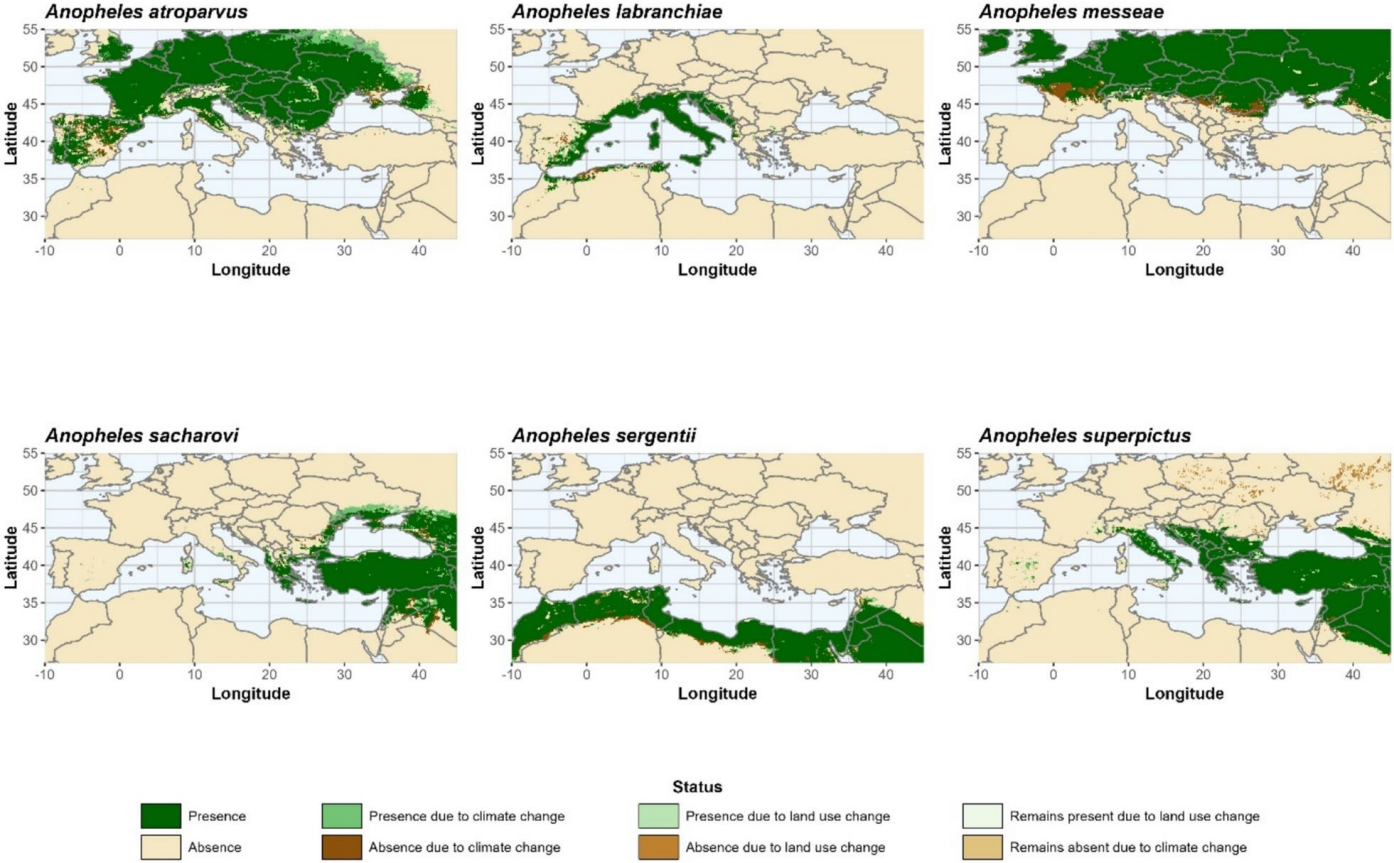
Habitat suitability area (HSA) and status changes for different* Anopheles *species between 2000 and 2020

Figure 5 shows the regional changes of the best model setup for each *Anopheles* species between 2000 and 2020 with respect to climate and land-use changes. For *An. atroparvus*, the expansion of the HSA due to climate change can be seen to mainly occur in a northeasterly direction along the Ukraine-Russian border and Belarus, while the foothills of Spain (Castilla and Leon, Aragon) and the coastal areas of the Sea of Azov (Southern Russia, Donezk, Cherson, Zaporizhzhya) provide the largest areas with unfavorable climate conditions in 2020. Land-use changes in the Coimbra region (Portugal) and between Seville and Granada (Spain) favor the establishment of *An. atroparvus* in the southwestern parts of the study area, northwest of Minsk (Belarus) and in the Charkiv region (Ukraine) in the northeast. In contrast, larger areas with land-use changes that prevent the establishment of *An. atroparvus* are found in Spain and south of Bordeaux in the west, and on the Crimean Peninsula in the east. Areas where the status is present or absent due to changes in land-use are found in Spain, Ukraine and southern Russia. In Spain, the RPLUC (remains present due to land-use change) status dominates, while areas with RALUC (remains absent due to land-use change) are observed in the eastern Ukraine.

For *An. labranchiae*, large areas in the western Mediterranean become climatologically favorable in 2020, but this is also predicted for the northern Balkans, southern Bulgaria and along the Turkish coast of the Black Sea. Unfavorable climatic conditions can be found in the immediate vicinity of Madrid and the adjacent areas to the south, as well as in the coastal areas of Algeria and the southern Balkans. Changes in the status due to land-use (presence or absence) are mainly concentrated in Spain, but not within clearly identifiable geographical clusters.

For *An. messeae*, a climatic expansion of HSA can be observed in the western parts of Ireland and the UK, the coastal regions of Germany, southern Russia and the Alps. Larger areas of retreat can be found on the southern edges of the original distribution area, especially in France, Serbia, Bulgaria and Romania. Changes due to land-use are scattered along the edges of the HSA of 2000, but not as coherent areas. Only on the Crimean Peninsula do land-use changes ensure that a larger area remains suitable for *An. messeae*, even though climatic conditions are becoming less favorable.

*Anopheles sacharovi* is climatically pushed in a northerly direction (Ukraine, southern Russia, Moldova), but the western Balkans, central and southern Italy and large parts of Syria and Iraq also offer climatically suitable conditions for establishment. Extended areas of unfavorable climatic conditions can also be observed in Iraq and Syria, as well as along the northern coast of the Black Sea (from Bulgaria to Georgia). Changes in land-use mainly favor the expansion of *An. sacharovi* in Ukraine and in isolated grid boxes in Italy and the Balkans, while the other land-use categories provide only scattered grid boxes around the HSA of 2000. Here, RPLUC is mainly observed in the southern adjacent regions, while RALUC represents more northern regions.

Small expansions of the HSA due to climate change can be observed for *An. sergentii* in Iraq, Syria, Libya and Morrocco, while an extensive reduction of the HSA area occurs at the southern edge of the original HSA. Land-use change has only a small impact on the HSA, with only one contiguous area in Lebanon providing favorable conditions due to land-use change in 2020, as well as some scattered grid boxes in the Western Mediterranean. Unfavorable land-use conditions are observed in Syria and Lebanon, along the Nile and in the northern region of Algeria. In the western parts of Syria, larger areas can also be observed where *An. sergentii* remains absent due to changes in land-use. In addition, some grid boxes in Sicily, Sardina and southern Spain provide conditions that favor the establishment of *An. sergentii*.

Although *An. sacharovi* and *An. superpictus* share approximately the same HSA, the expansion of *An. superpictus* is in a different main direction. In particular, a westward expansion of the HSA of *An. superpictus* can be observed especially in the Balkans, Italy and Spain, but some areas in Saudi Arabia and along the southern coast of the Black Sea also offer climatologically suitable conditions. In Saudi Arabia, Jordan and Syria, large areas are becoming unfavorable due to climate change, but the coastal regions of the Aegean Sea also provide unsuitable climatic conditions in 2020. Land-use change improves conditions for the establishment of *An. superpictus*, especially in Italy and northwestern Turkey, but set back conditions in the northeastern parts of the study area (Ukraine, southern Russia).

### Most important and change-driving predictors

Compared to the climatological predictors, the main land-use predictors were found to play a minor role. 6.2% of the land area has land-use variables as the MIP for *An. atroparvus*, while only 0.4% has land-use variables as MIP for *An. superpictus*. Considering only land-use variables, the category Urban (U) represents the MIP for *An. atroparvus* (35.9% land area of the study area) and *An. messeae* (29.8%), deciduous shrubs (DS) for *An. labranchiae* (34.3%) and *An. superpictus* (30.8%), non-irrigated crops (NIC) for *An. sacharovi* (26.4%) and coniferous shrubs for *An. sergentii* (35.6%). NIC and DS are in the top three land-use categories for five of the six species. Further important land-use categories are irrigated crops (IC) and temperate deciduous trees (TDT). Besides the representation of the natural reservoirs, no spatial structure can be observed for most of the land-use predictors. Only for NIC and IC can a tendency be observed: while NIC is selected as the MIP mainly in Eastern Europe and, to some extent in Spain (*An. messeae*), IC represents the MIP in Spain, Italy, Greece, Turkey and the Middle East. As land-use change has only a marginal effect on the HSA of *Anopheles* species in this study, only the MIPs related to climate change are discussed below, but figures and results for the land-use related MIPs are provided in Additional file 4 Most important predictors: Figure S4.2.

#### *Anopheles atroparvus*

**Fig. 6:**
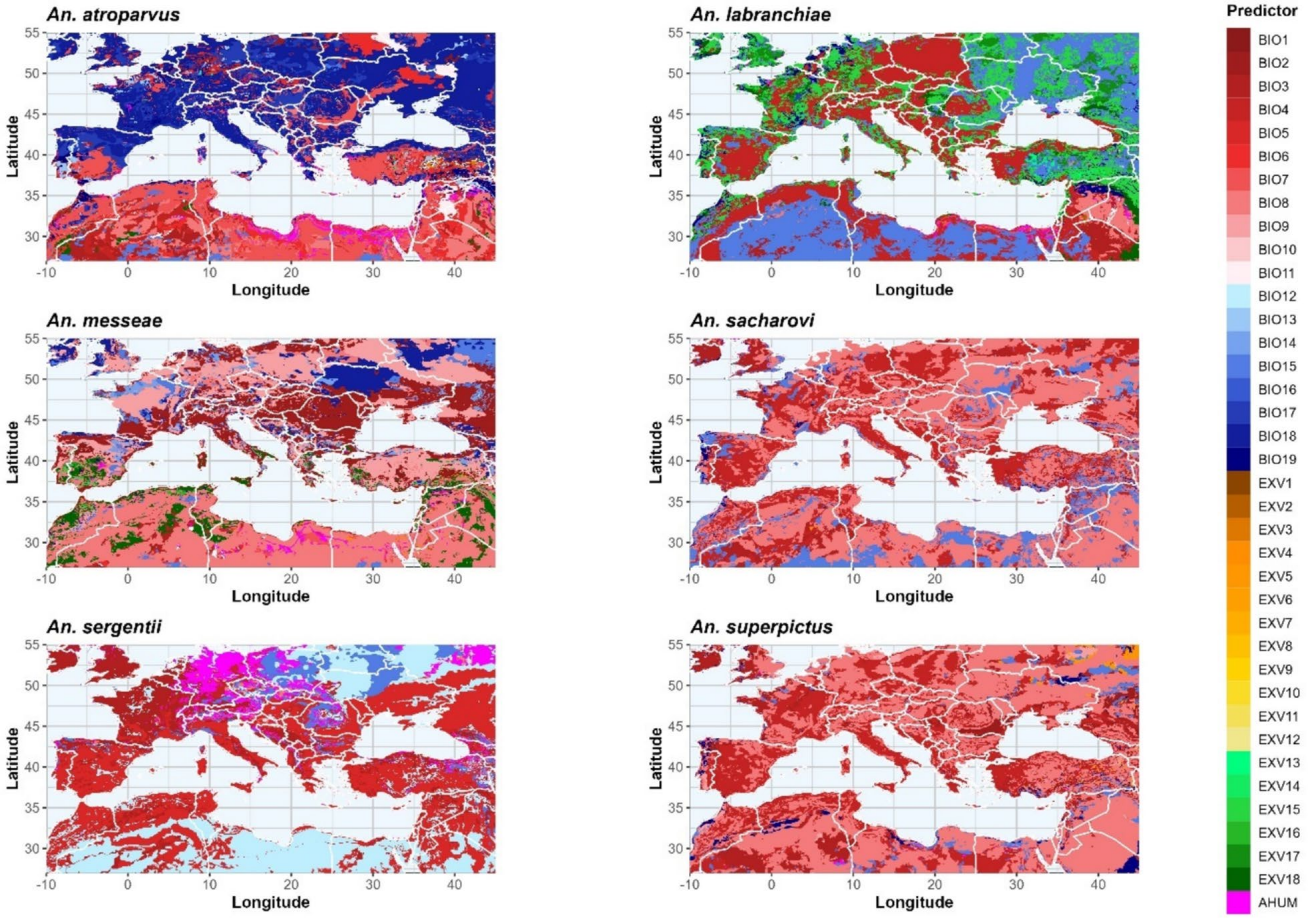
Most important climatic predictors. Red coloration represents temperature-related BioClim variables, blue colors represent precipitation-related BIO variables, yellow colors represent temperature-related extremes, green colors represent precipitation-related extremes and pink colors represent AHUM. AHUM Absolute humidity; BIO, Bioclimatic variable; EXV, extreme variable

The MIP for the establishment of *An. atroparvus* is precipitation of the warmest quarter (BIO18, 33.5%), followed by the annual temperature range (BIO7, 17.0%) and the precipitation of the driest quarter (BIO17, 11.7%). The HSA is mainly driven by precipitation predictors (55.7%) and mean values (BIOs and AHUM, 99.1%) are more crucial than extremes. Figure 6 (top left) shows that the study area is divided into two parts, with the northern part showing mainly precipitation-related predictors as MIP and the southern part depending more on temperature-related predictors. North of the 40th latitude, BIO18 dominates as the MIP, but also larger areas with BIO17 representing the MIP can be observed in Spain, eastern Germany and Hungary. BIO7 represents the MIP in southern and central Spain, Romania, western Turkey, the Levant region and northern Africa, but not directly along the coasts.

**Fig. 7:**
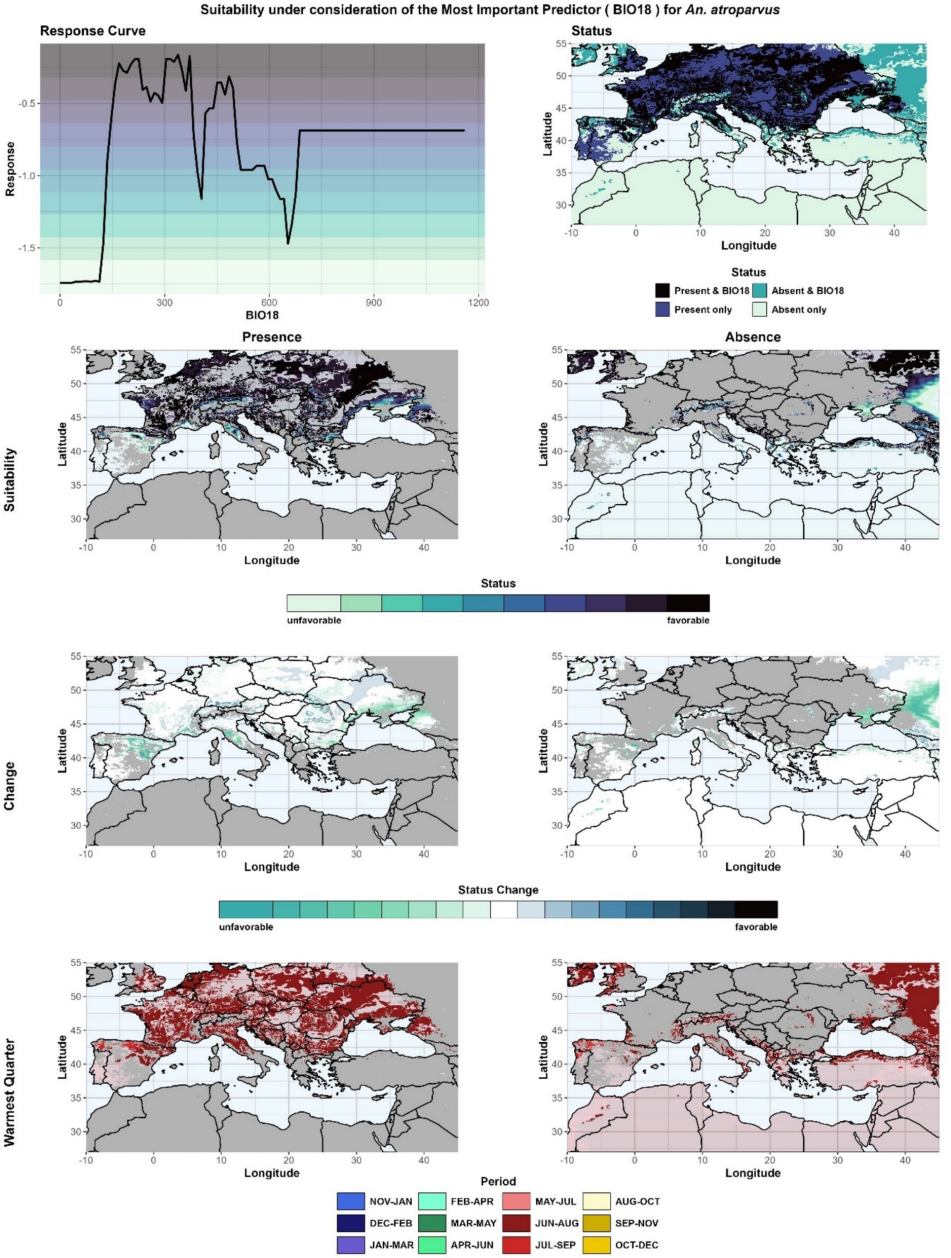
Suitability under consideration of the most important predictor for* Anopheles atroparvus*. The response curves of the most important predictor (MIP) and the status are presented at the top. Below, suitability maps are given for the presence (left) and absence areas (right) (second row from top), changes in suitability (third row from top) and the reference quarter representing the warmest quarter (bottom row). BIO, Bioclimatic variable

BIO18 also represents the most important predictor responsible for the changes (MIPC; Fig. 7). The period from June to August is mainly the warmest quarter, and only in some regions is the warmest quarter shifted by 1 month (JUL-SEP). Thus, the reference quarter (i.e. warmest quarter) represents the peak season of the species' life-cycle dynamics (Fig. 7, bottom), and no seasonal aspects need to be considered. The response curves show that the optimum range is between 160 and 375 mm of precipitation and that unfavorable conditions exist below 115 mm (Fig. 7, top left) and to a lesser extent above 650 mm. In 2020, the most favorable areas can be seen to fall within the range extending from the French Atlantic coast to western Russia (Fig. 7, second row left); however, also outside the range, favorable areas can be observed (Fig. 7, second row right). Although present, unsuitable conditions due to BIO18 can be observed in the western Mediterranean, where a decrease in precipitation during the warmest quarter leads to less favorable conditions (Fig. 7, third row left). Moreover, these regions correspond to areas where climate change is causing species retreat. Improved conditions are primarily found in the foothills of mountainous regions and to the east of the Black Sea. These regions have high precipitation levels of over 650 mm, but with a decreasing trend, resulting in more favorable conditions for the establishment of *An. atroparvus*. The presence of low-level precipitation territories to the east of the Sea of Azov serves to impede any further eastward expansion (see Fig. 7, third row, right), while the prevailing conditions to the north are conducive to continued spread.

#### *Anopheles labranchiae*

For *An. labranchiae* (Fig. 6, top right), temperature seasonality (BIO4, 30.2%) is the MIP, especially in central and southern Europe, while precipitation seasonality (BIO15, 29.4%) is an important factor in Eastern Europe and North Africa. In Europe, the Levant and along the coast of Northwest Africa, larger coherent areas can be observed where the longest period of precipitation > 20 mm (EXV15, 21.0%) represents the MIP. *Anopheles labranchiae* represents the species which depends most on extreme (29.9%) and precipitation (63.6%) variables.

The optimum range for BIO4 is mainly observed in regions where the climate is determined by the Atlantic and along the southeastern Mediterranean coast. Within the presence area, the Po Valley shows unfavorable conditions with respect to BIO4, and conditions became worse between 2000 and 2020. Thus, other factors counteract the development of BIO4 in this area, and the establishment of *An. labranchiae* is still possible. In general, the potential for the spread of *An. labranchiae* is limited. Limited improvements in suitability restrict expansion to areas where favorable conditions already exist (Additional file 4: S4.2).

#### *Anopheles messeae*

*Anopheles messeae* (Fig. 6, mid left) mainly depends on mean values (91.3%), and temperature (76.8%) is more important than precipitation. The MIPs are the mean temperature of the wettest (BIO8, 28.1%) or the driest (BIO9, 22.9%) quarter, followed by the mean diurnal temperature range (BIO2, 22.1%). BIO8 mainly represents the MIP in North Africa and the Middle East, while BIO9 is more important in the northern parts of the study area. BIO2 as MIP can be observed along the Mediterranean and Black Sea coastlines.

BIO8 mainly represents a limiting factor in the southern parts of the study area where winter is the wettest season and therefore temperatures are generally lower. In contrast, favorable conditions for the wettest quarter can be observed from Eastern Germany to Russia, but here BIO8 is mainly a minor predictor and BIO2 is more important. This area has the highest rainfall in summer, and the HSA of *An. messeae* highly corresponds to these areas (81.8%). Differences are limited to the southern edge, where the precipitation maximum also occurs in summer but the species is absent, and in some western parts, where the precipitation maximum does not occur in summer, but the species is present (Additional file Most important predictors: Figure S4.3.1). The mean diurnal temperature range is the MIP responsible for changes due to climate. BIO2 represents the MIP especially in the border area between presence and absence, and the most favorable areas with respect to BIO2 cover approximately the HSA of *An. messeae*.

#### *Anopheles sacharovi*

Means (99.6%) and temperature-related variables (85.0%) represent the MIPs for *An. sacharovi* (Fig. 6, mid right). More than 50% of the grid boxes have BIO8 as the MIP, and coherent regions can be found throughout the study area. BIO4 (25.5%) is mainly observed in southern and central Europe and BIO15 (12.7%) represents the MIP in the southern to southeastern parts of the study area.

In contrast to *An. messeae*, lower temperatures within the wettest quarter favor the establishment of *An. sacharovi*. Favorable conditions for the MIP are observed in the recent presence areas, as well as in Western Europe and Italy. It is interesting to note that in most areas of the western study area where BIO8 is in the optimal range, BIO8 does not represent the MIP for *An. messeae*. Improvements in suitability along the northern edge of the HSA are the main drivers for the northward spread of *An. sacharovi* as BIO8 represents the MIP here. The wettest quarter within the presence area is mainly in the winter or spring, but some areas in the western parts have their maximum precipitation in the fall and some in the northern parts have their maximum precipitation in the summer. Winter and spring maxima can also be observed in Spain and France where the MIP is within the optimal range, but the species is still absent (Additional file 4 Most important predictors: Figure S4.4.1).

#### *Anopheles sergentii*

The MIP for *An. sergentii* (Fig. 6, bottom left) is the maximum temperature of the warmest month (BIO5, 37.6%), followed by the mean annual precipitation (BIO12, 28.5%) and isothermality (BIO3, 13.8%). Means (92.3%) are more important than extremes, and the HSA of the species depends on both temperature- (56.9%) and precipitation-related (43.1) variables. BIO5 is dominant in the southwestern and eastern parts of the study area, while BIO12 and BIO3 have the greatest impact on suitability in North Africa and in oceanic climates, respectively. In addition, *An. sergentii* is the species where absolute humidity is most frequently selected as MIP (7.7%), especially in Central Europe.

BIO5 is mainly responsible for the changes to present due to climate change. Outside of the presence area, favorable conditions are observed in adjacent areas as well as in large parts of the Euro-Mediterranean regions. The northernmost favorable areas according to BIO5 are along the French Mediterranean coast, in the Po Valley and in southern Russia. Within these areas, BIO5 also represents the MIP. Due to climate change, many regions have become more favorable for the establishment of *An. sergentii*, providing many opportunities for its spread (see Additional file 4 Most important predictors: Figure S4.5.1).

#### *Anopheles superpictus*

With increasing continentality, BIO8 (54.1%) represents the MIP for *An. superpictus* (Fig. 6, bottom right). In contrast, the second MIP (BIO4, 25.6%) shows a gradient in the opposite direction, while BIO3 (9.2%) is dominant in the northwestern parts of the study area and in Algeria. The effect of precipitation variables (5.8%) is almost negligible and habitat suitability of *An. superpictus* depends mainly on the means of temperature-related variables (93.4%).

BIO8 represents the MIP especially for areas where *An. superpictus* is predicted to become absent due to climate change. Since the absence areas to the north have the highest precipitation in summer, temperatures are too high for further northward expansion and the spread is limited to westward directions. Although conditions deteriorated in these regions between 2000 and 2020, favorable conditions can still be found in the western Euro-Mediterranean region. Winter and spring are the wettest seasons in the presence area, and the corresponding regions of France and the Iberian Peninsula also have the highest rainfall during these seasons (Additional file 4 Most important predictors: Figure S4.6.1).

BIO4 is the predictor that is primarily responsible for the changes to present due to climate change. In general, the higher BIO4, the higher the suitability for establishment. BIO4 represents the MIP especially in the western parts of the HSA, and in large parts of Spain and France. In terms of BIO4, the most favorable areas are in the eastern parts of the study region and in the desert regions of North Africa, but conditions for establishment have improved throughout the study region between 2000 and 2020 (Table 3).

**Table 3:** Most important predictors and most important predictors for change for the different* Anopheles* species

*Anopheles* species	Status^a^	MIP©)^b^	%^c^	Time span of study		∆^d^	∆ Res^e^
				2000	2020		
*An. atroparvus*	*P*	*BIO18*	*57.1*	*238.5*	*223.0*	*− 15.5*	*− 0.0331*
	*A*	*BIO18*	*19.6*	*79.8*	*74.3*	*− 5.4*	*− 0.0398*
	PCC	BIO18	50.2	255.4	235.3	− 20.1	0.0763
	ACC	BIO18	55.4	160.9	137.9	− 23.1	− 0.5044
	PLUC	U	46.6	1.5	1.7	+ 0.2	0.0724
	ALUC	U	30.4	37.1	33.3	− 3.8	− 0.0742
	RPLUC	U	42.6	1.4	1.6	+ 0.2	0.0676
	RALUC	U	34.4	37.2	35.5	− 1.7	− 0.0318
*An. labranchiae*	*P*	*BIO4*	*54.9*	*666.8*	*690.3*	* + 23.5*	*− 0.2633*
	*A*	*BIO4*	*28.1*	*793.2*	*802.2*	* + 9.0*	*− 0.1173*
	PCC	BIO4	54.9	676.3	700.8	+ 24.5	− 0.2966
	ACC	BIO4	61.8	661.3	688.3	+ 27.0	− 0.2159
	PLUC	TBET	33.7	8.2	10.5	+ 2.3	0.1451
	ALUC	TBET	28.1	2.9	2.8	− 0.1	− 0.0391
	RPLUC	TBET	28.2	7.4	10.1	+ 2.7	0.0466
	RALUC	TBET	45.1	3.4	3.2	− 0.2	− 0.0017
*An. messeae*	*P*	*BIO8*	*4.3*	*15.3*	*16.2*	*+ 0.9*	* + 0.1817*
	*A*	*BIO8*	*42.0*	*11.1*	*11.7*	* + 0.7*	* + 0.0550*
	PCC	BIO2	46.4	6.6	6.7	+ 0.1	0.2218
	ACC	BIO2	47.4	8.0	8.2	+ 0.2	− 0.1548
	PLUC	U	33.7	5.1	5.5	+ 0.4	0.0106
	ALUC	DS	32.8	6.0	7.6	+ 1.6	− 0.0000
	RPLUC	C4	33.6	0.6	0.9	+ 0.3	0.0505
	RALUC	U	24.2	24.5	23.6	− 0.9	− 0.0361
*An. sacharovi*	*P*	*BIO8*	*47.4*	*9.2*	*9.9*	*+ 0.7*	*− 0.0294*
	*A*	*BIO8*	*53.9*	*13.4*	*14.1*	*+ 0.8*	*− 0.0894*
	PCC	BIO8	68.7	14.3	15.2	+ 0.9	0.0024
	ACC	BIO8	60.8	11.0	11.8	+ 0.8	− 0.4539
	PLUC	IC	45.8	4.9	6.6	+ 1.7	0.0366
	ALUC	IC	50.0	6.9	5.4	− 1.5	− 0.2009
	RPLUC	IC	49.0	3.0	8.2	+ 5.2	0.3120
	RALUC	IC	57.5	6.0	6.1	+ 0.1	− 0.0470
*An. sergentii*	*P*	*BIO5*	*47.2*	*35.2*	*36.0*	* + 0.8*	* + 0.0695*
	*A*	*BIO5*	*35.3*	*27.0*	*28.1*	*1.1*	* + 0.0821*
	PCC	BIO5	50.6	34.9	35.6	+ 0.7	0.1272
	ACC	BIO12	64.4	100.6	92.6	− 8.0	− 0.1336
	PLUC	CS	74.4	17.9	16.5	− 1.4	0.2328
	ALUC	CS	97.4	7.2	3.9	− 3.3	− 0.3171
	RPLUC	CS	60.0	14.5	13.6	− 0.9	0.0949
	RALUC	CS	97.0	9.1	5.4	− 3.7	− 0.6517
*An. superpictus*	*P*	*BIO8*	*52.8*	*8.3*	*9.0*	* + 0.7*	*− 0.1667*
	*A*	*BIO8*	*54.4*	*13.8*	*14.5*	* + 0.8*	*− 0.2016*
	PCC	BIO4	59.1	725.1	745.8	+ 20.7	0.6047
	ACC	BIO8	65.8	12.1	12.8	+ 0.7	− 0.1795
	PLUC	C4	21.7	4.4	4.9	+ 0.5	0.0399
	ALUC	C3	45.3	0.7	1.4	+ 0.7	− 0.0022
	RPLUC	DS	29.8	10.4	11.7	+ 1.3	0.0066
	RALUC	DS	29.6	8.4	9.9	+ 1.5	− 0.2486

*MIP(C) *Most important predictor (for change)

^a^P, Present; A, Absent; PCC, change to present due to climate change; ACC, Change to absent due to climate change; PLUC, change to present due to land-use change, ALUC, change to absent due to land-use change; RPLUC, remains present due to land-use change; RALUC, remains absent due to land-use change

^b^The MIP(C) (in italics) is analyzed for the entire presence (P) and absence (A) areas, the MIPC (normal font) is analyzed only for the respective areas with status change (PCC-RALUC). BIO, Bioclimatic variable; CS, coniferous shrubs; C3, C3 grass; C4, C4 grass; DS, deciduous shrubs; IC irrigated crops; TBET, temperate broadleaf evergreen trees; U, urban

^c^The fraction of grid boxes (in percentage, %) where the variable represents the MIP(C)

^e^The difference in the mean values (∆) of the variable between 2020 and 2000

^e^The difference in the response (∆ Res) between 2020 and 2000

## Discussion

Sinka et al. [49] emphasize that the predictive data and maps that we used as reference in the present study are not perfect representations of the true distributions of the *Anopheles* species included in our study. These authors indicate that variations in sampling methodology and collection location, as well as limited or missing data across large areas, affect the accuracy of final predictions. We therefore note that our analysis is not intended to be a perfect representation of the true species distributions.

The present study provides a statistical approach to assess the HSA evolution of six different *Anopheles* species between 2000 and 2020. Different methods of selecting predictors and background points were tested, and changes in HSA were attributed to either climate or land-use change. Finally, within the groups of climate and land-use predictors, we identified the MIP for each species as well as the MIP responsible for the changes. The present study provides some interesting findings, with our analyses showing that: (i) background selection has a greater impact on skill and transferability than within-model predictor selection; (ii) statistical methods can be useful to identify important predictors for projecting the HSA; (iii) climate change is mainly responsible for changes in the HSA of the species; and (iv) the MMEs of three species show on average an increase in the HSA, two show a decrease or shift and one shows no significant change, but for *An. superpictus*, the projections of the MME diverge substantially.

The relatively small effect of within-model predictor selection on model skill and transferability is due to the successive nature of the selection process, so that the most important predictors, as determined by the total set of predictors, are not eliminated in subsequent steps. Thus, the effect of within-model predictor selection on skill is only marginal. It is likely that considering pre-model predictor selection based on expert knowledge would have a greater impact on skill and transferability. However, especially when the model is used for projections, the elimination of unrelated predictors is crucial. In contrast, background selection is performed prior to model calibration and is therefore independent of the model calibration process. Background selection had a large impact on the transferability of the established models. In agreement with Barbet‐Massin et al*.* [70], we employed a substantial quantity of pseudo-absences and an equivalent number of presence points. Regarding transferability, recent literature does not recommend one specific method but rather emphasizes the need for checking (e.g. [71, 72]). Wenger and Olden [73] stated that complex models are less transferable than parsimonious models. In our study, this is only true for the best models of half of the species we investigated, but not in general. However, this result supports our approach of eliminating predictors during the model setup. Furthermore, in agreement with our study, there is a general consensus that the model should be calibrated and validated using spatially or temporally separated datasets [71]. Yates et al. [72] further emphasize that the transferability of the models depends on various factors, including, for example, taxon-specific or trait-specific factors, or it depends on the quality and resolution of the data. In the present study, transferability was assessed using an approach used by Meyer and Pebesma [69] that determines whether similar environmental conditions in the calibration and validation periods/areas are present. Thus, the approach of Meyer and Pebesma [69] only considers transferability due to environmental conditions, neglecting other species-specific factors, such as adaptability to new environments (which can hardly be assessed). In our study, we also observed a relationship between background point selection and transferability. Depending on the background points selected, transferability may be limited, especially within large study areas where different climate characteristics are present. Here, we used only 17.7% of the study area for model calibration, with half of the points restricted to presences. Thus, under-representation of certain climates cannot be ruled out, resulting in large differences in the AoA (compare Fig. 1). For large study areas like the Euro-Mediterranean region we recommend increasing the number of background points or selecting background points within spatial clusters representing similar zones to enhance model transferability. Barbet‐Massin et al*.* [70] also emphasize the importance of using geographically and environmentally stratified samples of pseudo-absences with machine-learning tools such as BRTs.

With respect to predictor selection, we have shown that statistical methods can be useful to identify important predictors for projecting HSA. However, we can also see that for some species statistical methods alone are not sufficient and the use of expert knowledge, where available, is strongly recommended. However, for some of the species presented here, expert knowledge is limited. Bertola et al. [19] performed a systematic review of what is known about* Anopheles* species, and the results are partly contradictory. Therefore, we decided to apply solely statistical predictor selection. That statistical selection alone is not sufficient for all species is particularly true for *An. superpictus*, where the temperature of the wettest quarter (BIO8) was identified as the MIP. The HSA of the species mainly coincides with the area with the highest seasonal rainfall in winter. The optimum range for BIO8 therefore includes temperatures between 2.9 °C and 9.8 °C, with suitability rapidly decreasing at higher temperatures. Given the extensive HSA of *An. superpictus* across Italy, Greece, Turkey and the Levant, it can be inferred that this species is not limited to cold environments. Consequently, the observed correlation between BIO8 and the HSA of *An. superpictus* is likely to be a pseudo-correlation. It is possible that the establishment of *An. superpictus* depends on winter temperatures or precipitation, but neither BIO11 nor BIO19 are identified as the MIP for many grid boxes. To confirm whether the relationship between HSA and BIO8 is a pseudo-correlation, the results for the MIP should be checked after re-running the model with an updated set of predictors where BIO8 has been removed. If either BIO11 or BIO19 does not replace BIO8 as MIP, the relationship between BIO8 and the HSA of *An. superpictus* represents a pseudo-correlation.

The illogical selection of BIO8 as MIP in specific cases highlights the need for further discussion about using interactive bioclimatic variables for HSA modeling. Some authors recommend excluding all interactive variables from the predictor dataset [74], while other studies point out that these variables represent an indispensable added value [75]. We argue that interactive variables are valuable only when seasonal shifts of the respective quarters or months within the study area/period are small. Otherwise, we recommend using fixed quarters or months, as already done by Hertig [63].

Regarding the question of whether climate or land-use is mainly responsible for changes in HSA, our analyses have shown that shifts in climatic conditions provide a higher contribution to modeled changes in HSA compared to land-use shifts. On average, over 75% of the changes recorded in this study can be traced back to climate. *Anopheles sergentii* in particular is very sensitive to climate change (MIN: 82.4%), while two models of *An. superpictus* are very sensitive to land-use change (MAX: 72.7%). However, we should be aware of these findings with respect to *An. superpictus,* as this may also be the result of the strong impact of BIO8 that limits the impact of climate change. These results contradict the theory of Lambin et al. [25] in which land-use is the main driver of the recent global spread of mosquitoes and MBDs. This outcome can be attributed to the minimal alterations in land-use that have occurred within the study region, as substantial changes in land-use were implemented decades ago. Thus, land-use changes in the Euro-Mediterranean region primarily occur on a local scale, a level of detail that cannot be captured by the resolution of our datasets. This conclusion is supported by studies with higher spatial resolution showing a stronger link between land-use and HSA (e.g. [25, 76, 77]). In addition, the maximum daily flight distance of the mosquitos [78] is substantially smaller than the grid resolution for some species. Thus, we can only assume that a certain grid cell is favorable or unfavorable for establishment, but not whether it has been actually colonized or whether it will be colonized in the future. It is important that we keep in mind that within each grid box, favorable and unfavorable biotopes can coexist. Similar results have also been reported by Merkenschlager et al. [57] for *Aedes albopictus* at the same spatial resolution. Thus, we found strong indications that the importance of land-use depends on the spatial resolution, with the higher the resolution, the more important features of the species habitat can be recorded. However, although the spatial resolution of our data is relatively coarse, we could assign almost 25% of the changes to land-use.

## Conclusions

In our study, we analyzed changes in the HSA for six *Anopheles* species within the period 2000–2020 and investigated the reasons and main drivers for change. Due to the lack of expert knowledge on the favorable conditions, we developed a purely statistical model to assess the HSAs of six *Anopheles* species. However, taking the presented results into consideration, we are unable to recommend one specific background or predictor selection approach since no method is generally superior to any of the others. With the exception of *An. superpictus*, where pseudo-correlations question the validity of some models, our study provides reasonable interactions between climate, land-use and habitat suitability. As Hertig [63] underscores, there is a dearth of comparable studies examining shifts in the distribution of the *Anopheles* vectors in the Euro-Mediterranean region. In this context, our study offers a significant contribution to the understanding of climate and land-use impacts on *Anopheles* species habitat suitability patterns within this region.

## Supplementary Information

Below is the link to the electronic supplementary material.

Additional file 1 Predictor variables: File S1.1. Bioclimatic variables. File S1.2. Extreme variables. File S1.3. Land-use variables. Additional file 2 Model evaluation: File S2.1. Predictor selection. File S2.2. Skills and scores. File S2.3. Comparisons between best model setup and observations. Additional file 3 Reasons for change: File S3.1. *An. atroparvus*. File S3.2. *An. labranchiae*. File S3.3. *An. messeae*. File S3.4. *An. sacharovi*. File S3.5. *An. sergentii*. File S3.6. *An. superpictus*. Additional file 4 Most important predictors: File S4.1. *An. atroparvus*. File S4.2. *An. labranchiae*. File S4.3. *An. messeae*. File S4.4. *An. sacharovi*. File S4.5. *An. sergentii*. File S4.6. *An. superpictus*.

## Data Availability

Data supporting the main conclusions of this study are included in the manuscript.

## Abbreviations

A: Absence
ACC: Change to absent due to climate change
AHUM: Absolute humidity
ALUC: Change to absent due to land-use change
AoA: Area of applicability
BIO: Bioclimatic variable
BRT: Boosted regression trees
BSS: Brier skill score
CK: Cohen’s kappa
CLU: Constant land-use
DS: Deciduous shrubs
EXV: Extreme variables
HSA: Habitat suitability area
IC: Irrigated crops
LP: Longest period
MAP: Malaria Atlas Project
MBD: Mosquito-borne diseases
MIP(C): Most important predictor (for change)
MME: Multi-model ensemble
ND: Number of days
NIC: Non-irrigated crops
OMS: Overall model skill
P: Presence
PCC: Change to present due to climate change
PLUC: Change to present due to land-use change
RALUC: Remains absent due to land-use change
REF: Reference
RPLUC: Remains present due to land-use change
SSB: Spatial sorting bias
TDT: Temperate deciduous trees
TSS: True Skills Statistics
U: Urban
VLU: Varying land-use
