# Supplementary material for: Changes in the recent habitat suitability of Euro-Mediterranean Anopheles species due to land-use and climate
Source: Parasit Vectors. 2025 Nov 27;18:500. doi: 10.1186/s13071-025-07115-0 (PMC12681145; doi:10.1186/s13071-025-07115-0)
Supplement: Supplementary file 1 — Additional file 1 Predictor variables: File S1.1. Bioclimatic variables. File S1.2. Extreme variables. File S1.3. Land-use variables. Additional file 2 Model evaluation: File S2.1. Predictor selection. File S2.2. Skills and scores. File S2.3. Comparisons between best model setup and observations. Additional file 3 Reasons for change: File S3.1. An. atroparvus. File S3.2. An. labranchiae. File S3.3. An. messeae. File S3.4. An. sacharovi. File S3.5. An. sergentii. File S3.6. An. superpictus. Additional file 4 Most important predictors: File S4.1. An. atroparvus. File S4.2. An. labranchiae. File S4.3. An. messeae. File S4.4. An. sacharovi. File S4.5. An. sergentii. File S4.6. An. superpictus. [file 13071_2025_7115_MOESM1_ESM.docx]

**Supplementary**

**S1 Predictor Variables**

S1.1 Bioclimatic variables

| **Acronym** | **Definition** |
| --- | --- |
| BIO-1 | Annual mean temperature |
| BIO-2 | Mean diurnal temperature |
| BIO-3 | Isothermality |
| BIO-4 | Temperature seasonality |
| BIO-5 | Maximum temperature (TMAX) of the warmest month |
| BIO-6 | Minimum temperature (TMIN) of the coldest month |
| BIO-7 | Temperature annual range |
| BIO-8 | Mean temperature (TMEAN) of the wettest quarter |
| BIO-9 | Mean temperature (TMEAN) of the wettest quarter |
| BIO-10 | Mean temperature (TMEAN) of the wettest quarter |
| BIO-11 | Mean temperature (TMEAN) of the wettest quarter |
| BIO-12 | Annual precipitation amounts |
| BIO-13 | Precipitation of the wettest month |
| BIO-14 | Precipitation of the driest month |
| BIO-15 | Precipitation seasonality |
| BIO-16 | Precipitation of the wettest quarter |
| BIO-17 | Precipitation of the driest quarter |
| BIO-18 | Precipitation of the warmest quarter |
| BIO-19 | Precipitation of the coldest quarter |

S1.2 Extreme variables

| **Acronym** | **Definition** |
| --- | --- |
| EXV-1 | Number of days with TMIN < -10°C |
| EXV-2 | Number of days with TMIN > 20°C |
| EXV-3 | Longest period with TMIN < -10°C |
| EXV-4 | Longest period with TMIN > 20°C |
| EXV-5 | Number of days with TMEAN < 0°C |
| EXV-6 | Number of days with TMEAN > 25°C |
| EXV-7 | Longest period with TMEAN < 0°C |
| EXV-8 | Longest period with TMEAN < 25°C |
| EXV-9 | Number of days with TMAX < 0°C |
| EXV-10 | Number of days with TMIAX > 37°C |
| EXV-11 | Longest period with TMAX < 0°C |
| EXV-12 | Longest period with TMAX < 37°C |
| EXV-13 | Number of days with PRE < 1mm |
| EXV-14 | Number of days with PRE > 20mm |
| EXV-15 | Longest period with PRE > 20mm |
| EXV-16 | Longest dry period |
| EXV-17 | Longest wet period |
| EXV-18 | Precipitation intensity |

S1.3 Land-use variables

| **Acronym** | **Definition** |
| --- | --- |
| TBET | Temperate broadleaf evergreen trees |
| TDT | Temperate deciduous trees |
| ECT | Evergreen coniferous trees |
| DCT | Deciduous coniferous trees |
| CS | Coniferous shrubs |
| DS | Deciduous shrubs |
| C3 | C3 grass |
| C4 | C4 grass |
| T | Tundra |
| S | Swamp |
| NIC | Non-irrigated crops |
| IC | Irrigated crops |
| U | Urban |
| B | Bare |

**S2 Model evaluation**

S2.1 Predictor selection

S2.1.1 Bioclimatic variables

*Table S2.1.1: Incorporated* ***bioclimatic*** *predictors (x) for each species and model setup (buffered, sampled, selection). The predictor with the highest contribution is highlighted in red, the number represents the overall rank. Predictors representing the highest interaction are highlighted in green, predictors involved in both (contribution and interaction) are highlighted in purple.*

| **Species** | **Buffered** | **Sampled** | **Selection** | **BIO1** | **BIO2** | **BIO3** | **BIO4** | **BIO5** | **BIO6** | **BIO7** | **BIO8** | **BIO9** | **BIO10** | **BIO11** | **BIO12** | **BIO13** | **BIO14** | **BIO15** | **BIO16** | **BIO17** | **BIO18** | **BIO19** |
| --- | --- | --- | --- | --- | --- | --- | --- | --- | --- | --- | --- | --- | --- | --- | --- | --- | --- | --- | --- | --- | --- | --- |
| **atroparvus** |  |  | **I** | **x** | **I** | **x** | **x** | **x** | **x** | **x** | **x** | **I** | **x** | **x** | **x** | **x** | **x** | **x** | **x** | **x** | **C1** | **x** |
|  |  |  | **II** | **x** | **x** | **x** | **x** | **x** | **x** | **x** | **x** | **x** | **x** | **x** | **x** | **x** | **x** | **x** | **x** | **I** | **C1** | **x** |
|  |  |  | **III** | **x** | **x** | **x** | **x** | **x** | **x** | **I** | **x** | **x** | **x** | **x** | **x** | **x** | **x** | **x** | **x** | **x** | **C1** | **x** |
|  |  |  | **IV** |  | **x** | **x** | **x** | **x** |  | **x** | **x** |  |  | **x** |  | **x** |  | **x** |  |  | **C1** | **x** |
|  | **x** |  | **I** | **x** | **x** | **x** | **x** | **x** | **x** | **x** | **x** | **x** | **x** | **x** | **x** | **x** | **x** | **x** | **x** | **x** | **C1** | **x** |
|  | **x** |  | **II** | **x** | **x** | **x** | **x** | **x** | **x** | **I** | **x** | **x** | **x** | **x** | **x** | **x** | **x** | **x** | **x** | **x** | **C1** | **x** |
|  | **x** |  | **III** |  | **x** | **x** | **x** | **x** | **x** | **x** | **x** | **x** | **x** |  | **I** | **x** | **x** | **x** | **x** | **x** | **C1** | **I** |
|  | **x** |  | **IV** |  | **x** | **x** | **x** | **x** | **x** | **x** | **I** | **x** |  |  |  | **x** |  | **x** |  |  | **C1** | **I** |
|  |  | **x** | **I** | **x** | **x** | **x** | **x** | **x** | **x** | **x** | **I** | **x** | **x** | **x** | **x** | **x** | **x** | **x** | **x** | **I** | **C2** | **x** |
|  |  | **x** | **II** | **x** | **x** | **I** | **x** | **x** | **x** | **x** | **x** | **x** | **x** | **x** | **x** | **x** | **x** | **x** | **x** | **x** | **C2** | **x** |
|  |  | **x** | **III** |  | **x** | **x** | **I** | **x** | **x** | **x** | **x** | **x** | **I** | **x** | **x** | **x** | **x** | **x** | **x** | **x** | **C2** | **x** |
|  |  | **x** | **IV** |  | **x** | **x** | **x** | **x** |  | **x** | **x** | **x** |  | **x** |  |  |  | **x** |  |  | **C1** | **x** |
|  | **x** | **x** | **I** | **x** | **x** | **I** | **x** | **x** | **x** | **x** | **I** | **x** | **x** | **x** | **x** | **x** | **x** | **x** | **x** | **x** | **C1** | **x** |
|  | **x** | **x** | **II** |  | **I** | **x** | **x** | **x** | **x** | **x** | **x** | **I** | **x** | **x** | **x** | **x** | **x** | **x** |  | **x** | **C1** | **x** |
|  | **x** | **x** | **III** |  | **x** | **x** | **x** | **x** | **x** | **I** | **x** | **x** |  | **x** | **x** | **x** | **x** | **x** |  | **x** | **C1** | **x** |
|  | **x** | **x** | **IV** |  | **x** | **x** | **x** | **x** | **I** | **x** | **x** |  |  |  |  |  |  | **x** |  |  | **C1** | **x** |
| **labranchiae** |  |  | **I** | **x** | **x** | **x** | **IC2** | **x** | **x** | **x** | **x** | **x** | **x** | **x** | **x** | **x** | **x** | **x** | **x** | **x** | **x** | **x** |
|  |  |  | **II** | **x** | **x** | **x** | **IC2** | **x** | **x** | **x** | **x** | **x** | **x** | **x** | **x** | **x** | **x** | **x** | **x** | **x** | **x** | **x** |
|  |  |  | **III** |  |  | **x** | **C2** | **x** | **x** | **x** | **x** |  |  |  | **x** |  | **x** | **x** |  | **x** | **I** | **x** |
|  |  |  | **IV** |  |  | **x** | **IC2** |  | **x** | **x** | **x** |  |  |  |  |  |  | **x** |  |  |  | **x** |
|  | **x** |  | **I** | **x** | **x** | **x** | **C2** | **x** | **x** | **x** | **I** | **x** | **x** | **x** | **x** | **x** | **x** | **x** | **x** | **x** | **x** | **x** |
|  | **x** |  | **II** | **x** | **x** | **x** | **C2** | **x** | **x** | **x** | **x** | **x** | **I** | **x** | **x** | **x** | **x** | **x** | **x** | **x** | **x** | **x** |
|  | **x** |  | **III** | **x** | **x** | **x** | **C2** | **x** |  | **x** | **x** | **x** | **I** | **x** | **x** | **x** | **x** | **x** | **x** | **x** |  | **x** |
|  | **x** |  | **IV** |  | **x** | **x** | **IC2** |  |  | **x** | **x** |  |  | **x** |  |  |  | **x** | **x** |  |  | **x** |
|  |  | **x** | **I** | **x** | **x** | **x** | **IC2** | **x** | **x** | **x** | **x** | **x** | **x** | **x** | **x** | **x** | **x** | **x** | **x** | **x** | **x** | **x** |
|  |  | **x** | **II** | **x** | **x** | **x** | **IC2** | **x** | **x** | **x** | **x** | **x** | **x** | **x** | **x** | **x** | **x** | **x** | **x** | **x** | **x** | **x** |
|  |  | **x** | **III** |  | **x** | **x** | **C2** | **x** | **x** | **x** | **x** | **x** |  | **x** | **x** |  | **x** | **I** | **x** | **x** | **x** | **x** |
|  |  | **x** | **IV** |  | **x** | **x** | **IC2** |  |  | **x** | **x** |  |  | **x** |  |  |  | **x** |  |  |  | **x** |
|  | **x** | **x** | **I** | **x** | **x** | **x** | **IC2** | **x** | **x** | **x** | **x** | **x** | **x** | **x** | **x** | **x** | **I** | **x** | **x** | **x** | **x** | **x** |
|  | **x** | **x** | **II** | **x** | **x** | **x** | **C2** | **x** | **x** | **x** | **x** | **x** | **x** | **x** | **x** | **x** | **x** | **I** | **x** | **x** | **x** | **x** |
|  | **x** | **x** | **III** |  | **x** | **x** | **C2** | **x** | **x** | **x** | **I** | **x** | **x** | **x** | **x** | **x** | **x** | **x** |  | **x** | **x** | **x** |
|  | **x** | **x** | **IV** |  | **x** | **x** | **I** |  | **x** | **x** | **x** | **x** |  |  |  | **x** |  | **C2** |  |  |  | **x** |
| **messeae** |  |  | **I** | **x** | **I** | **x** | **x** | **x** | **x** | **x** | **x** | **C1** | **x** | **x** | **x** | **x** | **x** | **x** | **x** | **x** | **x** | **x** |
|  |  |  | **II** | **x** | **x** | **x** | **x** | **x** | **x** | **x** | **x** | **C1** | **x** | **x** | **x** | **x** | **x** | **x** | **x** | **x** | **I** | **x** |
|  |  |  | **III** | **x** | **x** | **x** | **x** | **x** | **x** | **x** | **x** | **C1** | **I** |  | **x** | **x** | **x** | **x** | **x** | **x** | **x** | **x** |
|  |  |  | **IV** |  | **I** | **x** | **x** |  |  | **x** | **x** | **IC1** |  |  |  |  | **x** | **x** |  |  |  | **x** |
|  | **x** |  | **I** | **x** | **I** | **x** | **x** | **x** | **x** | **x** | **x** | **IC1** | **x** | **x** | **x** | **x** | **x** | **x** | **x** | **x** | **x** | **x** |
|  | **x** |  | **II** | **x** | **x** | **x** | **x** | **x** | **x** | **x** | **x** | **C1** | **x** | **x** | **x** | **x** | **x** | **x** | **x** | **x** | **I** | **x** |
|  | **x** |  | **III** | **x** | **I** | **x** | **x** | **x** | **x** | **x** | **x** | **IC1** | **x** |  | **x** | **x** | **x** | **x** | **x** | **x** | **x** | **x** |
|  | **x** |  | **IV** |  | **x** | **x** | **I** |  |  | **x** | **x** | **C1** |  |  |  |  | **x** | **x** |  |  |  | **x** |
|  |  | **x** | **I** | **x** | **x** | **x** | **x** | **x** | **x** | **x** | **I** | **C1** | **x** | **x** | **x** | **x** | **x** | **x** | **x** | **I** | **x** | **x** |
|  |  | **x** | **II** | **x** | **x** | **x** | **x** | **x** | **x** | **x** | **I** | **C1** | **x** | **x** | **x** | **x** | **x** | **x** | **x** | **x** | **x** | **x** |
|  |  | **x** | **III** | **x** | **x** | **x** | **x** | **x** |  | **x** | **x** | **IC1** | **x** | **x** |  |  | **x** | **x** |  | **x** | **x** | **x** |
|  |  | **x** | **IV** |  | **x** | **x** | **x** | **x** |  | **x** | **x** | **C1** |  |  |  |  | **x** | **x** |  |  | **I** | **x** |
|  | **x** | **x** | **I** | **x** | **x** | **x** | **I** | **x** | **x** | **x** | **x** | **C1** | **x** | **x** | **x** | **x** | **I** | **x** | **x** | **x** | **x** | **x** |
|  | **x** | **x** | **II** | **x** | **x** | **x** | **x** | **x** | **x** | **x** | **I** | **C1** | **x** | **x** | **x** | **x** | **x** | **x** | **x** | **I** | **x** | **x** |
|  | **x** | **x** | **III** | **x** | **x** | **x** | **x** | **x** |  | **x** | **I** | **C1** | **x** |  | **x** |  | **x** | **x** |  | **x** | **I** | **x** |
|  | **x** | **x** | **IV** |  | **x** | **x** | **x** |  |  | **x** | **I** | **C1** |  |  |  |  |  | **x** |  |  | **x** | **I** |
| **sacharovi** |  |  | **I** | **x** | **x** | **x** | **x** | **x** | **x** | **I** | **C1** | **x** | **x** | **x** | **x** | **x** | **x** | **x** | **x** | **x** | **x** | **x** |
|  |  |  | **II** | **x** | **x** | **x** | **x** | **x** | **x** | **x** | **C1** | **x** | **x** | **I** | **x** | **x** | **x** | **x** | **x** | **x** | **x** | **x** |
|  |  |  | **III** | **x** | **x** | **I** | **x** | **x** | **x** | **x** | **C1** | **x** | **x** | **x** | **x** |  | **x** | **x** | **x** | **x** | **x** | **x** |
|  |  |  | **IV** |  | **x** | **x** | **x** |  |  | **I** | **C1** |  |  |  |  |  | **x** | **x** | **x** |  |  | **x** |
|  | **x** |  | **I** | **x** | **x** | **x** | **x** | **x** | **x** | **I** | **C1** | **x** | **x** | **x** | **x** | **x** | **x** | **x** | **x** | **x** | **x** | **x** |
|  | **x** |  | **II** | **x** | **x** | **x** | **x** | **x** | **x** | **x** | **C1** | **x** | **I** | **x** | **x** | **x** | **x** | **x** | **x** | **x** | **x** | **x** |
|  | **x** |  | **III** | **x** | **x** | **x** | **x** | **x** |  | **x** | **IC1** |  | **x** | **x** | **x** |  | **x** | **x** |  | **x** | **x** | **x** |
|  | **x** |  | **IV** |  | **x** | **I** | **x** |  |  | **x** | **IC1** |  |  |  |  |  |  | **x** |  |  | **x** | **x** |
|  |  | **x** | **I** | **x** | **I** | **x** | **x** | **x** | **x** | **x** | **C1** | **x** | **x** | **x** | **x** | **x** | **x** | **x** | **x** | **x** | **x** | **x** |
|  |  | **x** | **II** | **x** | **x** | **x** | **x** | **x** | **x** | **I** | **C1** | **x** | **x** | **x** | **x** | **x** | **x** | **x** | **x** | **x** | **x** | **x** |
|  |  | **x** | **III** |  | **x** | **x** | **x** | **x** | **I** | **x** | **C1** | **x** | **x** |  | **x** | **x** | **x** | **x** | **x** | **x** | **x** | **x** |
|  |  | **x** | **IV** |  | **x** | **I** | **x** |  |  | **x** | **IC1** |  |  |  |  |  | **x** | **x** | **x** |  |  | **x** |
|  | **x** | **x** | **I** | **x** | **x** | **x** | **x** | **x** | **x** | **x** | **C1** | **x** | **x** | **I** | **x** | **x** | **x** | **x** | **x** | **x** | **x** | **x** |
|  | **x** | **x** | **II** | **x** | **x** | **x** | **x** | **x** | **x** | **x** | **IC1** | **x** | **x** | **x** | **x** | **x** | **x** | **x** | **x** | **x** | **x** | **x** |
|  | **x** | **x** | **III** | **x** | **x** | **x** | **x** | **x** | **x** | **x** | **C1** | **x** |  | **x** | **x** | **x** | **x** | **x** |  | **x** | **x** | **x** |
|  | **x** | **x** | **IV** |  | **x** | **x** | **I** |  |  | **x** | **C1** | **x** |  |  |  |  | **x** | **x** |  |  |  | **x** |
| **sergentii** |  |  | **I** | **x** | **x** | **x** | **x** | **C1** | **x** | **x** | **x** | **I** | **x** | **x** | **x** | **x** | **x** | **I** | **x** | **x** | **x** | **x** |
|  |  |  | **II** | **x** | **I** | **x** | **x** | **C1** | **x** | **x** | **x** | **x** | **x** | **x** | **x** | **x** | **x** | **I** | **x** | **x** | **x** | **x** |
|  |  |  | **III** | **x** | **x** | **x** | **x** | **C1** | **x** | **x** | **x** |  |  | **x** | **x** | **x** |  | **x** | **x** |  | **x** | **x** |
|  |  |  | **IV** |  | **x** | **x** | **x** | **C1** | **I** | **x** | **I** |  |  |  | **x** |  |  | **x** |  |  |  |  |
|  | **x** |  | **I** | **x** | **x** | **x** | **x** | **x** | **x** | **x** | **x** | **x** | **x** | **x** | **C1** | **x** | **x** | **x** | **x** | **x** | **x** | **x** |
|  | **x** |  | **II** | **x** | **x** | **x** | **x** | **C1** | **I** | **x** | **x** | **x** | **x** | **x** | **x** | **x** | **x** | **I** | **x** | **x** | **x** | **x** |
|  | **x** |  | **III** | **x** | **x** | **x** | **x** | **C1** | **x** | **x** | **x** |  |  | **x** | **x** |  |  | **x** | **x** | **x** | **x** | **x** |
|  | **x** |  | **IV** |  | **x** | **x** | **I** | **x** | **x** | **x** | **x** |  |  |  | **IC1** |  |  | **x** |  |  |  |  |
|  |  | **x** | **I** | **x** | **x** | **x** | **x** | **x** | **x** | **x** | **x** | **x** | **x** | **x** | **C1** | **x** | **x** | **x** | **x** | **x** | **x** | **x** |
|  |  | **x** | **II** | **x** | **x** | **x** | **x** | **C1** | **x** | **x** | **x** | **x** | **x** | **x** | **x** | **x** | **x** | **x** | **x** | **x** | **x** | **x** |
|  |  | **x** | **III** | **x** | **x** | **x** | **x** | **x** | **x** | **x** | **x** | **x** | **x** | **x** | **C1** | **x** | **x** | **x** | **x** |  | **x** | **x** |
|  |  | **x** | **IV** |  | **x** | **x** | **x** | **x** | **x** | **x** | **x** |  |  |  | **IC1** |  |  | **x** |  |  |  |  |
|  | **x** | **x** | **I** | **x** | **x** | **x** | **x** | **x** | **x** | **x** | **x** | **x** | **x** | **x** | **C1** | **x** | **x** | **x** | **x** | **x** | **x** | **x** |
|  | **x** | **x** | **II** | **x** | **x** | **x** | **x** | **C1** | **x** | **x** | **x** | **x** | **x** | **x** | **x** | **x** | **x** | **x** | **x** | **x** | **x** | **x** |
|  | **x** | **x** | **III** | **x** | **x** | **x** | **x** | **x** | **x** | **x** | **x** | **x** | **x** | **x** | **C1** | **x** | **x** | **x** | **x** |  | **x** | **x** |
|  | **x** | **x** | **IV** |  | **x** | **x** | **x** | **x** | **x** | **x** | **x** |  |  |  | **IC1** |  |  | **x** |  |  |  |  |
| **superpictus** |  |  | **I** | **x** | **x** | **x** | **x** | **x** | **x** | **I** | **C1** | **x** | **x** | **x** | **x** | **x** | **x** | **x** | **x** | **x** | **x** | **x** |
|  |  |  | **II** | **x** | **x** | **x** | **I** | **x** | **x** | **x** | **IC1** | **x** | **x** | **x** | **x** | **x** | **x** | **x** | **x** | **x** | **x** | **x** |
|  |  |  | **III** |  | **x** | **x** | **x** | **x** | **x** |  | **C1** | **x** |  | **x** |  | **x** | **x** | **x** | **x** | **x** | **x** | **x** |
|  |  |  | **IV** |  | **x** | **x** | **x** |  |  |  | **C1** | **x** |  |  |  |  |  | **x** |  |  |  | **x** |
|  | **x** |  | **I** | **x** | **x** | **x** | **x** | **x** | **x** | **I** | **C1** | **x** | **x** | **x** | **x** | **x** | **x** | **x** | **x** | **x** | **x** | **x** |
|  | **x** |  | **II** | **x** | **x** | **x** | **I** | **x** | **x** | **x** | **IC1** | **x** | **x** | **x** | **x** | **x** | **x** | **x** | **x** | **x** | **x** | **x** |
|  | **x** |  | **III** |  | **x** | **x** | **x** | **x** | **x** |  | **C1** | **x** |  | **x** |  | **x** | **x** | **x** | **x** | **x** | **x** | **x** |
|  | **x** |  | **IV** |  | **x** | **x** | **x** |  |  |  | **C1** | **x** |  |  |  |  |  | **x** |  |  |  | **x** |
|  |  | **x** | **I** | **x** | **x** | **x** | **x** | **x** | **x** | **x** | **C1** | **x** | **x** | **x** | **x** | **x** | **x** | **x** | **x** | **x** | **x** | **x** |
|  |  | **x** | **II** | **x** | **x** | **x** | **x** | **x** | **x** | **I** | **C1** | **x** | **x** | **x** | **x** | **x** | **x** | **x** | **x** | **x** | **x** | **x** |
|  |  | **x** | **III** |  | **x** | **x** | **x** | **x** | **x** | **x** | **C1** | **I** |  | **x** | **x** |  | **x** | **x** | **x** | **x** | **x** | **x** |
|  |  | **x** | **IV** |  | **x** | **x** | **x** |  |  | **x** | **C1** | **I** |  |  |  |  |  | **x** | **x** |  |  | **x** |
|  | **x** | **x** | **I** | **x** | **x** | **x** | **x** | **x** | **x** | **x** | **C1** | **x** | **x** | **x** | **x** | **x** | **x** | **x** | **x** | **x** | **x** | **x** |
|  | **x** | **x** | **II** |  | **x** | **x** | **x** | **x** | **x** | **x** | **IC1** | **x** | **x** |  | **x** | **x** | **x** | **x** | **x** | **x** | **x** | **I** |
|  | **x** | **x** | **III** |  | **x** | **x** | **I** | **x** | **x** |  | **IC1** | **x** |  |  |  | **x** | **x** | **x** | **x** | **x** | **x** | **x** |
|  | **x** | **x** | **IV** |  | **x** | **x** | **x** |  |  |  | **C1** | **x** |  |  |  |  | **x** | **x** | **x** |  |  | **I** |

S2.1.2 Extreme variables and absolute humidity

*Table S2.1.2: Incorporated* ***extreme*** *predictors (x) for each species and model setup (buffered, sampled, selection). The predictor with the highest contribution is highlighted in red, the number represents the overall rank. Predictors representing the highest interaction are highlighted in green, predictors involved in both (contribution and interaction) are highlighted in purple.*

| **Species** | **Buffered** | **Sampled** | **Selection** | **EXV1** | **EXV2** | **EXV3** | **EXV4** | **EXV5** | **EXV6** | **EXV7** | **EXV8** | **EXV9** | **EXV10** | **EXV11** | **EXV12** | **EXV13** | **EXV14** | **EXV15** | **EXV16** | **EXV17** | **EXV18** | **AHUM** |
| --- | --- | --- | --- | --- | --- | --- | --- | --- | --- | --- | --- | --- | --- | --- | --- | --- | --- | --- | --- | --- | --- | --- |
| **xtroparvus** |  |  | **I** | **x** | **x** | **C8** | **x** | **x** | **x** | **x** | **x** | **x** | **x** | **x** | **x** | **x** | **x** | **x** | **x** | **x** | **x** | **x** |
|  |  |  | **II** | **x** | **x** | **C12** | **x** | **x** | **x** | **x** | **x** | **x** | **x** | **x** | **x** | **x** | **x** | **x** | **x** | **x** | **x** | **x** |
|  |  |  | **III** |  |  | **C4** |  |  |  | **x** |  |  |  |  |  | **x** | **x** | **x** | **x** | **x** | **x** | **x** |
|  |  |  | **IV** |  |  | **C4** |  |  |  |  |  |  |  |  |  |  | **x** |  |  |  |  | **I** |
|  | **x** |  | **I** | **x** | **x** | **C2** | **x** | **I** | **x** | **x** | **x** | **x** | **x** | **I** | **x** | **x** | **x** | **x** | **x** | **x** | **x** | **x** |
|  | **x** |  | **II** | **x** | **x** | **C3** | **x** | **x** | **x** | **x** | **x** | **x** |  | **x** | **x** | **x** | **x** | **x** | **x** | **x** | **x** | **I** |
|  | **x** |  | **III** |  | **x** | **C4** |  |  |  |  |  |  |  |  |  | **x** |  |  |  | **x** | **x** | **x** |
|  | **x** |  | **IV** |  |  | **C3** |  |  |  |  |  |  |  |  |  |  |  |  |  |  | **x** | **x** |
|  |  | **x** | **I** | **x** | **x** | **x** | **x** | **x** | **x** | **C7** | **x** | **x** | **x** | **x** | **x** | **x** | **x** | **x** | **x** | **x** | **x** | **x** |
|  |  | **x** | **II** | **x** | **x** | **C15** | **x** |  | **x** | **x** | **x** | **x** | **x** | **x** |  | **x** | **x** | **x** | **x** | **x** | **x** | **x** |
|  |  | **x** | **III** | **x** |  | **C12** |  |  | **x** | **x** |  |  |  | **x** |  | **x** | **x** | **x** |  | **x** | **x** | **x** |
|  |  | **x** | **IV** |  |  | **C10** |  |  |  |  |  |  |  |  |  |  |  |  |  |  | **x** | **I** |
|  | **x** | **x** | **I** | **x** | **x** | **C3** | **x** | **x** | **x** | **x** | **x** | **x** | **x** | **x** | **x** | **x** | **x** | **x** | **x** | **x** | **x** | **x** |
|  | **x** | **x** | **II** | **x** |  | **C3** | **x** |  |  |  | **x** | **x** |  | **x** |  | **x** | **x** | **x** | **x** | **x** | **x** | **x** |
|  | **x** | **x** | **III** |  |  | **C3** |  |  |  |  |  |  |  |  |  | **x** | **x** | **x** |  |  | **x** | **x** |
|  | **x** | **x** | **IV** |  |  | **C3** |  |  |  |  |  |  |  |  |  |  |  |  |  |  | **x** | **I** |
| **labranchiae** |  |  | **I** | **x** | **x** | **x** | **x** | **x** | **x** | **x** | **x** | **x** | **x** | **x** | **x** | **x** | **x** | **C1** | **x** | **x** | **x** | **I** |
|  |  |  | **II** |  |  |  |  |  |  |  | **x** |  | **x** |  |  | **x** | **x** | **C1** | **x** | **x** | **x** | **I** |
|  |  |  | **III** |  |  |  |  |  |  |  |  |  |  |  |  | **x** | **x** | **C1** | **x** | **I** | **x** | **x** |
|  |  |  | **IV** |  |  |  |  |  |  |  |  |  |  |  |  |  |  | **C1** |  | **x** |  | **I** |
|  | **x** |  | **I** | **x** | **x** | **x** | **x** | **x** | **x** | **x** | **x** | **x** | **x** | **x** | **x** | **x** | **x** | **C1** | **x** | **x** | **x** | **I** |
|  | **x** |  | **II** | **x** | **x** | **x** | **x** | **x** | **x** | **x** | **x** | **x** | **x** | **x** | **x** | **I** | **x** | **C1** | **x** | **x** | **x** | **x** |
|  | **x** |  | **III** |  |  |  |  |  |  |  |  |  |  |  |  | **I** | **x** | **C1** | **x** | **x** | **x** | **x** |
|  | **x** |  | **IV** |  |  |  |  |  |  |  |  |  |  |  |  |  |  | **C1** |  | **x** |  | **I** |
|  |  | **x** | **I** | **x** | **x** | **x** | **x** | **x** | **x** | **x** | **x** | **x** | **x** | **x** | **x** | **x** | **x** | **C1** | **x** | **x** | **x** | **I** |
|  |  | **x** | **II** |  | **x** |  | **x** | **x** | **x** |  | **x** | **x** | **x** |  | **x** | **x** | **x** | **C1** | **x** | **x** | **x** | **I** |
|  |  | **x** | **III** |  |  |  |  |  |  |  |  |  |  |  |  | **x** | **x** | **C1** | **x** | **x** | **x** | **I** |
|  |  | **x** | **IV** |  |  |  |  |  |  |  |  |  |  |  |  |  |  | **C1** |  | **x** |  | **I** |
|  | **x** | **x** | **I** | **x** | **x** | **x** | **x** | **x** | **x** | **x** | **x** | **x** | **x** | **x** | **x** | **x** | **x** | **C1** | **x** | **x** | **x** | **x** |
|  | **x** | **x** | **II** | **x** | **x** | **x** | **x** | **x** | **x** | **x** | **x** | **x** | **x** | **x** | **x** | **x** | **x** | **C1** | **x** | **x** | **x** | **I** |
|  | **x** | **x** | **III** |  |  |  |  |  |  |  |  |  |  |  |  | **x** | **x** | **C1** | **x** | **x** | **x** | **I** |
|  | **x** | **x** | **IV** |  |  |  |  |  |  |  |  |  |  |  |  |  |  | **C1** |  | **x** |  | **I** |
| **messeae** |  |  | **I** | **x** | **x** | **x** | **x** | **x** | **x** | **x** | **x** | **x** | **x** | **x** | **x** | **x** | **x** | **x** | **x** | **x** | **IC5** | **x** |
|  |  |  | **II** | **x** | **x** | **x** | **x** | **x** | **x** | **x** | **x** | **x** | **x** | **x** | **x** | **I** | **x** | **x** | **x** | **x** | **C6** | **x** |
|  |  |  | **III** | **x** |  |  |  |  |  | **x** |  |  |  |  |  | **x** | **x** | **x** |  | **x** | **C6** | **x** |
|  |  |  | **IV** | **x** |  |  |  |  |  |  |  |  |  |  |  |  |  |  |  |  | **C5** | **x** |
|  | **x** |  | **I** | **x** | **x** | **x** | **x** | **x** | **x** | **x** | **x** | **x** | **x** | **x** | **x** | **x** | **x** | **x** | **x** | **x** | **C5** | **x** |
|  | **x** |  | **II** | **x** | **x** |  | **x** | **x** | **x** | **x** | **x** | **x** |  | **x** |  | **x** | **x** | **x** | **I** | **x** | **C8** | **x** |
|  | **x** |  | **III** |  |  |  |  |  |  | **x** |  |  |  |  |  | **x** |  | **x** | **x** | **x** | **C6** | **x** |
|  | **x** |  | **IV** |  |  |  |  |  |  |  |  |  |  |  |  |  |  | **x** |  | **I** | **C6** | **x** |
|  |  | **x** | **I** | **x** | **x** | **x** | **x** | **x** | **x** | **x** | **x** | **x** | **x** | **x** | **x** | **x** | **x** | **x** | **x** | **x** | **C6** | **x** |
|  |  | **x** | **II** | **x** |  |  | **x** |  |  | **x** | **x** | **x** |  | **x** |  | **x** | **x** | **x** | **x** | **x** | **C6** | **x** |
|  |  | **x** | **III** |  |  |  |  |  |  |  |  | **x** |  |  |  | **x** | **x** |  |  | **x** | **C5** | **x** |
|  |  | **x** | **IV** |  |  |  |  |  |  |  |  |  |  |  |  |  |  |  |  |  | **C5** | **x** |
|  | **x** | **x** | **I** | **x** | **x** | **x** | **x** | **x** | **x** | **x** | **x** | **x** | **x** | **x** | **x** | **x** | **x** | **x** | **x** | **x** | **C5** | **x** |
|  | **x** | **x** | **II** |  | **x** |  |  | **x** | **x** | **x** |  | **x** |  | **x** |  | **x** | **x** | **x** | **x** | **x** | **C6** | **x** |
|  | **x** | **x** | **III** |  |  |  |  |  |  |  |  |  |  |  |  | **x** |  | **x** | **x** | **x** | **C6** | **x** |
|  | **x** | **x** | **IV** |  |  |  |  |  |  |  |  |  |  |  |  |  |  | **x** |  | **x** | **C5** | **x** |
| **sacharovi** |  |  | **I** | **x** | **x** | **x** | **x** | **x** | **x** | **x** | **x** | **x** | **x** | **x** | **x** | **x** | **x** | **x** | **C8** | **x** | **x** | **I** |
|  |  |  | **II** | **x** | **x** | **I** | **x** | **x** | **x** | **x** | **x** | **x** | **x** | **x** | **x** | **x** | **x** | **x** | **C9** | **x** | **x** | **x** |
|  |  |  | **III** | **C9** |  | **x** | **x** | **x** |  | **x** |  |  |  |  |  | **x** | **x** |  | **x** | **x** | **x** | **x** |
|  |  |  | **IV** | **x** |  |  | **x** | **C9** |  |  |  |  |  |  |  |  |  |  |  |  | **x** | **I** |
|  | **x** |  | **I** | **C10** | **x** | **x** | **x** | **x** | **x** | **x** | **x** | **x** | **x** | **x** | **x** | **x** | **x** | **x** | **x** | **x** | **x** | **I** |
|  | **x** |  | **II** | **x** | **x** | **x** | **x** | **x** | **x** | **x** | **x** | **x** | **x** | **x** |  | **I** | **x** | **x** | **C10** | **x** | **x** | **x** |
|  | **x** |  | **III** | **x** |  | **x** |  | **C8** |  | **x** |  |  |  | **x** |  | **I** | **x** | **x** | **x** |  | **x** | **x** |
|  | **x** |  | **IV** | **x** |  |  |  | **C9** |  |  |  |  |  |  |  |  |  |  |  |  | **x** | **x** |
|  |  | **x** | **I** | **x** | **x** | **x** | **x** | **x** | **x** | **x** | **I** | **x** | **x** | **x** | **x** | **x** | **x** | **x** | **C7** | **x** | **x** | **x** |
|  |  | **x** | **II** | **C8** | **x** |  | **x** | **x** | **x** | **x** | **x** | **x** |  | **x** |  | **x** | **x** | **x** | **x** | **x** | **x** | **I** |
|  |  | **x** | **III** | **x** |  |  |  | **x** |  | **x** |  | **C11** |  | **x** |  | **x** |  | **x** | **x** | **x** | **x** | **x** |
|  |  | **x** | **IV** |  |  |  |  |  |  |  |  | **C9** |  |  |  |  |  |  |  |  | **x** | **x** |
|  | **x** | **x** | **I** | **x** | **I** | **x** | **x** | **C8** | **x** | **x** | **x** | **x** | **x** | **x** | **x** | **x** | **x** | **x** | **x** | **x** | **x** | **x** |
|  | **x** | **x** | **II** | **C9** | **x** | **x** | **x** | **x** | **x** | **I** | **x** | **x** | **x** | **x** | **x** | **x** | **x** | **x** | **x** | **x** | **x** | **x** |
|  | **x** | **x** | **III** | **x** | **x** | **x** | **x** | **x** |  | **x** |  | **I** |  | **x** |  | **x** |  | **x** | **C9** | **x** | **I** | **x** |
|  | **x** | **x** | **IV** | **x** |  |  |  | **C9** |  |  |  |  |  |  |  |  |  |  |  |  | **x** | **x** |
| **sergentii** |  |  | **I** | **x** | **x** | **x** | **x** | **x** | **x** | **x** | **x** | **x** | **C9** | **x** | **x** | **x** | **x** | **x** | **x** | **x** | **x** | **x** |
|  |  |  | **II** |  | **x** |  | **x** | **x** | **x** |  | **x** |  | **C10** |  | **x** | **x** |  |  | **x** | **x** | **x** | **x** |
|  |  |  | **III** |  | **x** |  | **x** |  | **x** |  | **x** |  | **C10** |  |  |  |  |  | **x** | **x** | **x** | **x** |
|  |  |  | **IV** |  |  |  |  |  |  |  |  |  |  |  |  |  |  |  |  |  | **C11** | **x** |
|  | **x** |  | **I** | **x** | **x** | **x** | **x** | **x** | **x** | **x** | **x** | **x** | **C12** | **x** | **x** | **x** | **x** | **x** | **x** | **x** | **x** | **I** |
|  | **x** |  | **II** |  | **x** |  | **x** | **x** | **x** |  | **x** |  | **C9** |  | **x** | **x** | **x** |  | **x** | **x** | **x** | **x** |
|  | **x** |  | **III** |  | **x** |  | **x** |  | **x** |  | **x** |  | **C8** |  | **x** | **x** |  |  | **x** | **x** | **x** | **x** |
|  | **x** |  | **IV** |  |  |  |  |  |  |  |  |  |  |  |  |  |  |  |  |  | **C14** | **I** |
|  |  | **x** | **I** | **x** | **x** | **x** | **x** | **x** | **x** | **x** | **x** | **x** | **x** | **x** | **x** | **x** | **x** | **x** | **C10** | **x** | **x** | **x** |
|  |  | **x** | **II** | **x** | **x** |  | **x** | **x** | **x** | **x** | **x** | **x** | **x** |  | **x** | **C14** | **x** | **x** | **x** | **x** | **x** | **x** |
|  |  | **x** | **III** |  | **x** |  | **x** |  | **x** |  | **x** |  | **x** |  |  | **C12** |  |  | **x** | **x** | **x** | **I** |
|  |  | **x** | **IV** |  |  |  |  |  |  |  |  |  |  |  |  |  |  |  |  |  | **C16** | **x** |
|  | **x** | **x** | **I** | **x** | **x** | **x** | **x** | **x** | **x** | **x** | **x** | **x** | **x** | **x** | **x** | **x** | **x** | **x** | **C10** | **x** | **x** | **x** |
|  | **x** | **x** | **II** | **x** | **x** |  | **x** | **x** | **x** | **x** | **x** | **x** | **x** |  | **x** | **C14** | **x** | **x** | **x** | **x** | **x** | **x** |
|  | **x** | **x** | **III** |  | **x** |  | **x** |  | **x** |  | **x** |  | **x** |  |  | **C12** |  |  | **x** | **x** | **I** | **x** |
|  | **x** | **x** | **IV** |  |  |  |  |  |  |  |  |  |  |  |  |  |  |  |  |  | **C16** | **x** |
| **superpictus** |  |  | **I** | **x** | **x** | **x** | **x** | **x** | **x** | **x** | **x** | **x** | **x** | **x** | **x** | **x** | **x** | **x** | **x** | **x** | **C6** | **I** |
|  |  |  | **II** | **x** | **x** | **x** | **x** | **x** | **x** | **x** | **x** | **x** | **x** | **x** | **x** | **x** | **x** | **x** | **x** | **x** | **C5** | **x** |
|  |  |  | **III** | **x** |  |  |  | **x** |  | **x** | **I** | **x** |  |  |  | **x** | **x** | **x** | **x** | **x** | **C6** | **x** |
|  |  |  | **IV** |  |  |  |  | **C8** |  |  |  |  |  |  |  |  |  |  |  |  |  | **x** |
|  | **x** |  | **I** | **x** | **x** | **x** | **x** | **x** | **x** | **x** | **x** | **x** | **x** | **x** | **x** | **x** | **x** | **x** | **x** | **x** | **C6** | **I** |
|  | **x** |  | **II** | **x** | **x** | **x** | **x** | **x** | **x** | **x** | **x** | **x** | **x** | **x** | **x** | **x** | **x** | **x** | **x** | **x** | **C5** | **x** |
|  | **x** |  | **III** | **x** |  |  |  | **x** |  | **x** | **I** | **x** |  |  |  | **x** | **x** | **x** | **x** | **x** | **C6** | **x** |
|  | **x** |  | **IV** | **x** |  |  |  | **C8** |  |  |  |  |  |  |  |  |  |  |  |  |  | **x** |
|  |  | **x** | **I** | **x** | **I** | **x** | **x** | **x** | **x** | **x** | **x** | **x** | **x** | **x** | **x** | **x** | **x** | **x** | **x** | **x** | **C5** | **x** |
|  |  | **x** | **II** |  |  |  | **x** | **x** |  | **x** |  |  | **x** | **x** |  | **x** | **x** | **x** | **x** | **x** | **C5** | **x** |
|  |  | **x** | **III** |  |  |  |  | **IC5** |  | **x** |  |  |  |  |  | **x** | **x** | **x** |  | **x** | **x** | **I** |
|  |  | **x** | **IV** |  |  |  |  | **IC4** |  |  |  |  |  |  |  |  |  |  |  |  | **x** | **x** |
|  | **x** | **x** | **I** | **x** | **x** | **x** | **x** | **x** | **x** | **x** | **x** | **x** | **x** | **x** | **x** | **x** | **x** | **x** | **x** | **x** | **C5** | **x** |
|  | **x** | **x** | **II** | **x** | **x** |  | **x** | **x** |  |  |  |  | **x** | **x** |  | **x** | **x** | **x** | **x** | **x** | **C5** | **x** |
|  | **x** | **x** | **III** |  | **x** |  |  | **x** |  |  |  |  |  |  |  | **x** | **x** | **x** |  | **x** | **C5** | **x** |
|  | **x** | **x** | **IV** |  | **x** |  |  | **x** |  |  |  |  |  |  |  |  |  |  |  |  | **IC4** | **x** |

S2.1.3 Land-use variables

*Table S2.1.3: Incorporated* ***land-use*** *predictors (x) for each species and model setup (buffered, sampled, selection). The predictor with the highest contribution is highlighted in red, the number represents the overall rank. Predictors representing the highest interaction are highlighted in green, predictors involved in both (contribution and interaction) are highlighted in purple.*

| **Species** | **Buffered** | **Sampled** | **Selection** | **TBET** | **TDT** | **ECT** | **DCT** | **CS** | **DS** | **C3** | **C4** | **T** | **S** | **NIC** | **IC** | **U** | **B** |
| --- | --- | --- | --- | --- | --- | --- | --- | --- | --- | --- | --- | --- | --- | --- | --- | --- | --- |
| **atroparvus** |  |  | **I** | **x** | **x** | **x** | **x** | **x** | **x** | **x** | **x** | **x** | **x** | **x** | **x** | **x** | **C3** |
|  |  |  | **II** | **I** | **x** | **x** |  | **x** | **x** | **x** | **x** |  | **x** | **x** | **x** | **x** | **C2** |
|  |  |  | **III** | **x** | **x** | **x** |  | **x** | **x** | **x** | **x** |  | **x** | **I** | **x** | **x** | **C3** |
|  |  |  | **IV** | **I** | **x** | **x** |  | **x** | **x** | **x** | **x** |  | **x** | **x** | **x** | **x** | **C2** |
|  | **x** |  | **I** | **x** | **x** | **x** | **x** | **x** | **x** | **x** | **x** | **x** | **x** | **x** | **x** | **x** | **C4** |
|  | **x** |  | **II** | **x** | **x** | **x** | **x** | **x** | **x** | **x** | **x** |  | **x** | **x** | **x** | **x** | **C2** |
|  | **x** |  | **III** | **x** | **x** | **x** | **x** | **x** | **x** | **x** | **x** |  | **x** | **x** | **x** | **x** | **C3** |
|  | **x** |  | **IV** | **x** | **x** | **x** | **x** | **x** | **x** | **x** | **x** |  | **x** | **x** | **x** | **x** | **C2** |
|  |  | **x** | **I** | **x** | **x** | **x** | **x** | **x** | **x** | **x** | **x** | **x** | **x** | **x** | **x** | **x** | **C1** |
|  |  | **x** | **II** | **x** | **x** | **x** |  | **x** | **I** | **x** | **x** |  |  | **x** | **x** | **x** | **C1** |
|  |  | **x** | **III** | **x** | **x** | **x** |  | **x** | **x** | **x** | **x** |  |  | **x** | **x** | **x** | **C1** |
|  |  | **x** | **IV** | **I** | **x** | **x** |  | **x** | **x** | **x** | **x** |  |  | **x** | **x** | **x** | **C2** |
|  | **x** | **x** | **I** | **x** | **x** | **x** | **x** | **x** | **x** | **x** | **x** | **x** | **x** | **x** | **x** | **x** | **C2** |
|  | **x** | **x** | **II** | **x** | **x** | **x** |  | **x** | **x** | **x** | **x** |  | **x** | **x** | **x** | **x** | **C2** |
|  | **x** | **x** | **III** | **x** | **x** | **x** |  | **x** | **I** | **x** | **x** |  | **x** | **x** | **x** | **x** | **C2** |
|  | **x** | **x** | **IV** | **x** | **x** | **x** |  | **x** | **x** | **x** | **x** |  | **x** | **x** | **x** | **x** | **C2** |
| **labranchiae** |  |  | **I** | **C3** | **x** | **x** | **x** | **x** | **x** | **x** | **x** | **x** | **x** | **x** | **x** | **x** | **x** |
|  |  |  | **II** | **C5** | **x** | **x** |  | **x** | **x** | **x** | **x** |  |  | **x** | **x** | **x** | **x** |
|  |  |  | **III** | **C4** | **x** | **x** |  | **x** | **x** | **x** | **x** |  |  | **x** | **x** | **x** | **x** |
|  |  |  | **IV** | **C3** | **x** | **x** |  | **x** | **x** | **x** | **x** |  |  | **x** | **x** | **x** | **x** |
|  | **x** |  | **I** | **C3** | **x** | **x** | **x** | **x** | **x** | **x** | **x** | **x** | **x** | **x** | **x** | **x** | **x** |
|  | **x** |  | **II** | **C3** | **x** | **x** |  | **x** | **x** | **x** | **x** | **x** |  | **x** | **x** | **x** | **x** |
|  | **x** |  | **III** | **C4** | **x** | **x** |  | **x** | **x** | **x** | **x** | **x** |  | **x** | **x** | **x** | **x** |
|  | **x** |  | **IV** | **C3** | **x** | **x** |  | **x** | **x** | **x** | **x** | **x** |  | **x** | **x** | **x** | **x** |
|  |  | **x** | **I** | **C4** | **x** | **x** | **x** | **x** | **x** | **x** | **x** | **x** | **x** | **x** | **x** | **x** | **x** |
|  |  | **x** | **II** | **C4** | **x** | **x** |  | **x** | **x** | **x** | **x** |  |  | **x** | **x** | **x** | **x** |
|  |  | **x** | **III** | **C4** | **x** | **x** |  | **x** | **x** | **x** | **x** |  |  | **x** | **x** | **x** | **x** |
|  |  | **x** | **IV** | **C4** | **x** | **x** |  | **x** | **x** | **x** | **x** |  |  | **x** | **x** | **x** | **x** |
|  | **x** | **x** | **I** | **C4** | **x** | **x** | **x** | **x** | **x** | **x** | **x** | **x** | **x** | **x** | **x** | **x** | **x** |
|  | **x** | **x** | **II** | **C4** | **x** | **x** |  | **x** | **x** | **x** | **x** | **x** | **x** | **x** | **x** | **x** | **x** |
|  | **x** | **x** | **III** | **C4** | **x** | **x** |  | **x** | **x** | **x** | **x** | **x** | **x** | **x** | **x** | **x** | **x** |
|  | **x** | **x** | **IV** | **C4** | **x** | **x** |  | **x** | **x** | **x** | **x** | **x** | **x** | **x** | **x** | **x** | **x** |
| **messeae** |  |  | **I** | **x** | **x** | **x** | **x** | **x** | **x** | **x** | **x** | **x** | **x** | **x** | **x** | **x** | **C4** |
|  |  |  | **II** | **x** | **x** | **x** | **x** | **x** | **x** | **x** | **x** |  | **x** | **x** | **x** | **x** | **C4** |
|  |  |  | **III** | **x** | **x** | **I** | **x** | **x** | **x** | **x** | **x** |  | **x** | **x** | **x** | **x** | **C4** |
|  |  |  | **IV** | **x** | **x** | **x** | **x** | **x** | **x** | **x** | **x** |  | **x** | **x** | **x** | **x** | **C6** |
|  | **x** |  | **I** | **x** | **x** | **x** | **x** | **x** | **x** | **x** | **x** | **x** | **x** | **x** | **x** | **x** | **C4** |
|  | **x** |  | **II** | **x** | **x** | **x** | **x** | **x** | **x** | **x** | **x** |  | **x** | **x** | **x** | **x** | **C4** |
|  | **x** |  | **III** | **x** | **x** | **x** | **x** | **x** | **x** | **x** | **x** |  | **x** | **x** | **x** | **x** | **C8** |
|  | **x** |  | **IV** | **x** | **x** | **x** | **x** | **x** | **x** | **x** | **x** |  | **x** | **x** | **x** | **x** | **C4** |
|  |  | **x** | **I** | **x** | **x** | **x** | **x** | **x** | **x** | **x** | **x** | **x** | **x** | **x** | **x** | **x** | **C2** |
|  |  | **x** | **II** | **x** | **x** | **x** |  |  | **x** | **I** | **x** |  | **x** | **x** | **x** | **x** | **C2** |
|  |  | **x** | **III** | **x** | **x** | **x** |  |  | **x** | **x** | **I** |  | **x** | **x** | **x** | **x** | **C2** |
|  |  | **x** | **IV** | **x** | **x** | **I** |  |  | **x** | **x** | **x** |  | **x** | **x** | **x** | **x** | **C2** |
|  | **x** | **x** | **I** | **x** | **x** | **x** | **x** | **x** | **x** | **x** | **x** | **x** | **x** | **x** | **x** | **x** | **C2** |
|  | **x** | **x** | **II** | **x** | **x** | **x** | **x** | **x** | **x** | **x** | **x** |  | **x** | **x** |  | **x** | **C2** |
|  | **x** | **x** | **III** | **x** | **x** | **x** | **x** | **x** | **x** | **x** | **x** |  | **x** | **x** |  | **x** | **C2** |
|  | **x** | **x** | **IV** | **x** | **x** | **x** | **x** | **x** | **x** | **x** | **x** |  | **x** | **x** |  | **x** | **C2** |
| **sacharovi** |  |  | **I** | **x** | **x** | **x** | **x** | **x** | **x** | **x** | **C2** | **x** | **x** | **x** | **x** | **x** | **x** |
|  |  |  | **II** | **x** | **x** | **x** |  | **x** | **x** | **x** | **C3** |  |  | **x** | **x** | **x** | **x** |
|  |  |  | **III** | **x** | **x** | **x** |  | **I** | **x** | **x** | **x** |  |  | **x** | **C3** | **x** | **x** |
|  |  |  | **IV** | **x** | **x** | **x** |  | **x** | **x** | **x** | **C4** |  |  | **x** | **x** | **x** | **x** |
|  | **x** |  | **I** | **x** | **x** | **x** | **x** | **x** | **x** | **x** | **C3** | **x** | **x** | **x** | **x** | **x** | **x** |
|  | **x** |  | **II** | **x** | **x** | **x** | **x** | **x** | **x** | **x** | **C3** |  |  | **x** | **x** | **x** | **x** |
|  | **x** |  | **III** | **x** | **x** | **x** | **x** | **x** | **x** | **x** | **C2** |  |  | **x** | **x** | **x** | **x** |
|  | **x** |  | **IV** | **x** | **x** | **x** | **x** | **x** | **x** | **x** | **C3** |  |  | **x** | **x** | **x** | **x** |
|  |  | **x** | **I** | **x** | **x** | **x** | **x** | **x** | **x** | **x** | **C3** | **x** | **x** | **x** | **x** | **x** | **x** |
|  |  | **x** | **II** |  | **x** | **x** |  | **x** | **x** | **x** | **C3** |  |  | **x** | **x** | **x** | **x** |
|  |  | **x** | **III** |  | **x** | **x** |  | **x** | **x** | **I** | **C3** |  |  | **x** | **x** | **x** | **x** |
|  |  | **x** | **IV** |  | **x** | **x** |  | **x** | **x** | **x** | **C3** |  |  | **x** | **x** | **x** | **x** |
|  | **x** | **x** | **I** | **x** | **x** | **x** | **x** | **x** | **x** | **x** | **C3** | **x** | **x** | **x** | **x** | **x** | **x** |
|  | **x** | **x** | **II** | **x** | **x** | **x** | **x** | **x** | **x** | **x** | **C3** |  | **x** | **x** | **x** | **x** | **x** |
|  | **x** | **x** | **III** | **x** | **x** | **x** | **x** | **x** | **x** | **x** | **C3** |  | **x** | **x** | **x** | **x** | **x** |
|  | **x** | **x** | **IV** | **I** | **x** | **x** | **x** | **x** | **x** | **x** | **C3** |  | **x** | **x** | **x** | **x** | **x** |
| **sergentii** |  |  | **I** | **x** | **x** | **x** | **x** | **x** | **x** | **x** | **x** | **x** | **x** | **x** | **x** | **x** | **C6** |
|  |  |  | **II** | **x** | **x** | **x** |  | **x** | **x** | **x** | **x** |  |  | **x** | **x** | **x** | **C5** |
|  |  |  | **III** | **x** | **x** | **x** |  | **I** | **x** | **x** | **x** |  |  | **I** | **x** | **x** | **C6** |
|  |  |  | **IV** | **x** | **x** | **x** |  | **x** | **x** | **x** | **x** |  |  | **x** | **x** | **x** | **C4** |
|  | **x** |  | **I** | **x** | **x** | **x** | **x** | **x** | **x** | **x** | **x** | **x** | **x** | **x** | **x** | **I** | **C6** |
|  | **x** |  | **II** | **x** | **x** | **x** |  | **x** | **x** | **x** | **x** |  |  | **x** | **x** | **x** | **C8** |
|  | **x** |  | **III** | **x** | **x** | **x** |  | **x** | **x** | **x** | **x** |  |  | **x** | **x** | **I** | **C6** |
|  | **x** |  | **IV** | **x** | **x** | **x** |  | **x** | **x** | **x** | **x** |  |  | **x** | **x** | **x** | **C4** |
|  |  | **x** | **I** | **x** | **x** | **x** | **x** | **I** | **x** | **x** | **x** | **x** | **x** | **I** | **x** | **x** | **C5** |
|  |  | **x** | **II** | **x** | **x** | **x** |  | **x** | **x** | **I** | **x** | **x** | **x** | **x** | **x** | **x** | **IC5** |
|  |  | **x** | **III** | **x** | **x** | **x** |  | **I** | **x** | **x** | **x** |  |  | **x** | **x** | **x** | **C5** |
|  |  | **x** | **IV** | **x** | **x** | **x** |  | **I** | **x** | **x** | **x** |  |  | **x** | **x** | **x** | **C4** |
|  | **x** | **x** | **I** | **x** | **x** | **x** | **x** | **I** | **x** | **x** | **x** | **x** | **x** | **I** | **x** | **x** | **C5** |
|  | **x** | **x** | **II** | **x** | **x** | **x** |  | **x** | **x** | **I** | **x** |  |  | **x** | **x** | **x** | **IC5** |
|  | **x** | **x** | **III** | **x** | **x** | **x** |  | **I** | **x** | **x** | **x** |  |  | **x** | **x** | **x** | **C5** |
|  | **x** | **x** | **IV** | **x** | **x** | **x** |  | **I** | **x** | **x** | **x** |  |  | **x** | **x** | **x** | **C4** |
| **superpictus** |  |  | **I** | **x** | **x** | **x** | **x** | **x** | **C10** | **x** | **x** | **x** | **x** | **x** | **x** | **x** | **x** |
|  |  |  | **II** | **x** | **x** | **x** |  | **x** | **x** | **x** | **C12** |  | **x** | **x** | **x** | **x** | **I** |
|  |  |  | **III** | **x** | **x** | **x** |  | **x** | **x** | **x** | **C12** |  | **x** | **x** | **x** | **x** | **I** |
|  |  |  | **IV** | **x** | **x** | **x** |  | **x** | **C9** | **x** | **x** |  | **x** | **I** | **x** | **x** | **x** |
|  | **x** |  | **I** | **x** | **x** | **x** | **x** | **x** | **C10** | **x** | **x** | **x** | **x** | **x** | **x** | **x** | **x** |
|  | **x** |  | **II** | **x** | **x** | **x** |  | **x** | **x** | **x** | **C12** |  | **x** | **x** | **x** | **x** | **x** |
|  | **x** |  | **III** | **x** | **x** | **x** |  | **x** | **x** | **x** | **C12** |  | **x** | **x** | **x** | **x** | **I** |
|  | **x** |  | **IV** | **x** | **x** | **x** |  | **x** | **C9** | **x** | **x** |  | **x** | **I** | **x** | **x** | **I** |
|  |  | **x** | **I** | **x** | **x** | **x** | **x** | **x** | **x** | **x** | **C9** | **x** | **x** | **I** | **x** | **x** | **x** |
|  |  | **x** | **II** | **x** | **x** | **x** |  | **x** | **x** | **x** | **C8** |  |  | **x** | **x** | **x** | **x** |
|  |  | **x** | **III** | **x** | **x** | **x** |  | **x** | **x** | **x** | **C9** |  |  | **x** | **x** | **x** | **x** |
|  |  | **x** | **IV** | **x** | **x** | **x** |  | **x** | **x** | **x** | **C10** |  |  | **x** | **x** | **x** | **x** |
|  | **x** | **x** | **I** | **x** | **x** | **x** | **x** | **x** | **x** | **x** | **C9** | **x** | **x** | **I** | **I** | **x** | **x** |
|  | **x** | **x** | **II** | **x** | **x** | **x** |  | **x** | **x** |  | **C11** |  |  | **x** | **x** | **x** | **x** |
|  | **x** | **x** | **III** | **x** | **x** | **x** |  | **x** | **x** |  | **C9** |  |  | **x** | **x** | **x** | **x** |
|  | **x** | **x** | **IV** | **x** | **x** | **x** |  | **x** | **x** |  | **C10** |  |  | **x** | **x** | **x** | **x** |

S2.2 Skills and Scores

S2.2.1 *An. atroparvus*

*Table S2.2.1: Skill, transferability and score for each model run of* ***An. atroparvus****. Skill is described by the True Skill Statistics (TSS), the Area Under the Curve (AUC; spatial sorting bias removed AUC.SSB), Cohens Kappa (KAPPA; spatial sorting bias removed KAPPA.SSB), Root Mean Squared Error (RMSE), Mean Square Error Skill Score (MSESS), Brier Skill (BS) and Brier Skill Score (BSS). The transferability is represented by the Area of Applicability (AoA). The overall score (SCORE) represents the relationship between skill and transferability.* *The best model setup is highlighted in red.*

| **Species** | **Buffered** | **Sampled** | **Selection** | **TSS** | **AUC** | **KAPPA** | **AUC.SSB** | **KAPPA.SSB** | **RMSE** | **MSESS** | **BS** | **BSS** | **AoA** | **SCORE** |
| --- | --- | --- | --- | --- | --- | --- | --- | --- | --- | --- | --- | --- | --- | --- |
| ***atroparvus*** |  |  | **I** | .8991 | .9694 | .8577 | .6950 | .5488 | .1628 | .1323 | .0549 | .7533 | .8492 | .7891 |
|  |  |  | **II** | .8973 | .9694 | .8581 | .7000 | .5518 | .1608 | .1306 | .0575 | .7416 | .7874 | .7557 |
|  |  |  | **III** | .8970 | .9696 | .8573 | .6932 | .5434 | .1610 | .1307 | .0578 | .7403 | .8473 | .7855 |
|  |  |  | **IV** | .8933 | .9676 | .8559 | .6858 | .5295 | .1629 | .1355 | .0579 | .7398 | .7982 | .7564 |
|  | **x** |  | **I** | .8649 | .9338 | .7971 | .5230 | .4118 | .2474 | .2491 | .0822 | .6306 | .7417 | .6780 |
|  | **x** |  | **II** | .8627 | .9326 | .7957 | .5114 | .4037 | .2464 | .2483 | .0847 | .6194 | .8798 | .7527 |
|  | **x** |  | **III** | .8606 | .9334 | .7917 | .5256 | .4114 | .2452 | .2481 | .0864 | .6117 | .8884 | .7590 |
|  | **x** |  | **IV** | .8602 | .9286 | .7913 | .4946 | .3900 | .2458 | .2490 | .0866 | .6108 | .7335 | .6651 |
|  |  | **x** | **I** | .8925 | .9688 | .8567 | .7086 | .5566 | .1607 | .1314 | .0584 | .7376 | .3848 | .5796 |
|  |  | **x** | **II** | .8953 | .9692 | .8572 | .6978 | .5464 | .1615 | .1308 | .0590 | .7349 | .3387 | .5617 |
|  |  | **x** | **III** | .8970 | .9690 | .8574 | .6926 | .5433 | .1615 | .1304 | .0565 | .7461 | .4026 | .5832 |
|  |  | **x** | **IV** | .8901 | .9678 | .8554 | .6862 | .5384 | .1623 | .1342 | .0619 | .7218 | .3837 | .5701 |
|  | **x** | **x** | **I** | .8658 | .9346 | .7984 | .5292 | .4124 | .2412 | .2392 | .0819 | .6320 | .7715 | .6954 |
|  | **x** | **x** | **II** | .8633 | .9330 | .7947 | .5002 | .3891 | .2413 | .2403 | .0855 | .6158 | .7584 | .6804 |
|  | **x** | **x** | **III** | .8652 | .9306 | .7967 | .4852 | .3749 | .2432 | .2417 | .0828 | .6279 | .7577 | .6783 |
|  | **x** | **x** | **IV** | .8631 | .9306 | .7941 | .4894 | .3808 | .2432 | .2428 | .0834 | .6252 | .7870 | .6953 |

S2.2.2 *An. labranchiae*

*Table S2.2.2: Skill, transferability and score for each model run of* ***An. labranchiae****. Skill is described by the True Skill Statistics (TSS), the Area Under the Curve (AUC; spatial sorting bias removed AUC.SSB), Cohens Kappa (KAPPA; spatial sorting bias removed KAPPA.SSB), Root Mean Squared Error (RMSE), Mean Square Error Skill Score (MSESS), Brier Skill (BS) and Brier Skill Score (BSS). The transferability is represented by the Area of Applicability (AoA). The overall score (SCORE) represents the relationship between skill and transferability. The best model setup is highlighted in red.*

| **Species** | **Buffered** | **Sampled** | **Selection** | **TSS** | **AUC** | **KAPPA** | **AUC.SSB** | **KAPPA.SSB** | **RMSE** | **MSESS** | **BS** | **BSS** | **AoA** | **SCORE** |
| --- | --- | --- | --- | --- | --- | --- | --- | --- | --- | --- | --- | --- | --- | --- |
| ***labranchiae*** |  |  | **I** | .9712 | .9888 | .7092 | .5500 | .4420 | .1098 | .1962 | .0183 | .6583 | .9180 | .7976 |
|  |  |  | **II** | .9689 | .9884 | .6922 | .5654 | .4246 | .1100 | .1984 | .0216 | .5967 | .9217 | .7930 |
|  |  |  | **III** | .9668 | .9878 | .6974 | .5492 | .4256 | .1115 | .2034 | .0208 | .6117 | .9230 | .7935 |
|  |  |  | **IV** | .9605 | .9852 | .6716 | .5270 | .4109 | .1159 | .2211 | .0254 | .5258 | .9398 | .7907 |
|  | **x** |  | **I** | .9679 | .9834 | .6201 | .5066 | .3886 | .1367 | .2568 | .0243 | .5463 | .9202 | .7777 |
|  | **x** |  | **II** | .9691 | .9832 | .6146 | .4894 | .3647 | .1360 | .2537 | .0218 | .5930 | .9280 | .7829 |
|  | **x** |  | **III** | .9670 | .9830 | .6106 | .4724 | .3573 | .1363 | .2564 | .0239 | .5538 | .9261 | .7755 |
|  | **x** |  | **IV** | .9656 | .9828 | .6158 | .4766 | .3691 | .1389 | .2667 | .0257 | .5202 | .9415 | .7830 |
|  |  | **x** | **I** | .9708 | .9892 | .7162 | .5248 | .4063 | .1001 | .1717 | .0194 | .6378 | .8711 | .7622 |
|  |  | **x** | **II** | .9685 | .9888 | .7058 | .5404 | .4243 | .0992 | .1732 | .0216 | .5967 | .8830 | .7680 |
|  |  | **x** | **III** | .9690 | .9888 | .7119 | .5288 | .4139 | .1000 | .1738 | .0192 | .6415 | .9089 | .7853 |
|  |  | **x** | **IV** | .9663 | .9876 | .7057 | .5374 | .4303 | .1049 | .1882 | .0226 | .5781 | .8607 | .7534 |
|  | **x** | **x** | **I** | .9683 | .9846 | .6328 | .4852 | .3766 | .1242 | .2231 | .0248 | .5370 | .8871 | .7540 |
|  | **x** | **x** | **II** | .9702 | .9842 | .6234 | .4700 | .3636 | .1240 | .2205 | .0227 | .5762 | .8992 | .7624 |
|  | **x** | **x** | **III** | .9693 | .9844 | .6327 | .4896 | .3754 | .1244 | .2226 | .0228 | .5743 | .8918 | .7609 |
|  | **x** | **x** | **IV** | .9677 | .9842 | .6347 | .4900 | .3805 | .1231 | .2221 | .0241 | .5501 | .8148 | .7143 |

S2.2.3 *An. messeae*

*Table S2.2.3: Skill, transferability and score for each model run of* ***An. messeae****. Skill is described by the True Skill Statistics (TSS), the Area Under the Curve (AUC; spatial sorting bias removed AUC.SSB), Cohens Kappa (KAPPA; spatial sorting bias removed KAPPA.SSB), Root Mean Squared Error (RMSE), Mean Square Error Skill Score (MSESS), Brier Skill (BS) and Brier Skill Score (BSS). The transferability is represented by the Area of Applicability (AoA). The overall score (SCORE) represents the relationship between skill and transferability. The best model setup is highlighted in red.*

| **Species** | **Buffered** | **Sampled** | **Selection** | **TSS** | **AUC** | **KAPPA** | **AUC.SSB** | **KAPPA.SSB** | **RMSE** | **MSESS** | **BS** | **BSS** | **AoA** | **SCORE** |
| --- | --- | --- | --- | --- | --- | --- | --- | --- | --- | --- | --- | --- | --- | --- |
| ***messeae*** |  |  | **I** | .9345 | .9842 | .9106 | .7328 | .5828 | .1559 | .1137 | .0355 | .8431 | .5834 | .6850 |
|  |  |  | **II** | .9363 | .9844 | .9121 | .7338 | .5827 | .1555 | .1127 | .0354 | .8435 | .5706 | .6800 |
|  |  |  | **III** | .9320 | .9842 | .9093 | .7450 | .5990 | .1545 | .1131 | .0379 | .8324 | .5683 | .6808 |
|  |  |  | **IV** | .9311 | .9830 | .9059 | .7256 | .5856 | .1563 | .1154 | .0384 | .8302 | .5420 | .6647 |
|  | **x** |  | **I** | .9138 | .9518 | .8676 | .4754 | .3959 | .1987 | .1684 | .0511 | .7741 | .7291 | .6859 |
|  | **x** |  | **II** | .9133 | .9502 | .8674 | .4428 | .3699 | .1979 | .1675 | .0522 | .7692 | .7064 | .6664 |
|  | **x** |  | **III** | .9132 | .9490 | .8661 | .4338 | .3783 | .2005 | .1706 | .0520 | .7701 | .6329 | .6284 |
|  | **x** |  | **IV** | .9152 | .9512 | .8701 | .4592 | .3938 | .1963 | .1649 | .0510 | .7745 | .6532 | .6445 |
|  |  | **x** | **I** | .9330 | .9844 | .9091 | .7406 | .5895 | .1551 | .1130 | .0361 | .8404 | .2505 | .5765 |
|  |  | **x** | **II** | .9309 | .9840 | .9070 | .7392 | .5898 | .1552 | .1138 | .0372 | .8355 | .2418 | .5733 |
|  |  | **x** | **III** | .9309 | .9838 | .9073 | .7276 | .5779 | .1551 | .1138 | .0383 | .8307 | .2566 | .5718 |
|  |  | **x** | **IV** | .9299 | .9834 | .9057 | .7336 | .5844 | .1551 | .1141 | .0389 | .8280 | .2530 | .5724 |
|  | **x** | **x** | **I** | .9155 | .9552 | .8721 | .4962 | .4223 | .1960 | .1639 | .0509 | .7750 | .5900 | .6219 |
|  | **x** | **x** | **II** | .9154 | .9544 | .8714 | .4326 | .3687 | .1962 | .1645 | .0510 | .7745 | .6178 | .6203 |
|  | **x** | **x** | **III** | .9149 | .9526 | .8694 | .4622 | .3837 | .1969 | .1652 | .0504 | .7772 | .5845 | .6100 |
|  | **x** | **x** | **IV** | .9153 | .9534 | .8717 | .4670 | .3859 | .1964 | .1649 | .0491 | .7829 | .5972 | .6178 |

S2.2.4 *An. sacharovi*

*Table S2.2.4: Skill, transferability and score for each model run of* ***An. sacharovi****. Skill is described by the True Skill Statistics (TSS), the Area Under the Curve (AUC; spatial sorting bias removed AUC.SSB), Cohens Kappa (KAPPA; spatial sorting bias removed KAPPA.SSB), Root Mean Squared Error (RMSE), Mean Square Error Skill Score (MSESS), Brier Skill (BS) and Brier Skill Score (BSS). The transferability is represented by the Area of Applicability (AoA). The overall score (SCORE) represents the relationship between skill and transferability. The best model setup is highlighted in red.*

| **Species** | **Buffered** | **Sampled** | **Selection** | **TSS** | **AUC** | **KAPPA** | **AUC.SSB** | **KAPPA.SSB** | **RMSE** | **MSESS** | **BS** | **BSS** | **AoA** | **SCORE** |
| --- | --- | --- | --- | --- | --- | --- | --- | --- | --- | --- | --- | --- | --- | --- |
| ***sacharovi*** |  |  | **I** | .9346 | .9738 | .7154 | .5098 | .3874 | .1649 | .2129 | .0430 | .6194 | .8241 | .7262 |
|  |  |  | **II** | .9329 | .9740 | .7116 | .5216 | .3946 | .1643 | .2133 | .0480 | .5752 | .8169 | .7193 |
|  |  |  | **III** | .9364 | .9734 | .7171 | .4980 | .3842 | .1643 | .2109 | .0442 | .6088 | .8342 | .7294 |
|  |  |  | **IV** | .9305 | .9722 | .7061 | .5150 | .3969 | .1669 | .2194 | .0470 | .5840 | .7947 | .7069 |
|  | **x** |  | **I** | .9289 | .9582 | .6219 | .4028 | .2871 | .2014 | .2682 | .0548 | .5150 | .8402 | .7037 |
|  | **x** |  | **II** | .9289 | .9574 | .6214 | .4104 | .3027 | .2007 | .2679 | .0539 | .5229 | .8399 | .7065 |
|  | **x** |  | **III** | .9277 | .9598 | .6238 | .4180 | .2991 | .2011 | .2686 | .0544 | .5185 | .8364 | .7043 |
|  | **x** |  | **IV** | .9260 | .9560 | .6140 | .3876 | .2710 | .2017 | .2718 | .0562 | .5026 | .8340 | .6956 |
|  |  | **x** | **I** | .9280 | .9694 | .6927 | .4662 | .3652 | .1534 | .1952 | .0490 | .5663 | .8006 | .6996 |
|  |  | **x** | **II** | .9296 | .9690 | .6883 | .4606 | .3449 | .1543 | .1966 | .0464 | .5893 | .7869 | .6917 |
|  |  | **x** | **III** | .9286 | .9692 | .6854 | .4508 | .3477 | .1540 | .1965 | .0471 | .5831 | .8277 | .7137 |
|  |  | **x** | **IV** | .9250 | .9668 | .6801 | .4420 | .3398 | .1574 | .2045 | .0482 | .5734 | .8125 | .7018 |
|  | **x** | **x** | **I** | .9301 | .9586 | .6309 | .3922 | .2939 | .1897 | .2472 | .0543 | .5194 | .8207 | .6923 |
|  | **x** | **x** | **II** | .9299 | .9582 | .6302 | .3966 | .2909 | .1905 | .2486 | .0534 | .5274 | .8375 | .7032 |
|  | **x** | **x** | **III** | .9325 | .9590 | .6310 | .4050 | .2985 | .1904 | .2469 | .0517 | .5424 | .8251 | .6991 |
|  | **x** | **x** | **IV** | .9297 | .9574 | .6224 | .3990 | .2872 | .1930 | .2531 | .0544 | .5185 | .8276 | .6963 |

S2.2.5 *An. sergentii*

*Table S2.2.5: Skill, transferability and score for each model run of* ***An. sergentii****. Skill is described by the True Skill Statistics (TSS), the Area Under the Curve (AUC; spatial sorting bias removed AUC.SSB), Cohens Kappa (KAPPA; spatial sorting bias removed KAPPA.SSB), Root Mean Squared Error (RMSE), Mean Square Error Skill Score (MSESS), Brier Skill (BS) and Brier Skill Score (BSS). The transferability is represented by the Area of Applicability (AoA). The overall score (SCORE) represents the relationship between skill and transferability. The best model setup is highlighted in red.*

| **Species** | **Buffered** | **Sampled** | **Selection** | **TSS** | **AUC** | **KAPPA** | **AUC.SSB** | **KAPPA.SSB** | **RMSE** | **MSESS** | **BS** | **BSS** | **AoA** | **SCORE** |
| --- | --- | --- | --- | --- | --- | --- | --- | --- | --- | --- | --- | --- | --- | --- |
| ***sergentii*** |  |  | **I** | .9635 | .9884 | .8882 | .6482 | .5117 | .1307 | .1133 | .0253 | .8266 | .3960 | .5919 |
|  |  |  | **II** | .9629 | .9882 | .8867 | .6414 | .5071 | .1318 | .1152 | .0247 | .8307 | .3088 | .5641 |
|  |  |  | **III** | .9626 | .9876 | .8858 | .6306 | .4920 | .1316 | .1149 | .0246 | .8314 | .3365 | .5679 |
|  |  |  | **IV** | .9621 | .9876 | .8823 | .6174 | .4773 | .1330 | .1177 | .0239 | .8362 | .3991 | .5841 |
|  | **x** |  | **I** | .9649 | .9800 | .8705 | .4998 | .3978 | .1449 | .1316 | .0257 | .8238 | .3532 | .5366 |
|  | **x** |  | **II** | .9649 | .9802 | .8705 | .4854 | .3759 | .1449 | .1313 | .0251 | .8279 | .3663 | .5359 |
|  | **x** |  | **III** | .9639 | .9802 | .8681 | .4840 | .3770 | .1463 | .1340 | .0263 | .8197 | .3821 | .5400 |
|  | **x** |  | **IV** | .9645 | .9800 | .8703 | .4894 | .3827 | .1466 | .1342 | .0249 | .8293 | .3918 | .5467 |
|  |  | **x** | **I** | .9600 | .9874 | .8810 | .6726 | .5084 | .1438 | .1345 | .0267 | .8170 | .6213 | .6830 |
|  |  | **x** | **II** | .9607 | .9872 | .8805 | .6542 | .5097 | .1434 | .1336 | .0262 | .8204 | .6116 | .6768 |
|  |  | **x** | **III** | .9598 | .9874 | .8826 | .6582 | .5052 | .1439 | .1352 | .0281 | .8074 | .5459 | .6460 |
|  |  | **x** | **IV** | .9564 | .9858 | .8786 | .6390 | .4976 | .1500 | .1467 | .0268 | .8163 | .6642 | .6964 |
|  | **x** | **x** | **I** | .9600 | .9874 | .8810 | .6524 | .4981 | .1438 | .1345 | .0267 | .8170 | .6248 | .6805 |
|  | **x** | **x** | **II** | .9607 | .9872 | .8805 | .6598 | .5139 | .1434 | .1336 | .0262 | .8204 | .6079 | .6765 |
|  | **x** | **x** | **III** | .9598 | .9874 | .8826 | .6616 | .5122 | .1439 | .1352 | .0281 | .8074 | .5926 | .6677 |
|  | **x** | **x** | **IV** | .9564 | .9858 | .8786 | .6308 | .4902 | .1500 | .1467 | .0268 | .8163 | .6506 | .6880 |

S2.2.6 *An. superpictus*

*Table S2.2.6: Skill, transferability and score for each model run of* ***An. superpictus****. Skill is described by the True Skill Statistics (TSS), the Area Under the Curve (AUC; spatial sorting bias removed AUC.SSB), Cohens Kappa (KAPPA; spatial sorting bias removed KAPPA.SSB), Root Mean Squared Error (RMSE), Mean Square Error Skill Score (MSESS), Brier Skill (BS) and Brier Skill Score (BSS). The transferability is represented by the Area of Applicability (AoA). The overall score (SCORE) represents the relationship between skill and transferability. The best model setup is highlighted in red.*

| **Species** | **Buffered** | **Sampled** | **Selection** | **TSS** | **AUC** | **KAPPA** | **AUC.SSB** | **KAPPA.SSB** | **RMSE** | **MSESS** | **BS** | **BSS** | **AoA** | **SCORE** |
| --- | --- | --- | --- | --- | --- | --- | --- | --- | --- | --- | --- | --- | --- | --- |
| ***superpictus*** |  |  | **I** | .9461 | .9806 | .8005 | .5730 | .4444 | .1512 | .1680 | .0389 | .6964 | .7514 | .7095 |
|  |  |  | **II** | .9470 | .9808 | .8028 | .5940 | .4588 | .1505 | .1664 | .0362 | .7175 | .7529 | .7170 |
|  |  |  | **III** | .9462 | .9804 | .8019 | .5762 | .4499 | .1513 | .1675 | .0365 | .7152 | .7579 | .7162 |
|  |  |  | **IV** | .9385 | .9784 | .7892 | .5594 | .4311 | .1592 | .1855 | .0407 | .6824 | .7788 | .7186 |
|  | **x** |  | **I** | .9226 | .9806 | .7010 | .6086 | .2759 | .1918 | .2387 | .0579 | .5482 | .9285 | .7524 |
|  | **x** |  | **II** | .9291 | .9808 | .7126 | .6028 | .2742 | .1859 | .2267 | .0561 | .5622 | .7542 | .6514 |
|  | **x** |  | **III** | .9311 | .9804 | .7101 | .6050 | .2562 | .1870 | .2257 | .0539 | .5794 | .7755 | .6596 |
|  | **x** |  | **IV** | .9190 | .9784 | .7010 | .5986 | .2735 | .1950 | .2479 | .0587 | .5419 | .9611 | .7739 |
|  |  | **x** | **I** | .9491 | .9438 | .8110 | .5902 | .4754 | .1422 | .1530 | .0341 | .7339 | .7064 | .6991 |
|  |  | **x** | **II** | .9489 | .9594 | .8083 | .5794 | .4603 | .1425 | .1536 | .0341 | .7339 | .7349 | .7111 |
|  |  | **x** | **III** | .9488 | .9582 | .8088 | .5824 | .4582 | .1424 | .1538 | .0342 | .7331 | .7236 | .7052 |
|  |  | **x** | **IV** | .9476 | .9458 | .8047 | .5814 | .4549 | .1451 | .1582 | .0344 | .7316 | .6611 | .6722 |
|  | **x** | **x** | **I** | .9461 | .9816 | .8005 | .3326 | .4548 | .1512 | .1680 | .0389 | .6964 | .7759 | .7258 |
|  | **x** | **x** | **II** | .9470 | .9810 | .8028 | .3506 | .4436 | .1505 | .1664 | .0362 | .7175 | .7445 | .7091 |
|  | **x** | **x** | **III** | .9462 | .9816 | .8019 | .3074 | .4470 | .1513 | .1675 | .0365 | .7152 | .7135 | .6934 |
|  | **x** | **x** | **IV** | .9385 | .9808 | .7892 | .3600 | .4478 | .1592 | .1855 | .0407 | .6824 | .7767 | .7219 |

S2.3 Comparisons between best model setup and observations

S.2.3.1 *An. atroparvus*


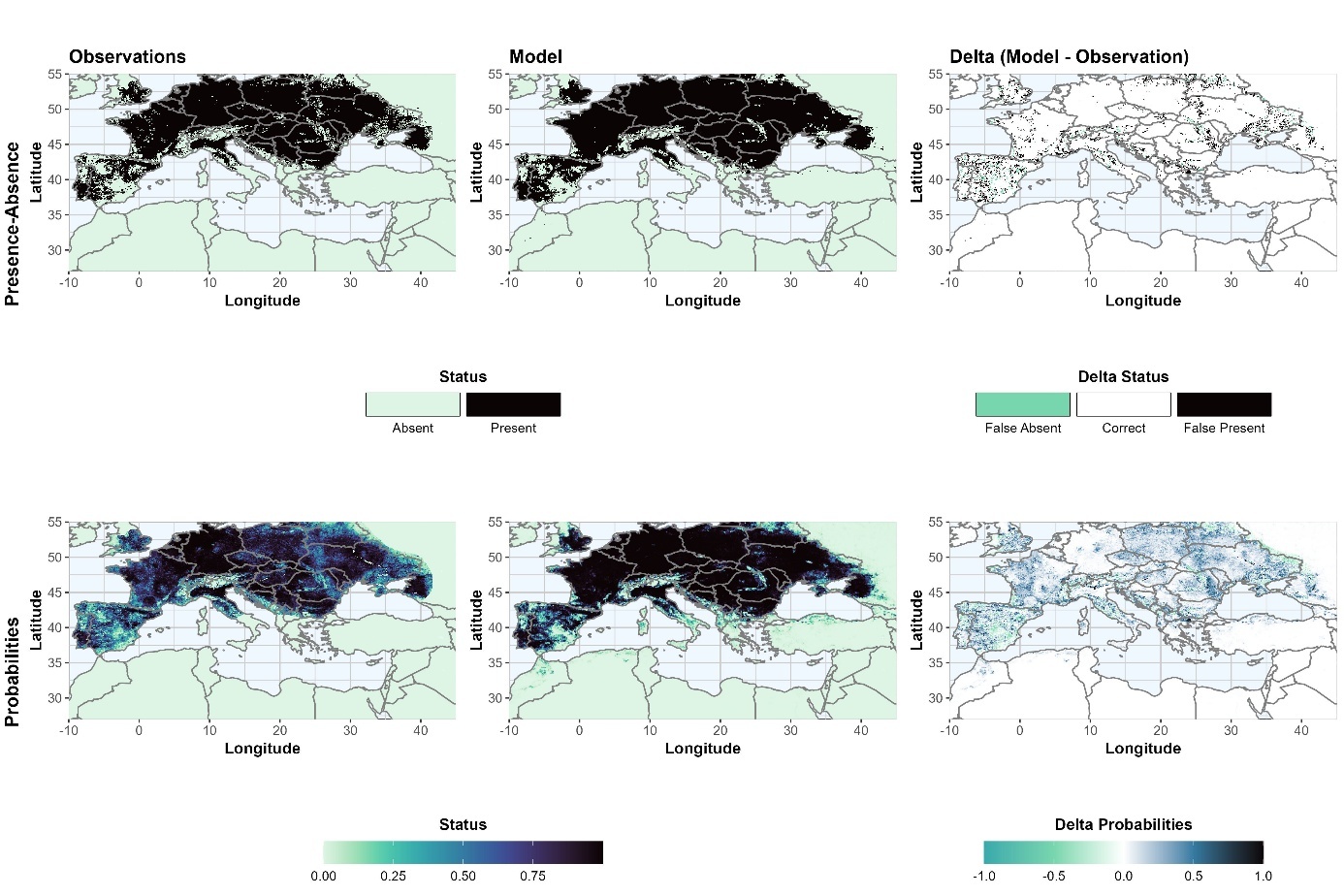


*Figure S2.3.1: Observations, presence-absence and probabilities of the best model setup and the differences for the year 2010 of* ***An. atroparvus****.*

S2.3.2 *An. labranchiae*


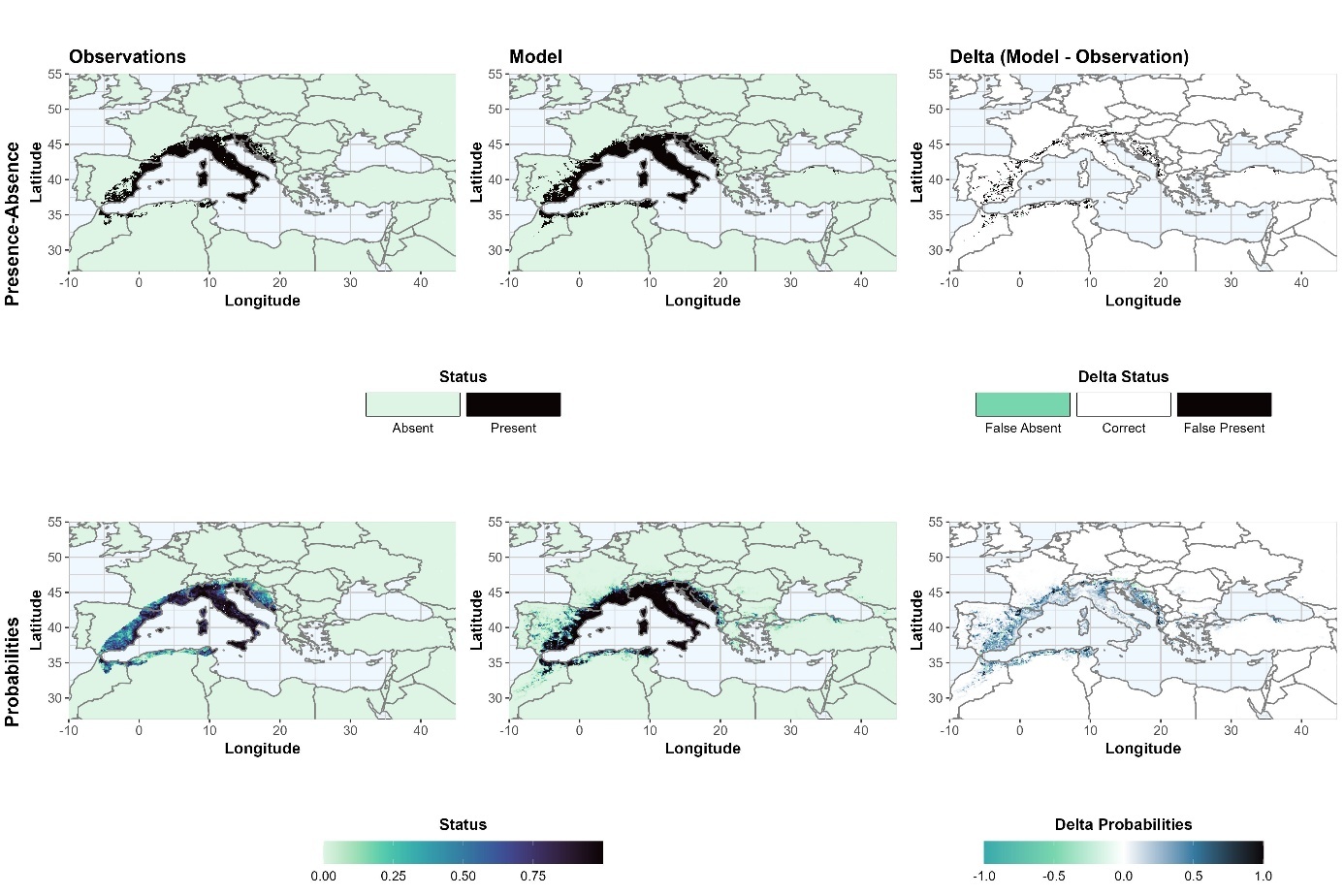


*Figure S2.3.2: Observations, presence-absence and probabilities of the best model setup and the differences for the year 2010 of* ***An. labranchiae****.*

S2.3.3 *An. messeae*

**
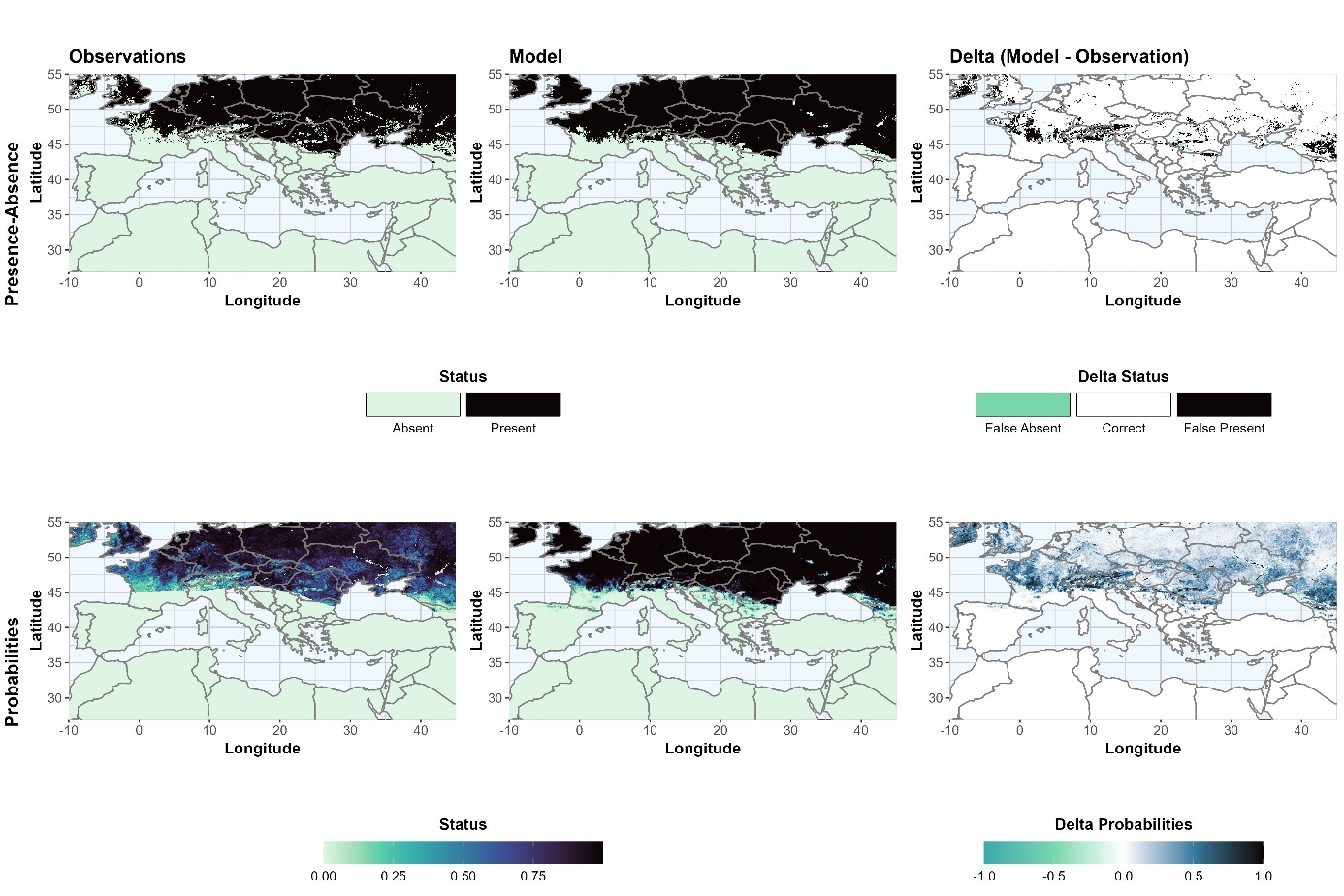
**

*Figure S2.3.3: Observations, presence-absence and probabilities of the best model setup and the differences for the year 2010 of* ***An. messeae****.*

S2.3.4 *An. sacharovi*

**
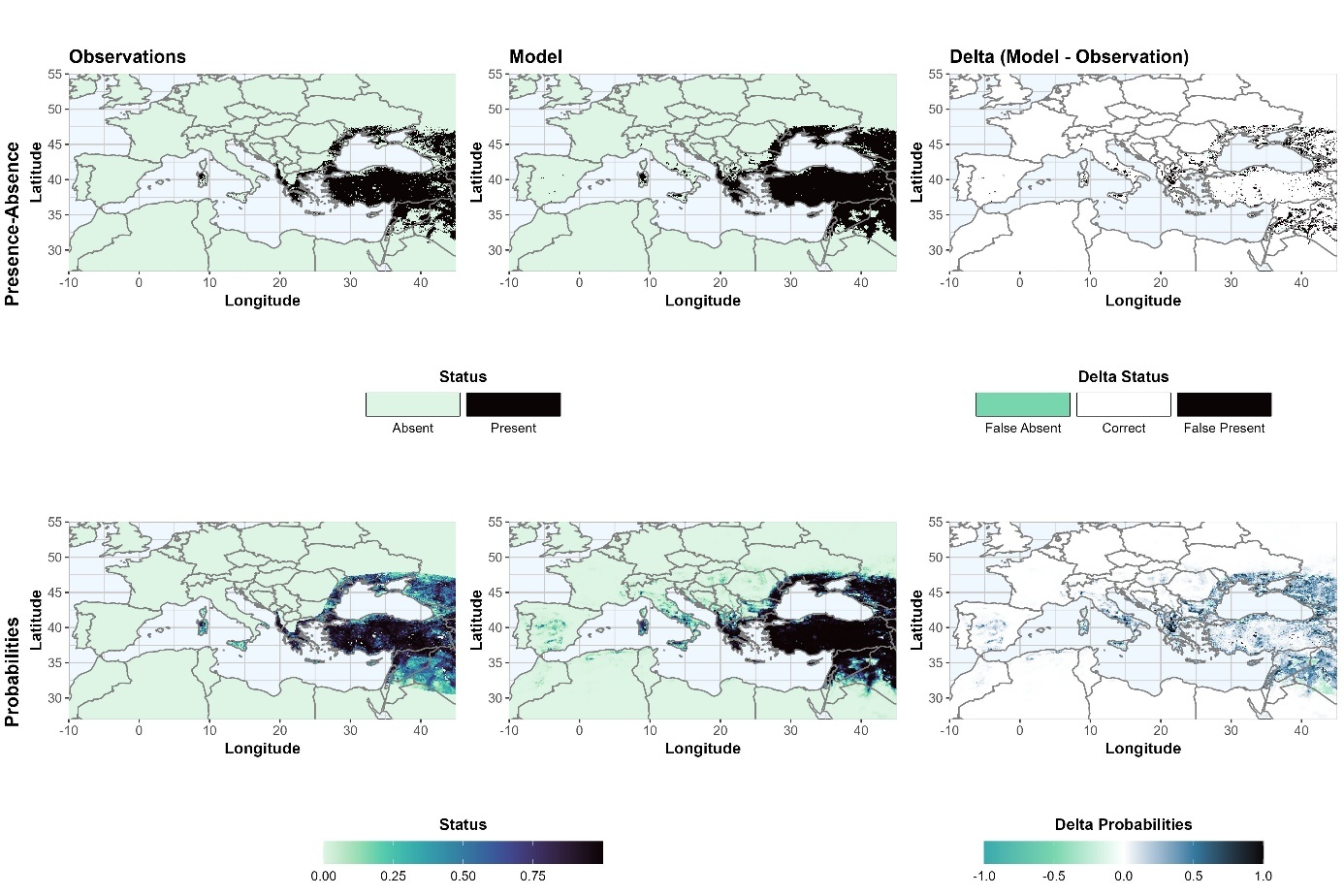
**

*Figure S2.3.4: Observations, presence-absence and probabilities of the best model setup and the differences for the year 2010 of* ***An. sacharovi****.*

S2.3.5 *An. sergentii*

**
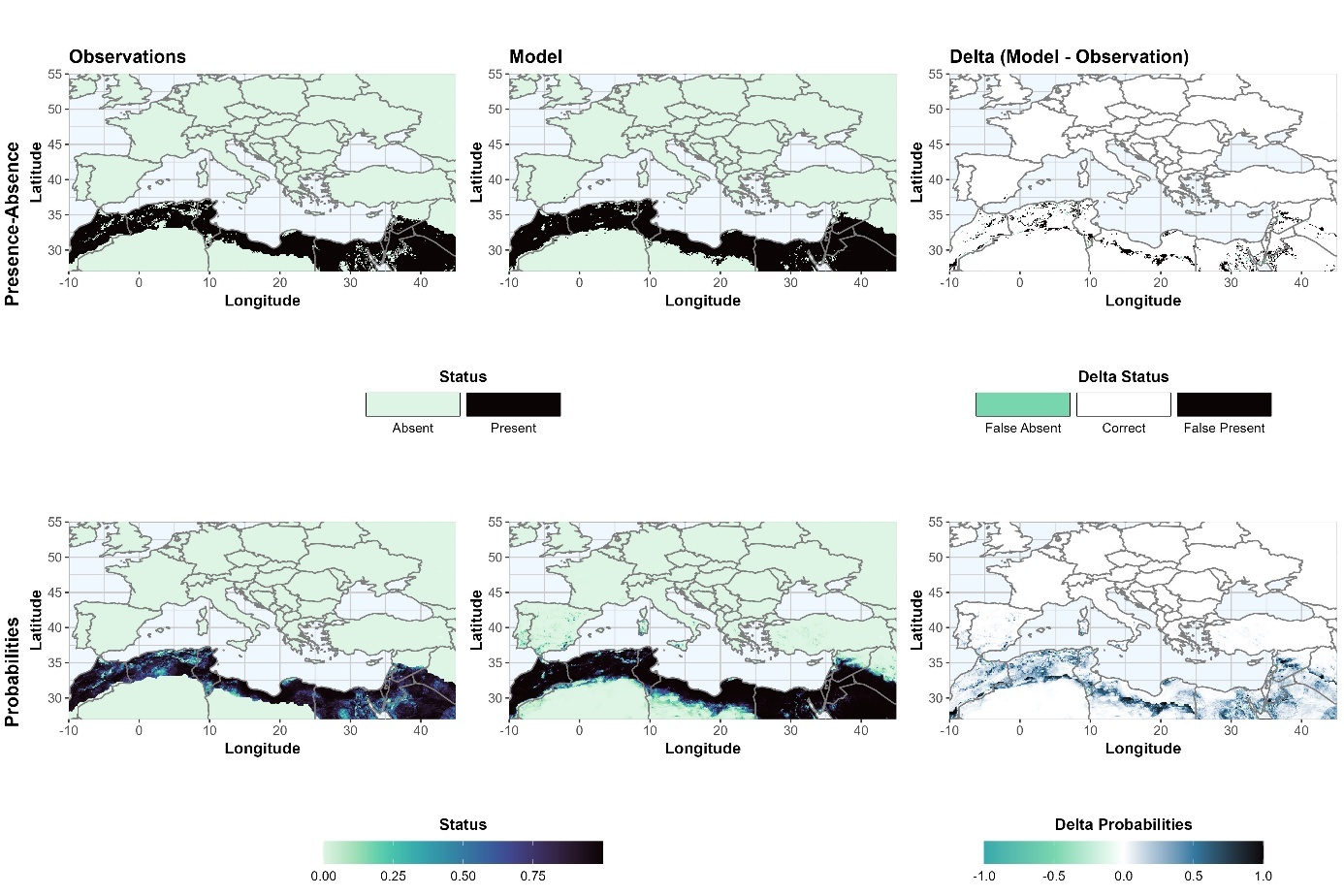
**

*Figure S2.3.5: Observations, presence-absence and probabilities of the best model setup and the differences for the year 2010 of* ***An. sergentii****.*

S2.3.6 *An. superpictus*


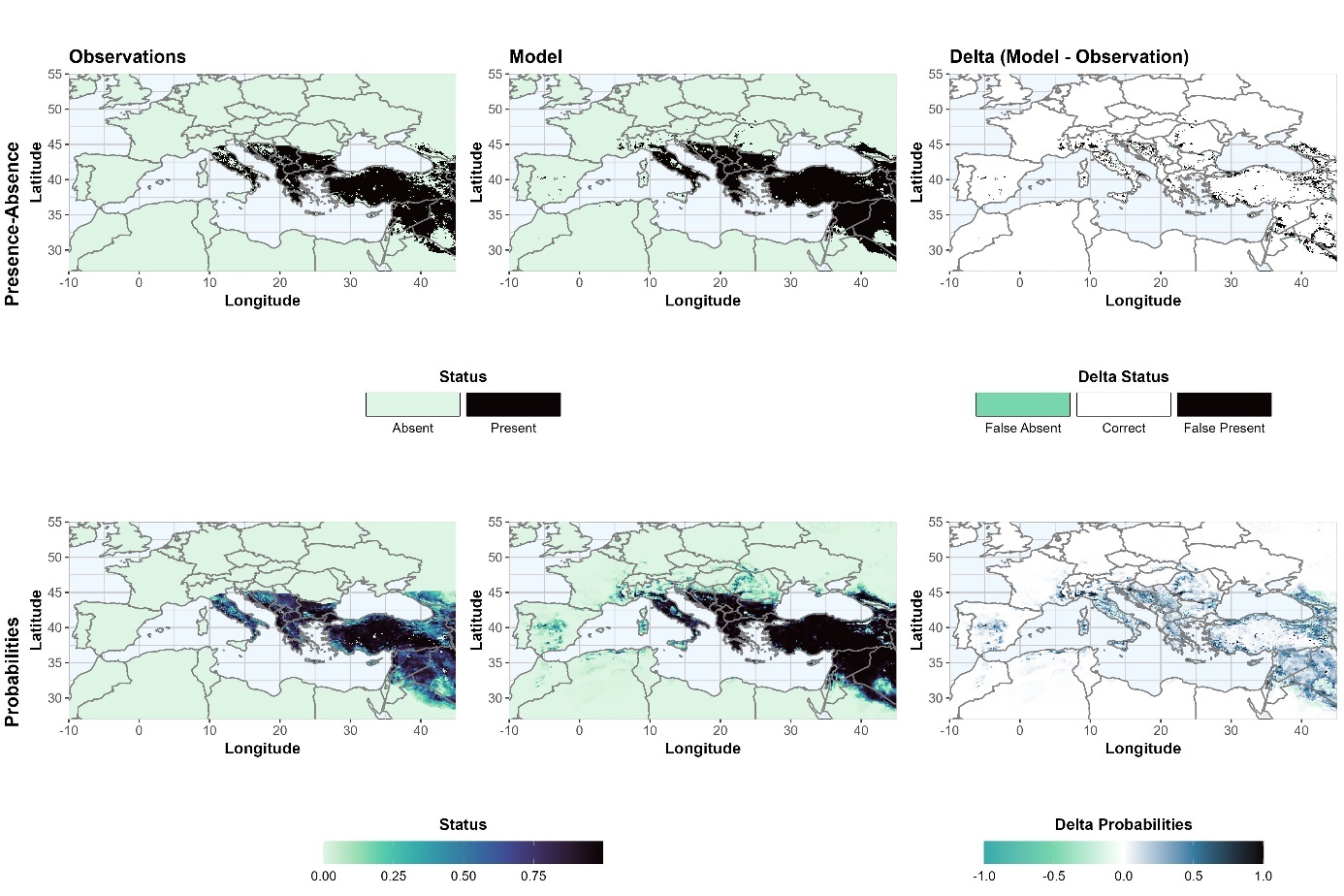


*Figure S2.3.6: Observations, presence-absence and probabilities of the best model setup and the differences for the year 2010 of* ***An. superpictus****.*

**S3 Reasons for Change**

S3.1 *An. atroparvus*

*Table S3.1: Results per category of change for each model setup of* ***An. atroparvus*** *between 2000 and 2020. In addition to the categories of change, the presence area as a fraction of the land grid boxes of the study area is given for 2000 (P.2000) and 2020 (P.2020), changes as a fraction of the presence area of 2000 for the total changes (CHANGE), changes due to climate (CHANGE.CLIM) and changes due to land use (CHANGE.LU).*

| **Species** | **Buffered** | **Sampled** | **Selection** | **P.2000** | **P.2020** | **CHANGE** | **PCC** | **ACC** | **PLUC** | **ALUC** | **RPLUC** | **APLUC** | **CHANGE.CLIM** | **CHANGE.LU** |
| --- | --- | --- | --- | --- | --- | --- | --- | --- | --- | --- | --- | --- | --- | --- |
| ***atroparvus*** |  |  | **I** | 35.1 | 37.1 | 5.9 | 9.8 | 3.9 | 1.3 | 1.3 | 1.0 | 0.6 | 5.9 | 0.0 |
|  |  |  | **II** | 35.5 | 37.5 | 5.6 | 9.9 | 4.1 | 1.2 | 1.4 | 0.9 | 0.6 | 5.8 | -0.2 |
|  |  |  | **III** | 35.5 | 37.9 | 6.7 | 10.1 | 3.5 | 1.3 | 1.2 | 0.9 | 0.7 | 6.6 | 0.1 |
|  |  |  | **IV** | 34.7 | 37.1 | 7.0 | 10.6 | 3.6 | 1.5 | 1.5 | 0.9 | 0.8 | 7.0 | 0.0 |
|  | **x** |  | **I** | 38.6 | 40.1 | 3.9 | 7.1 | 3.0 | 0.6 | 0.8 | 0.7 | 0.4 | 4.1 | -0.2 |
|  | **x** |  | **II** | 39.3 | 40.4 | 2.8 | 6.7 | 3.6 | 0.5 | 0.8 | 0.7 | 0.3 | 3.1 | -0.3 |
|  | **x** |  | **III** | 39.5 | 41.0 | 3.8 | 6.8 | 2.8 | 0.5 | 0.7 | 0.6 | 0.2 | 4.0 | -0.2 |
|  | **x** |  | **IV** | 39.4 | 41.0 | 4.1 | 6.9 | 2.6 | 0.6 | 0.8 | 0.7 | 0.3 | 4.3 | -0.2 |
|  |  | **x** | **I** | 34.9 | 37.2 | 6.6 | 10.2 | 3.8 | 1.3 | 1.1 | 0.7 | 0.6 | 6.4 | 0.2 |
|  |  | **x** | **II** | 35.5 | 37.7 | 5.9 | 9.5 | 3.5 | 1.3 | 1.4 | 0.8 | 0.5 | 6.0 | -0.1 |
|  |  | **x** | **III** | 35.0 | 37.1 | 6.2 | 10.4 | 4.3 | 1.4 | 1.3 | 0.8 | 0.5 | 6.1 | 0.1 |
|  |  | **x** | **IV** | 36.2 | 37.9 | 4.8 | 8.2 | 3.1 | 1.3 | 1.6 | 0.7 | 0.6 | 5.1 | -0.3 |
|  | **x** | **x** | **I** | 39.2 | 40.1 | 2.5 | 6.1 | 3.2 | 0.4 | 0.8 | 0.6 | 0.3 | 2.9 | -0.4 |
|  | **x** | **x** | **II** | 40.1 | 41.0 | 2.4 | 5.4 | 2.7 | 0.4 | 0.7 | 0.5 | 0.2 | 2.7 | -0.3 |
|  | **x** | **x** | **III** | 39.3 | 40.3 | 2.6 | 5.6 | 2.8 | 0.6 | 0.8 | 0.6 | 0.2 | 2.8 | -0.2 |
|  | **x** | **x** | **IV** | 39.1 | 40.3 | 3.2 | 6.3 | 2.8 | 0.6 | 0.9 | 0.6 | 0.3 | 3.5 | -0.3 |

S3.2 *An. labranchiae*

*Table S3.2: Results per category of change for each model setup of* ***An. labranchiae*** *between 2000 and 2020. In addition to the categories of change, the presence area as a fraction of the land grid boxes of the study area is given for 2000 (P.2000) and 2020 (P.2020), changes as a fraction of the presence area of 2000 for the total changes (CHANGE), changes due to climate (CHANGE.CLIM) and changes due to land use (CHANGE.LU).*

| **Species** | **Buffered** | **Sampled** | **Selection** | **P.2000** | **P.2020** | **CHANGE** | **PCC** | **ACC** | **PLUC** | **ALUC** | **RPLUC** | **APLUC** | **CHANGE.CLIM** | **CHANGE.LU** |
| --- | --- | --- | --- | --- | --- | --- | --- | --- | --- | --- | --- | --- | --- | --- |
| ***labranchiae*** |  |  | **I** | 7.5 | 7.6 | 0.5 | 8.4 | 8.4 | 2.1 | 1.6 | 1.4 | 1.4 | 0.0 | 0.5 |
|  |  |  | **II** | 8.1 | 8.1 | 0.5 | 8.4 | 8.3 | 2.3 | 1.9 | 1.3 | 2.0 | 0.1 | 0.4 |
|  |  |  | **III** | 7.8 | 7.9 | 0.9 | 8.4 | 8.3 | 3.1 | 2.3 | 1.4 | 1.8 | 0.1 | 0.8 |
|  |  |  | **IV** | 8.5 | 8.3 | -2.0 | 7.9 | 10.1 | 3.1 | 2.9 | 2.1 | 2.4 | -2.2 | 0.2 |
|  | **x** |  | **I** | 8.0 | 7.9 | -1.2 | 6.7 | 7.9 | 1.3 | 1.3 | 1.0 | 1.2 | -1.2 | 0.0 |
|  | **x** |  | **II** | 7.7 | 7.6 | -0.3 | 7.3 | 7.9 | 1.3 | 1.0 | 1.0 | 0.8 | -0.6 | 0.3 |
|  | **x** |  | **III** | 8.0 | 8.0 | 0.4 | 7.5 | 7.7 | 1.6 | 1.0 | 0.8 | 0.9 | -0.2 | 0.6 |
|  | **x** |  | **IV** | 8.4 | 8.3 | -1.3 | 7.5 | 9.1 | 1.9 | 1.6 | 1.3 | 1.3 | -1.6 | 0.3 |
|  |  | **x** | **I** | 8.0 | 7.9 | -0.9 | 8.3 | 10.1 | 2.8 | 1.9 | 1.5 | 1.7 | -1.8 | 0.9 |
|  |  | **x** | **II** | 8.3 | 8.1 | -2.5 | 8.0 | 10.8 | 2.2 | 1.9 | 1.4 | 2.0 | -2.8 | 0.3 |
|  |  | **x** | **III** | 7.9 | 7.8 | -1.2 | 8.8 | 10.5 | 2.5 | 2.0 | 1.6 | 1.8 | -1.7 | 0.5 |
|  |  | **x** | **IV** | 8.5 | 8.2 | -2.7 | 8.3 | 11.9 | 3.7 | 2.8 | 2.3 | 2.1 | -3.6 | 0.9 |
|  | **x** | **x** | **I** | 8.2 | 8.2 | -0.7 | 7.1 | 8.2 | 1.5 | 1.1 | 0.9 | 1.0 | -1.1 | 0.4 |
|  | **x** | **x** | **II** | 7.9 | 7.8 | -0.8 | 7.0 | 7.9 | 1.2 | 1.1 | 1.0 | 0.8 | -0.9 | 0.1 |
|  | **x** | **x** | **III** | 7.9 | 7.8 | -0.7 | 7.7 | 8.3 | 1.3 | 1.4 | 0.9 | 0.8 | -0.6 | -0.1 |
|  | **x** | **x** | **IV** | 8.3 | 8.1 | -2.2 | 7.0 | 9.3 | 1.6 | 1.5 | 1.5 | 1.2 | -2.3 | 0.1 |

S3.3 *An. messeae*

*Table S3.3: Results per category of change for each model setup of* ***An. messeae*** *between 2000 and 2020. In addition to the categories of change, the presence area as a fraction of the land grid boxes of the study area is given for 2000 (P.2000) and 2020 (P.2020), changes as a fraction of the presence area of 2000 for the total changes (CHANGE), changes due to climate (CHANGE.CLIM) and changes due to land use (CHANGE.LU).*

| **Species** | **Buffered** | **Sampled** | **Selection** | **P.2000** | **P.2020** | **CHANGE** | **PCC** | **ACC** | **PLUC** | **ALUC** | **RPLUC** | **APLUC** | **CHANGE.CLIM** | **CHANGE.LU** |
| --- | --- | --- | --- | --- | --- | --- | --- | --- | --- | --- | --- | --- | --- | --- |
| ***messeae*** |  |  | **I** | 36.5 | 35.5 | -2.7 | 2.9 | 5.7 | 0.6 | 0.5 | 0.7 | 0.2 | -2.8 | 0.1 |
|  |  |  | **II** | 37.0 | 36.0 | -2.8 | 2.5 | 5.1 | 0.5 | 0.7 | 0.7 | 0.2 | -2.6 | -0.2 |
|  |  |  | **III** | 37.1 | 35.9 | -3.4 | 2.2 | 5.4 | 0.4 | 0.6 | 0.6 | 0.2 | -3.2 | -0.2 |
|  |  |  | **IV** | 37.2 | 36.4 | -2.2 | 2.0 | 4.2 | 0.7 | 0.7 | 0.8 | 0.3 | -2.2 | 0.0 |
|  | **x** |  | **I** | 39.0 | 37.1 | -5.0 | 0.8 | 5.6 | 0.2 | 0.4 | 0.5 | 0.1 | -4.8 | -0.2 |
|  | **x** |  | **II** | 39.4 | 37.3 | -5.2 | 0.8 | 5.8 | 0.2 | 0.4 | 0.5 | 0.1 | -5.0 | -0.2 |
|  | **x** |  | **III** | 39.2 | 37.3 | -5.0 | 0.8 | 5.5 | 0.2 | 0.5 | 0.5 | 0.1 | -4.7 | -0.3 |
|  | **x** |  | **IV** | 39.1 | 37.6 | -3.8 | 0.8 | 4.3 | 0.3 | 0.6 | 0.6 | 0.1 | -3.5 | -0.3 |
|  |  | **x** | **I** | 36.6 | 35.3 | -3.4 | 2.6 | 5.9 | 0.5 | 0.6 | 0.5 | 0.3 | -3.3 | -0.1 |
|  |  | **x** | **II** | 36.6 | 35.3 | -3.6 | 2.4 | 5.9 | 0.5 | 0.6 | 0.6 | 0.3 | -3.5 | -0.1 |
|  |  | **x** | **III** | 37.0 | 36.1 | -2.5 | 2.5 | 5.1 | 0.6 | 0.5 | 0.4 | 0.3 | -2.6 | 0.1 |
|  |  | **x** | **IV** | 37.1 | 36.1 | -2.5 | 2.0 | 4.5 | 0.6 | 0.6 | 0.7 | 0.3 | -2.5 | 0.0 |
|  | **x** | **x** | **I** | 39.0 | 37.0 | -5.2 | 0.8 | 5.8 | 0.3 | 0.5 | 0.6 | 0.1 | -5.0 | -0.2 |
|  | **x** | **x** | **II** | 39.2 | 37.0 | -5.4 | 0.6 | 5.7 | 0.2 | 0.5 | 0.6 | 0.1 | -5.1 | -0.3 |
|  | **x** | **x** | **III** | 38.8 | 36.8 | -5.2 | 0.7 | 5.7 | 0.3 | 0.5 | 0.6 | 0.1 | -5.0 | -0.2 |
|  | **x** | **x** | **IV** | 38.3 | 36.8 | -4.1 | 0.9 | 4.8 | 0.4 | 0.6 | 0.7 | 0.1 | -3.9 | -0.2 |

S3.4 *An. sacharovi*

*Table S3.4: Results per category of change for each model setup of* ***An. sacharovi*** *between 2000 and 2020. In addition to the categories of change, the presence area as a fraction of the land grid boxes of the study area is given for 2000 (P.2000) and 2020 (P.2020), changes as a fraction of the presence area of 2000 for the total changes (CHANGE), changes due to climate (CHANGE.CLIM) and changes due to land use (CHANGE.LU).*

| **Species** | **Buffered** | **Sampled** | **Selection** | **P.2000** | **P.2020** | **CHANGE** | **PCC** | **ACC** | **PLUC** | **ALUC** | **RPLUC** | **APLUC** | **CHANGE.CLIM** | **CHANGE.LU** |
| --- | --- | --- | --- | --- | --- | --- | --- | --- | --- | --- | --- | --- | --- | --- |
| ***sacharovi*** |  |  | **I** | 15.8 | 16.8 | 6.5 | 11.1 | 5.5 | 2.0 | 1.1 | 0.8 | 0.9 | 5.6 | 0.9 |
|  |  |  | **II** | 16.5 | 17.9 | 8.3 | 11.6 | 4.5 | 1.9 | 0.7 | 0.7 | 0.9 | 7.1 | 1.2 |
|  |  |  | **III** | 16.0 | 17.3 | 8.2 | 11.7 | 4.9 | 2.3 | 0.9 | 0.9 | 0.9 | 6.8 | 1.4 |
|  |  |  | **IV** | 16.1 | 17.6 | 9.4 | 13.0 | 4.9 | 2.3 | 1.0 | 0.9 | 1.0 | 8.1 | 1.3 |
|  | **x** |  | **I** | 17.2 | 18.1 | 5.3 | 8.4 | 4.2 | 1.6 | 0.5 | 0.7 | 0.4 | 4.2 | 1.1 |
|  | **x** |  | **II** | 17.1 | 18.1 | 5.7 | 8.5 | 3.9 | 1.6 | 0.5 | 0.8 | 0.5 | 4.6 | 1.1 |
|  | **x** |  | **III** | 17.0 | 18.2 | 6.9 | 8.8 | 3.1 | 1.8 | 0.6 | 0.6 | 0.6 | 5.7 | 1.2 |
|  | **x** |  | **IV** | 17.4 | 18.4 | 5.9 | 7.1 | 3.0 | 2.5 | 0.7 | 0.7 | 0.7 | 4.1 | 1.8 |
|  |  | **x** | **I** | 16.6 | 17.8 | 7.6 | 11.9 | 5.5 | 2.1 | 0.9 | 0.7 | 1.0 | 6.4 | 1.2 |
|  |  | **x** | **II** | 16.3 | 17.3 | 6.6 | 11.5 | 5.6 | 1.6 | 0.9 | 0.7 | 0.8 | 5.9 | 0.7 |
|  |  | **x** | **III** | 16.3 | 17.4 | 6.4 | 11.1 | 5.6 | 1.8 | 0.9 | 0.7 | 0.9 | 5.5 | 0.9 |
|  |  | **x** | **IV** | 16.0 | 17.6 | 10.3 | 13.3 | 4.3 | 2.4 | 1.1 | 0.7 | 2.0 | 9.0 | 1.3 |
|  | **x** | **x** | **I** | 17.4 | 18.1 | 3.7 | 7.2 | 4.2 | 1.4 | 0.7 | 0.6 | 0.5 | 3.0 | 0.7 |
|  | **x** | **x** | **II** | 17.2 | 17.9 | 4.1 | 7.9 | 4.3 | 1.3 | 0.8 | 0.6 | 0.4 | 3.6 | 0.5 |
|  | **x** | **x** | **III** | 17.0 | 17.8 | 4.6 | 8.1 | 3.9 | 1.0 | 0.6 | 0.5 | 0.4 | 4.2 | 0.4 |
|  | **x** | **x** | **IV** | 17.3 | 18.1 | 5.2 | 8.3 | 4.0 | 1.5 | 0.6 | 0.7 | 0.6 | 4.3 | 0.9 |

S3.5 *An. sergentii*

*Table S3.5: Results per category of change for each model setup of* ***An. sergentii*** *between 2000 and 2020. In addition to the categories of change, the presence area as a fraction of the land grid boxes of the study area is given for 2000 (P.2000) and 2020 (P.2020), changes as a fraction of the presence area of 2000 for the total changes (CHANGE), changes due to climate (CHANGE.CLIM) and changes due to land use (CHANGE.LU).*

| **Species** | **Buffered** | **Sampled** | **Selection** | **P.2000** | **P.2020** | **CHANGE** | **PCC** | **ACC** | **PLUC** | **ALUC** | **RPLUC** | **APLUC** | **CHANGE.CLIM** | **CHANGE.LU** |
| --- | --- | --- | --- | --- | --- | --- | --- | --- | --- | --- | --- | --- | --- | --- |
| ***sergentii*** |  |  | **I** | 20.7 | 19.5 | -5.6 | 1.7 | 7.1 | 0.2 | 0.4 | 0.1 | 0.3 | -5.4 | -0.2 |
|  |  |  | **II** | 20.5 | 19.4 | -5.7 | 1.8 | 7.1 | 0.2 | 0.6 | 0.1 | 0.3 | -5.3 | -0.4 |
|  |  |  | **III** | 20.5 | 19.2 | -6.2 | 1.7 | 7.4 | 0.2 | 0.7 | 0.1 | 0.3 | -5.7 | -0.5 |
|  |  |  | **IV** | 20.0 | 19.4 | -3.0 | 2.5 | 5.1 | 0.3 | 0.7 | 0.1 | 0.3 | -2.6 | -0.4 |
|  | **x** |  | **I** | 20.7 | 19.4 | -6.0 | 1.1 | 6.8 | 0.1 | 0.4 | 0.1 | 0.2 | -5.7 | -0.3 |
|  | **x** |  | **II** | 20.5 | 19.3 | -5.7 | 1.2 | 6.6 | 0.1 | 0.4 | 0.0 | 0.1 | -5.4 | -0.3 |
|  | **x** |  | **III** | 20.6 | 19.4 | -5.9 | 1.2 | 6.8 | 0.1 | 0.4 | 0.1 | 0.1 | -5.6 | -0.3 |
|  | **x** |  | **IV** | 19.9 | 19.3 | -3.1 | 1.8 | 4.6 | 0.1 | 0.4 | 0.1 | 0.2 | -2.8 | -0.3 |
|  |  | **x** | **I** | 20.5 | 19.4 | -5.3 | 1.7 | 6.6 | 0.1 | 0.5 | 0.1 | 0.2 | -4.9 | -0.4 |
|  |  | **x** | **II** | 20.6 | 19.4 | -5.6 | 1.8 | 6.9 | 0.2 | 0.7 | 0.1 | 0.2 | -5.1 | -0.5 |
|  |  | **x** | **III** | 20.8 | 19.6 | -5.6 | 1.8 | 6.7 | 0.1 | 0.8 | 0.1 | 0.3 | -4.9 | -0.7 |
|  |  | **x** | **IV** | 20.2 | 19.4 | -3.7 | 2.4 | 5.5 | 0.2 | 0.8 | 0.1 | 0.3 | -3.1 | -0.6 |
|  | **x** | **x** | **I** | 21.2 | 19.8 | -6.4 | 1.5 | 7.5 | 0.1 | 0.5 | 0.0 | 0.1 | -6.0 | -0.4 |
|  | **x** | **x** | **II** | 20.9 | 19.7 | -5.9 | 1.6 | 7.1 | 0.0 | 0.4 | 0.0 | 0.2 | -5.5 | -0.4 |
|  | **x** | **x** | **III** | 21.1 | 19.7 | -6.3 | 1.2 | 7.2 | 0.1 | 0.4 | 0.0 | 0.3 | -6.0 | -0.3 |
|  | **x** | **x** | **IV** | 20.4 | 19.7 | -3.4 | 2.0 | 5.1 | 0.1 | 0.4 | 0.0 | 0.3 | -3.1 | -0.3 |

S3.6 *An. superpictus*

*Table S3.6: Results per category of change for each model setup of* ***An. superpictus*** *between 2000 and 2020. In addition to the categories of change, the presence area as a fraction of the land grid boxes of the study area is given for 2000 (P.2000) and 2020 (P.2020), changes as a fraction of the presence area of 2000 for the total changes (CHANGE), changes due to climate (CHANGE.CLIM) and changes due to land use (CHANGE.LU).*

| **Species** | **Buffered** | **Sampled** | **Selection** | **P.2000** | **P.2020** | **CHANGE** | **PCC** | **ACC** | **PLUC** | **ALUC** | **RPLUC** | **APLUC** | **CHANGE.CLIM** | **CHANGE.LU** |
| --- | --- | --- | --- | --- | --- | --- | --- | --- | --- | --- | --- | --- | --- | --- |
| ***superpictus*** |  |  | **I** | 18.2 | 19.0 | 4.1 | 7.1 | 3.7 | 1.1 | 0.4 | 0.2 | 0.7 | 3.4 | 0.7 |
|  |  |  | **II** | 17.8 | 18.6 | 4.6 | 7.4 | 3.6 | 1.2 | 0.4 | 0.2 | 0.5 | 3.8 | 0.8 |
|  |  |  | **III** | 17.8 | 18.6 | 4.4 | 7.2 | 3.5 | 1.1 | 0.4 | 0.3 | 0.6 | 3.7 | 0.7 |
|  |  |  | **IV** | 18.0 | 18.8 | 4.0 | 7.0 | 3.8 | 1.5 | 0.7 | 0.3 | 1.0 | 3.2 | 0.8 |
|  | **x** |  | **I** | 21.3 | 20.8 | -2.4 | 4.9 | 1.2 | 1.0 | 7.1 | 0.2 | 0.5 | 3.7 | -6.1 |
|  | **x** |  | **II** | 19.9 | 20.8 | 4.6 | 5.2 | 1.1 | 0.8 | 0.3 | 0.1 | 0.4 | 4.1 | 0.5 |
|  | **x** |  | **III** | 19.7 | 20.5 | 4.4 | 5.3 | 1.3 | 0.7 | 0.3 | 0.2 | 0.5 | 4.0 | 0.4 |
|  | **x** |  | **IV** | 20.6 | 20.6 | 0.0 | 5.0 | 1.5 | 1.9 | 5.4 | 0.2 | 0.8 | 3.5 | -3.5 |
|  |  | **x** | **I** | 17.6 | 18.5 | 5.3 | 8.0 | 3.5 | 1.3 | 0.5 | 0.3 | 0.7 | 4.5 | 0.8 |
|  |  | **x** | **II** | 17.5 | 18.2 | 4.2 | 7.4 | 4.1 | 1.4 | 0.5 | 0.4 | 0.6 | 3.3 | 0.9 |
|  |  | **x** | **III** | 17.6 | 18.3 | 4.1 | 6.8 | 3.8 | 1.6 | 0.5 | 0.3 | 0.6 | 3.0 | 1.1 |
|  |  | **x** | **IV** | 17.4 | 18.4 | 5.6 | 8.1 | 3.3 | 1.7 | 0.9 | 0.5 | 0.8 | 4.8 | 0.8 |
|  | **x** | **x** | **I** | 19.3 | 20.0 | 3.6 | 5.2 | 1.9 | 0.6 | 0.3 | 0.1 | 0.3 | 3.3 | 0.3 |
|  | **x** | **x** | **II** | 19.2 | 19.9 | 3.8 | 5.2 | 1.7 | 0.5 | 0.2 | 0.2 | 0.4 | 3.5 | 0.3 |
|  | **x** | **x** | **III** | 19.1 | 19.8 | 3.4 | 5.0 | 1.9 | 0.6 | 0.3 | 0.2 | 0.3 | 3.1 | 0.3 |
|  | **x** | **x** | **IV** | 19.1 | 19.7 | 2.9 | 4.5 | 1.8 | 0.6 | 0.4 | 0.3 | 0.4 | 2.7 | 0.2 |

**S4 Most important predictors**


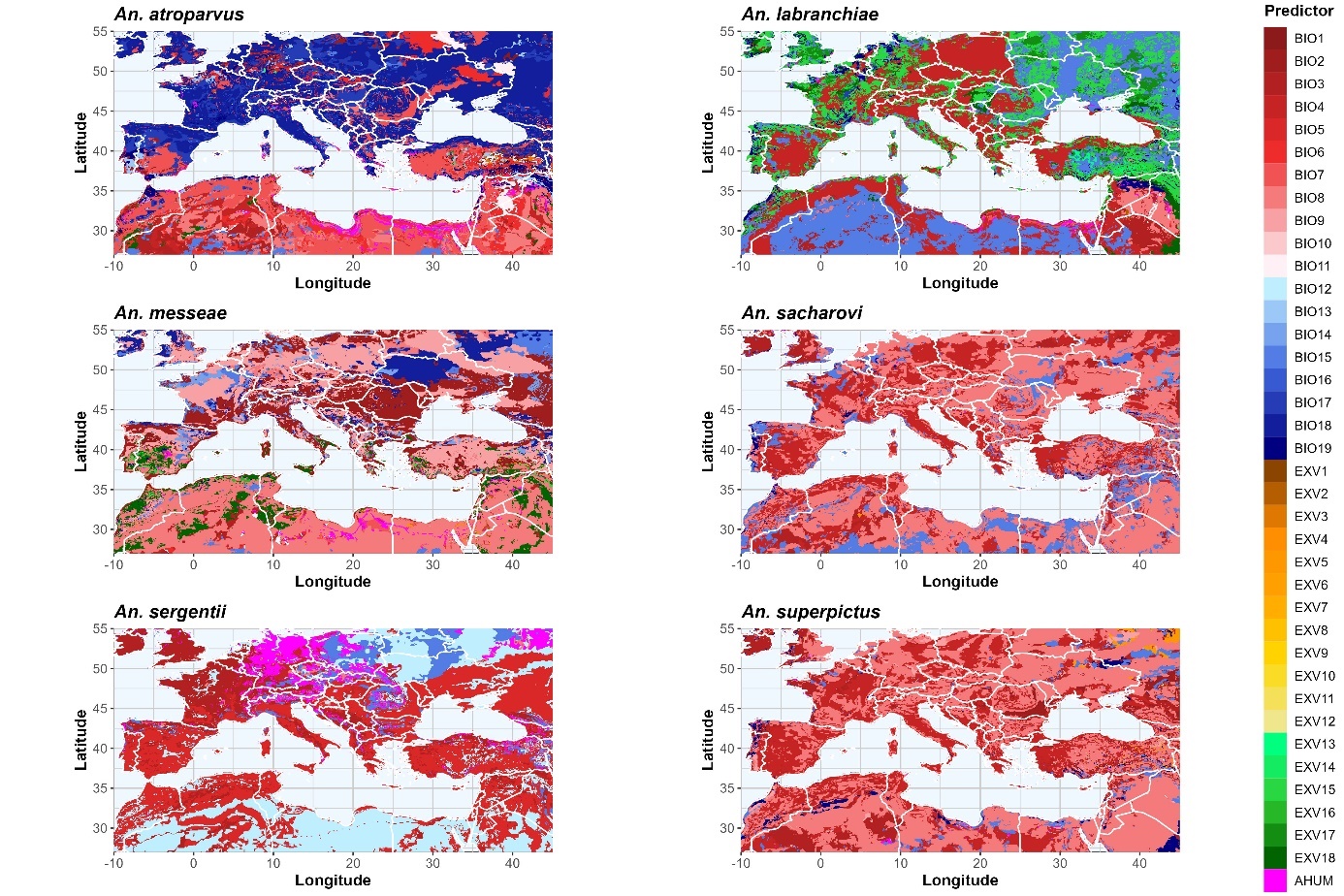


*Figure S4.1: Most important climatic predictors.*


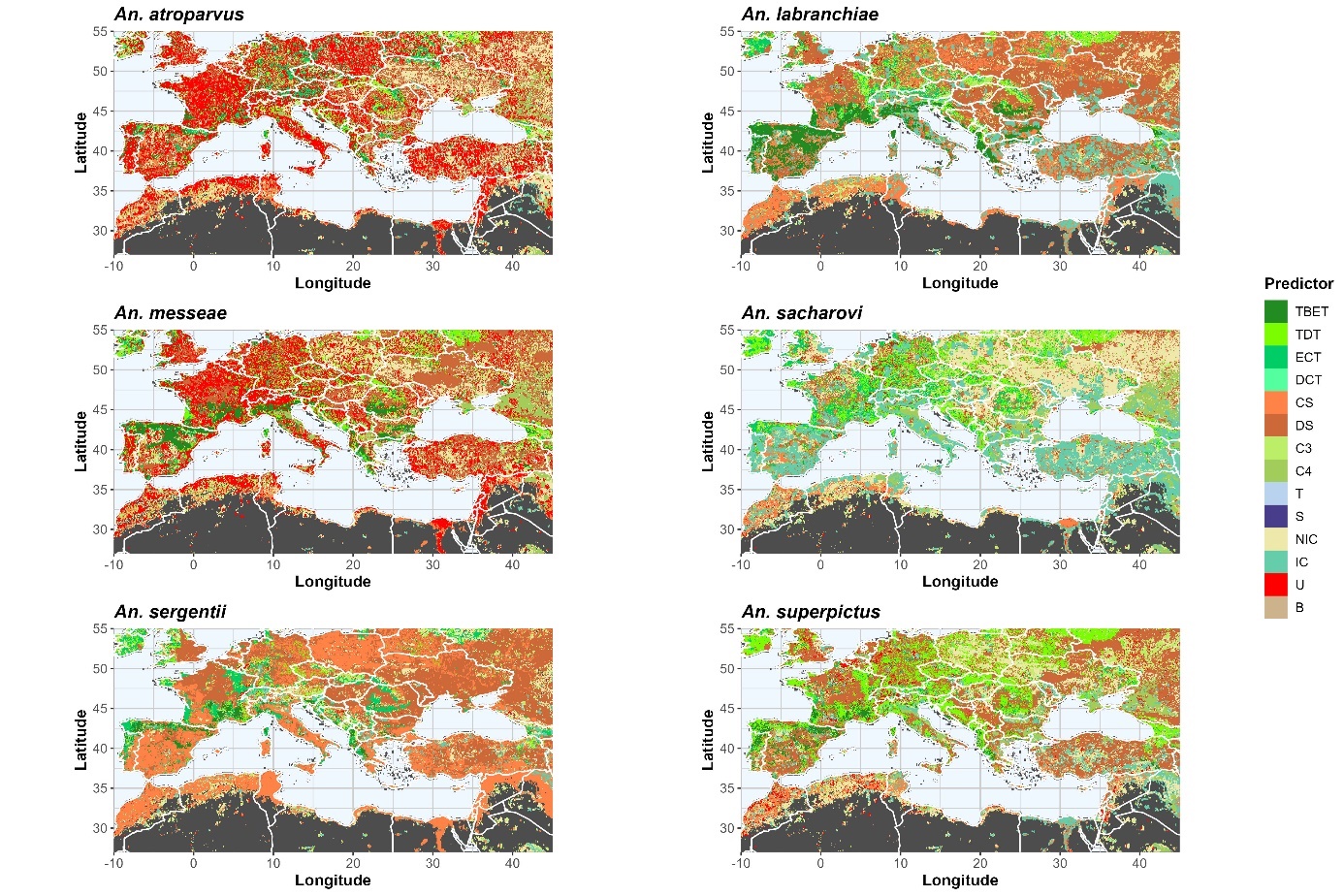


*Figure S4.2: Most important land-use predictors.*

S4.1 *An. atroparvus*


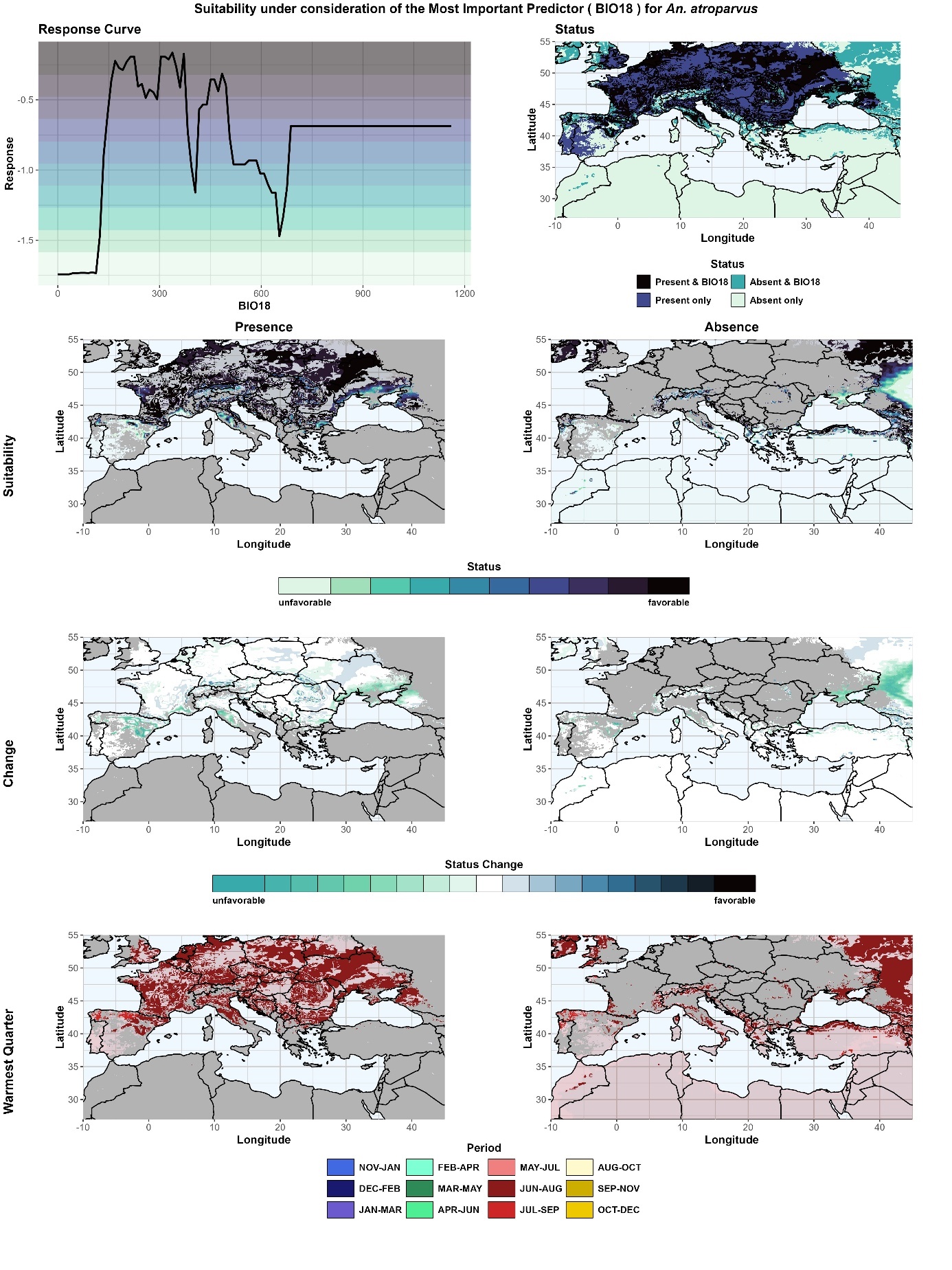


*Figure S4.1.1: Suitability under consideration of the most important climatic predictor for* ***An. atroparvus****. The response curves of the MIP and the status is presented at the top. Below, suitability maps are given for the presence (left) and absence areas (right), changes in suitability (3^rd^ row) and the reference quarter representing the warmest quarter* *obtained from ERA5-Land (bottom).*


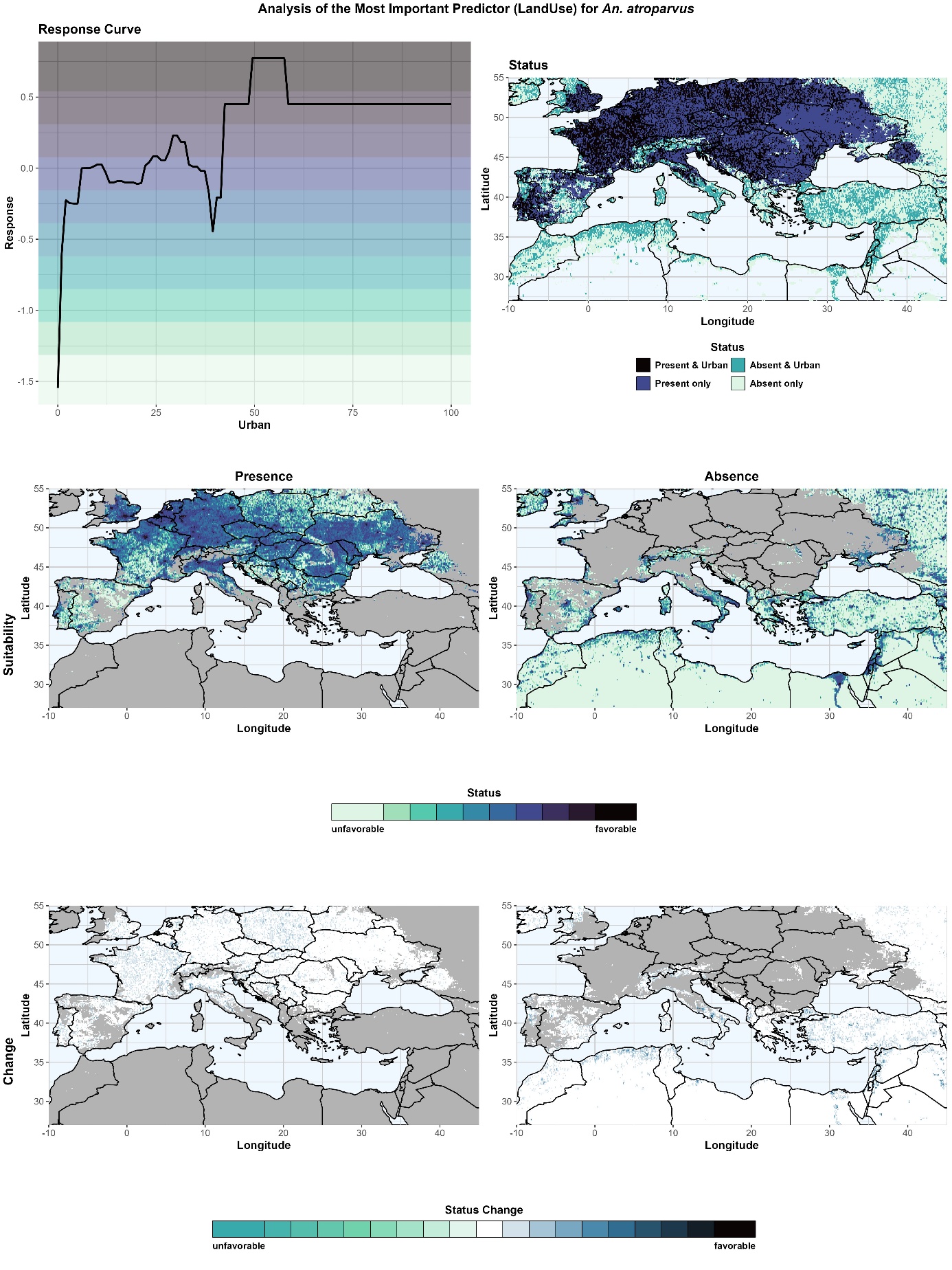


*Figure S4.1.2: Suitability under consideration of the most important land-use predictor for* ***An. atroparvus****. The response curves of the MIP and the status is presented at the top. Below, suitability maps are given for the presence (left) and absence areas (right), and changes in suitability at the bottom.*

S4.2 *An. labranchiae*


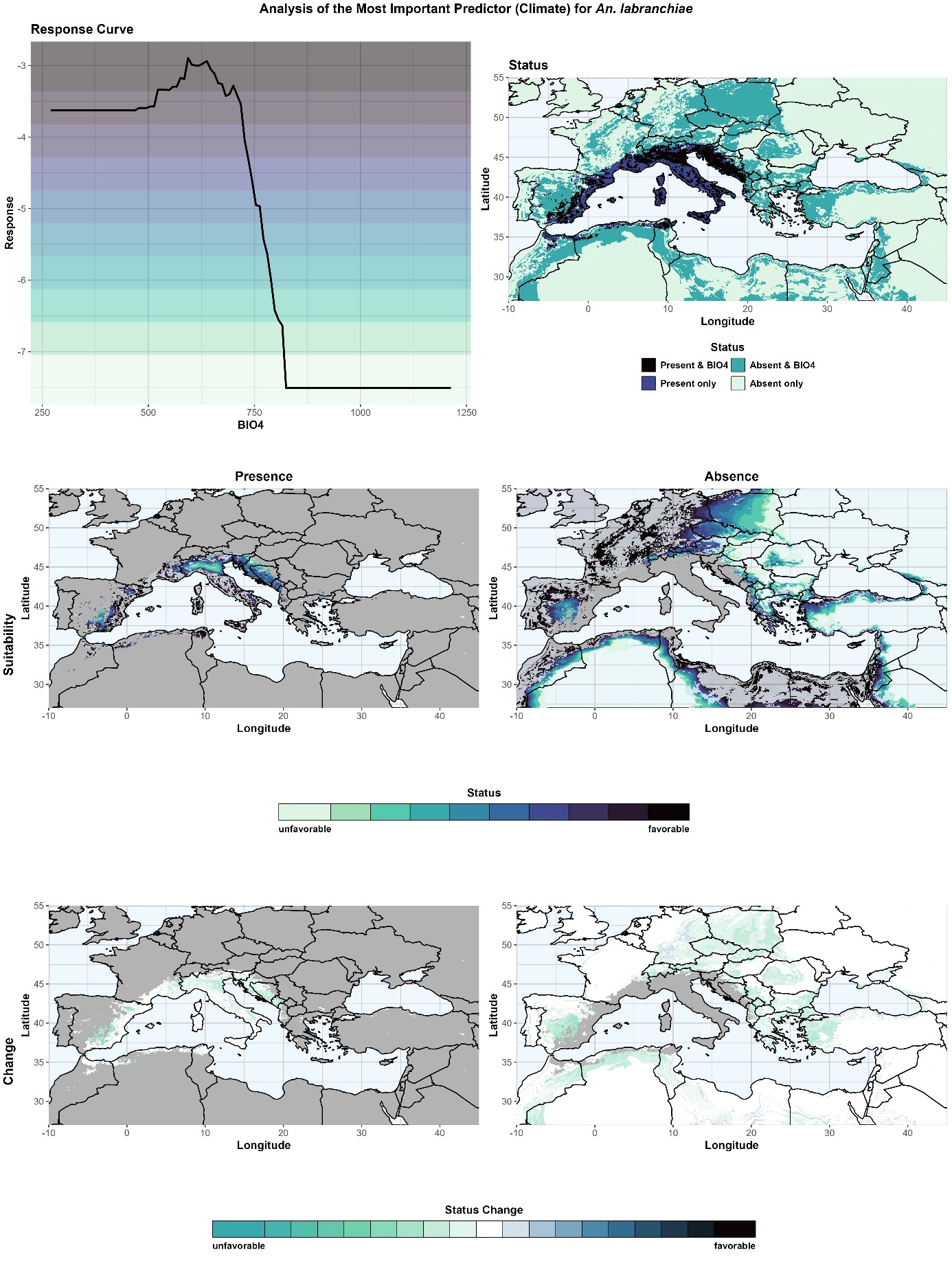


*Figure S4.2.1: Suitability under consideration of the most important climatic predictor for* ***An. labranchiae****. The response curves of the MIP and the status is presented at the top. Below, suitability maps are given for the presence (left) and absence areas (right), and changes in suitability at the bottom.*


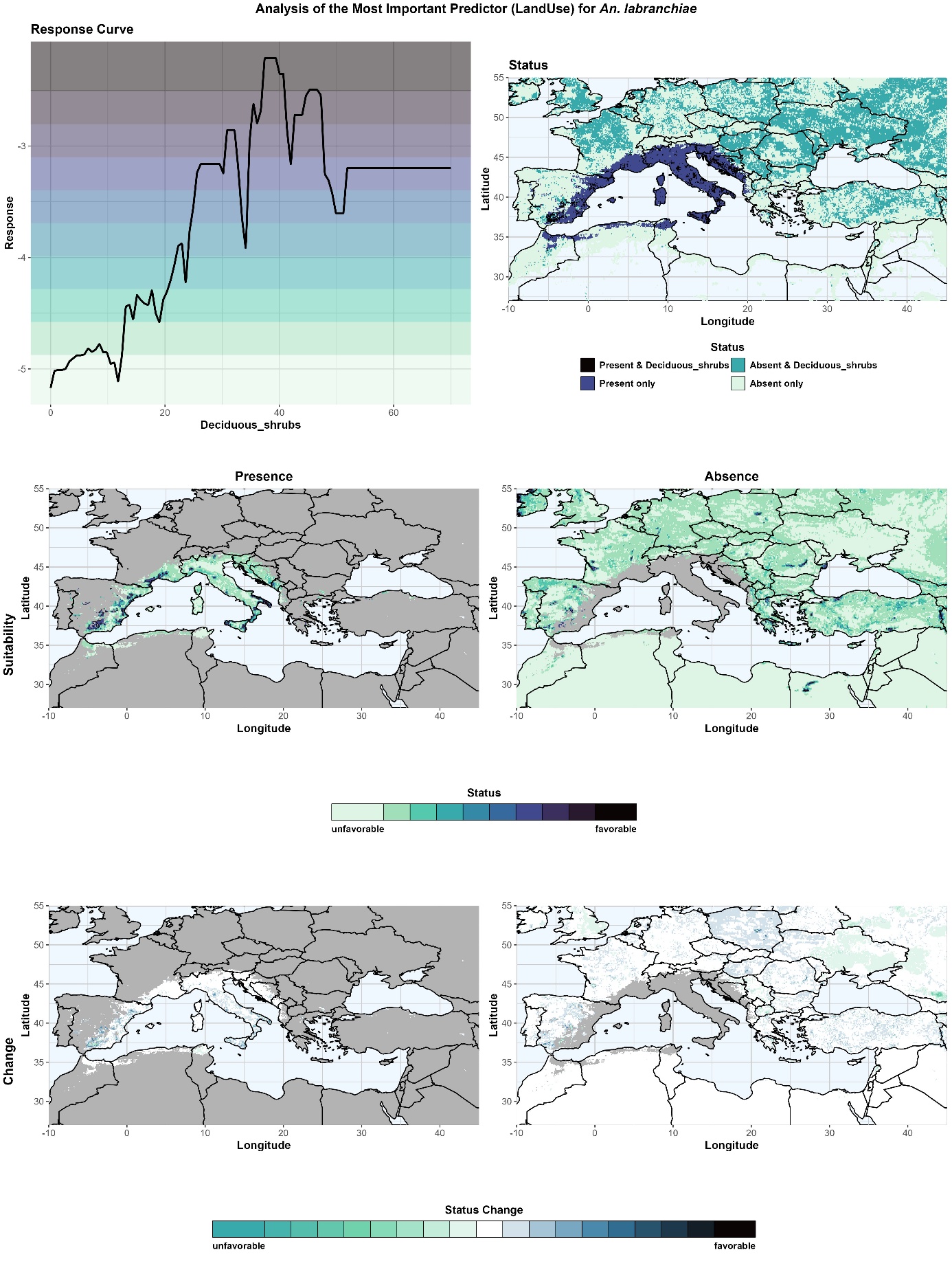


*Figure S4.2.2: Suitability under consideration of the most important land-use predictor for* ***An. labranchiae****. The response curves of the MIP and the status is presented at the top. Below, suitability maps are given for the presence (left) and absence areas (right), and changes in suitability at the bottom.*

S4.3 *An. messeae*


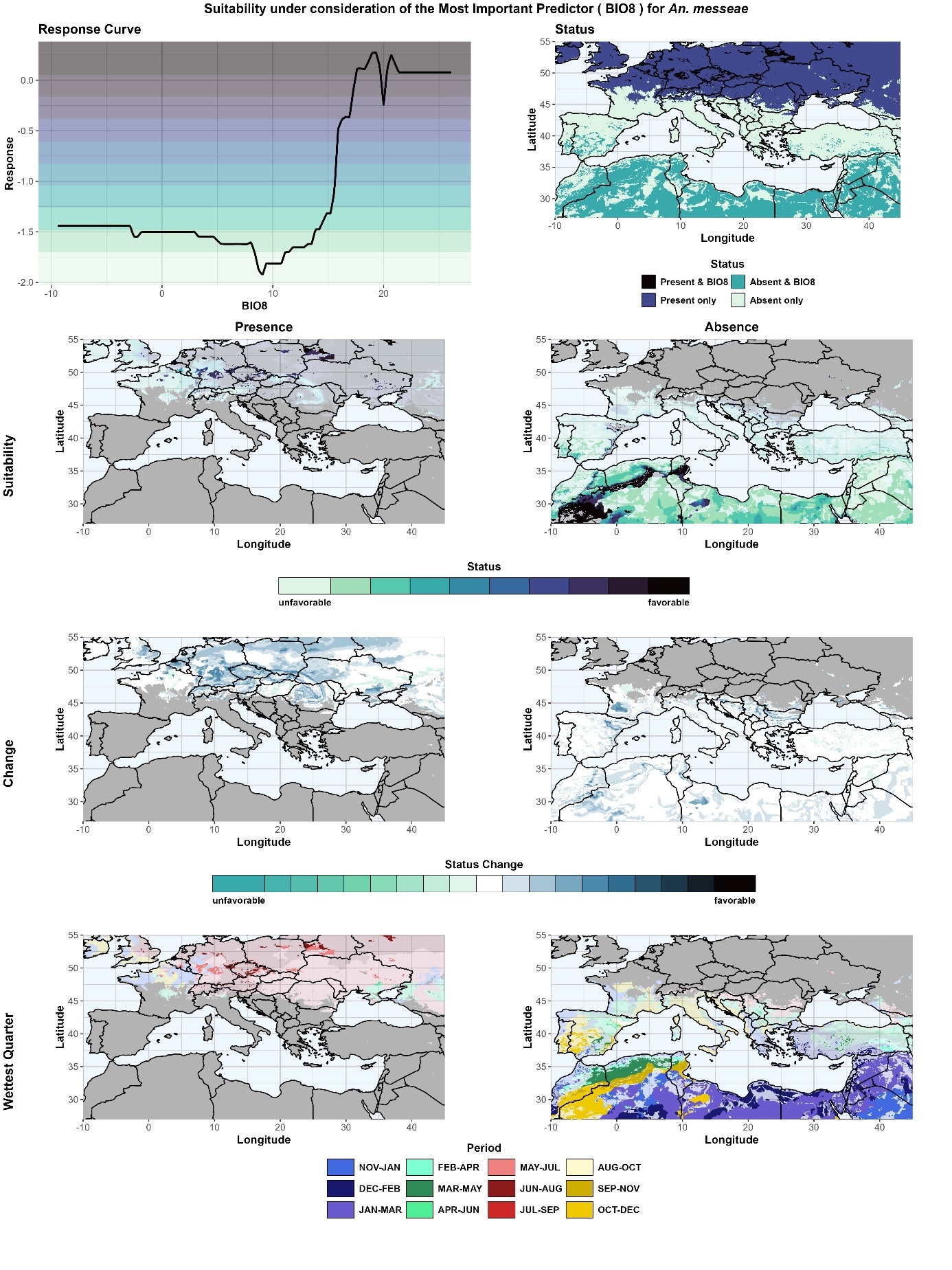


*Figure S4.3.1: Suitability under consideration of the most important climatic predictor for* ***An. messeae****. The response curves of the MIP and the status is presented at the top. Below, suitability maps are given for the presence (left) and absence areas (right), changes in suitability (3^rd^ row) and the reference quarter representing the warmest quarter obtained from ERA5-Land (bottom).*


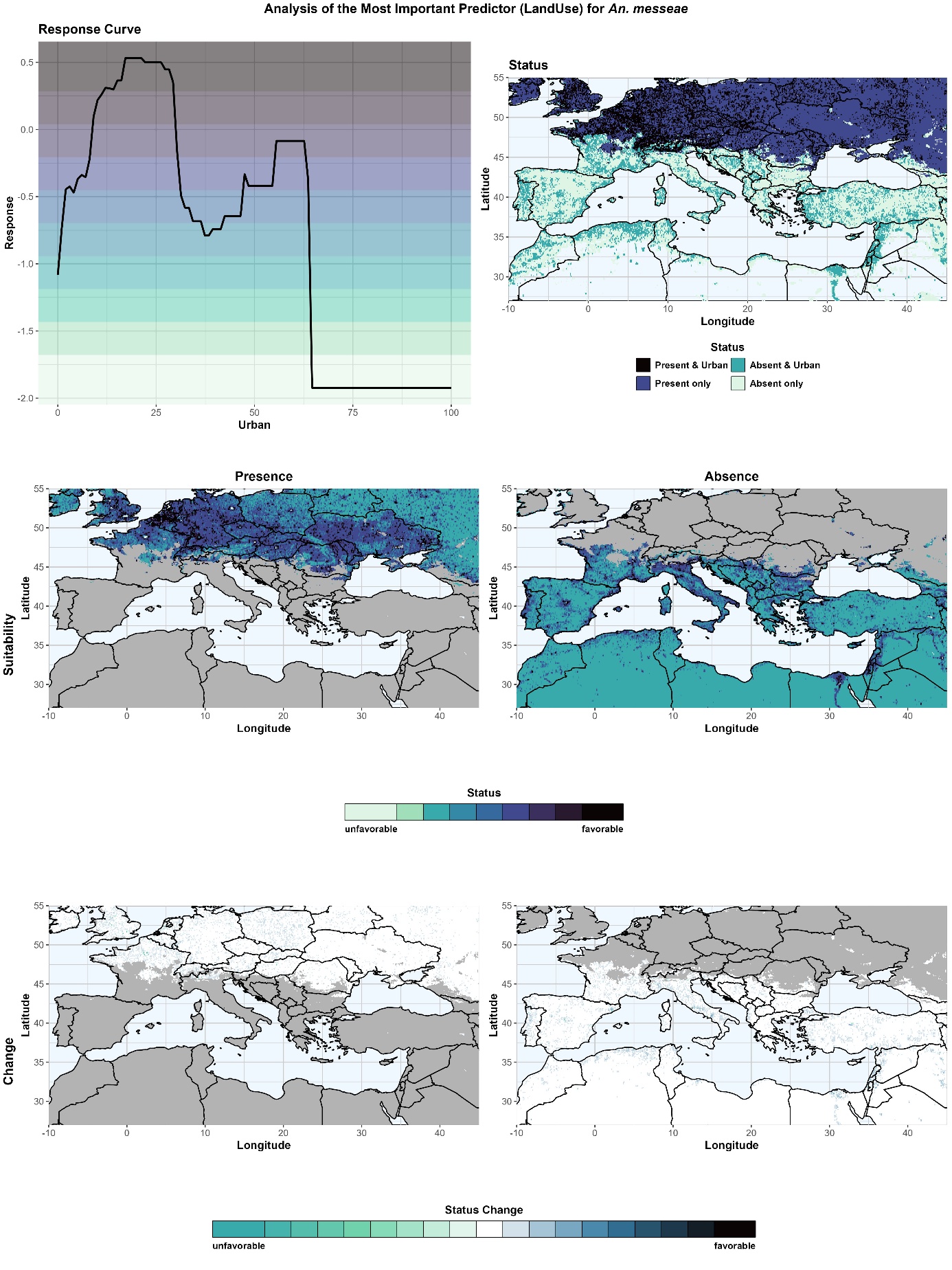


*Figure S4.3.2: Suitability under consideration of the most important land-use predictor for* ***An. messeae****. The response curves of the MIP and the status is presented at the top. Below, suitability maps are given for the presence (left) and absence areas (right), and changes in suitability at the bottom.*

S4.4 *An. sacharovi*


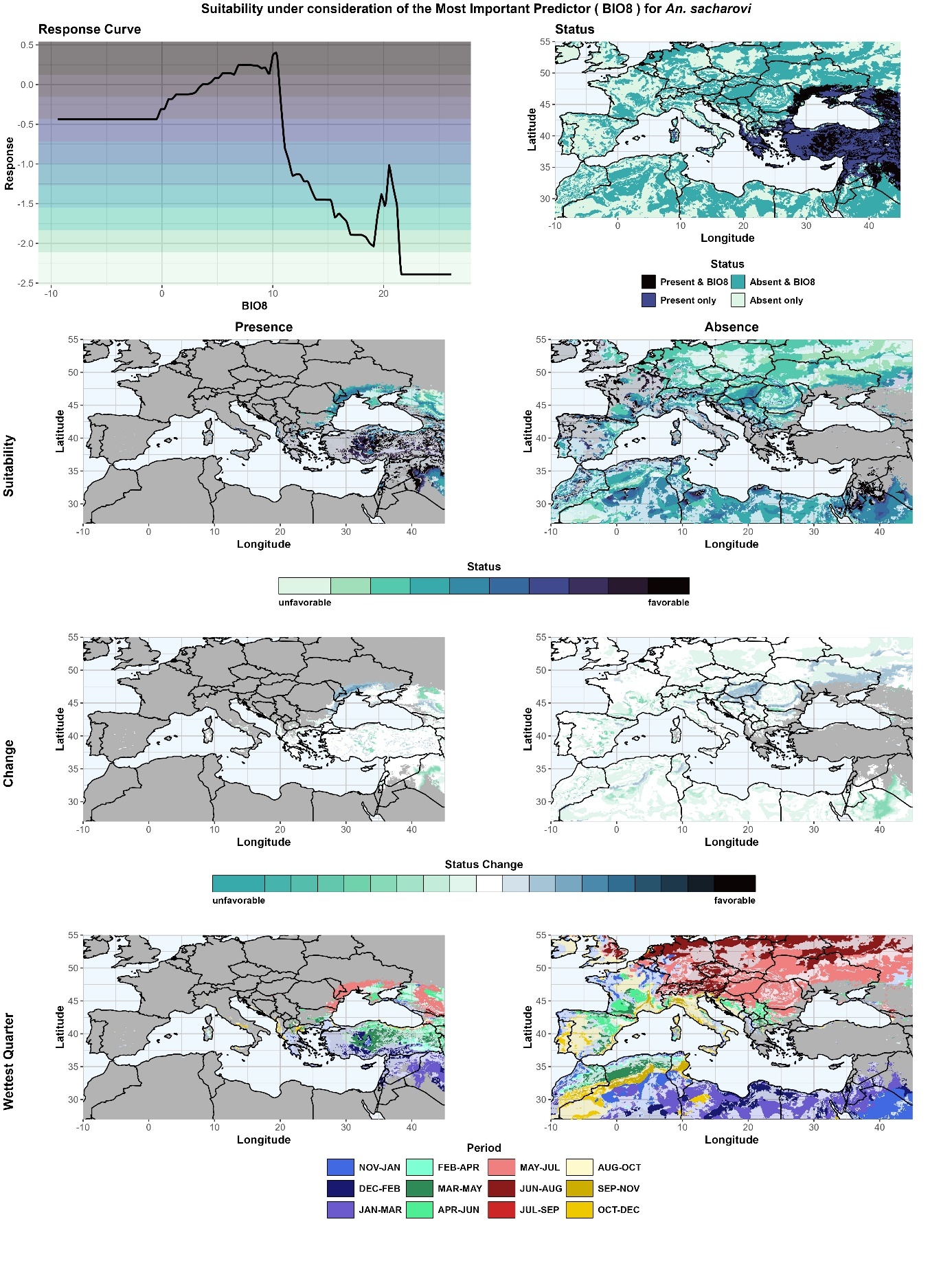


*Figure S4.4.1: Suitability under consideration of the most important climatic predictor for* ***An. sacharovi****. The response curves of the MIP and the status is presented at the top. Below, suitability maps are given for the presence (left) and absence areas (right), changes in suitability (3^rd^ row) and the reference quarter representing the warmest quarter obtained from ERA5-Land (bottom).*


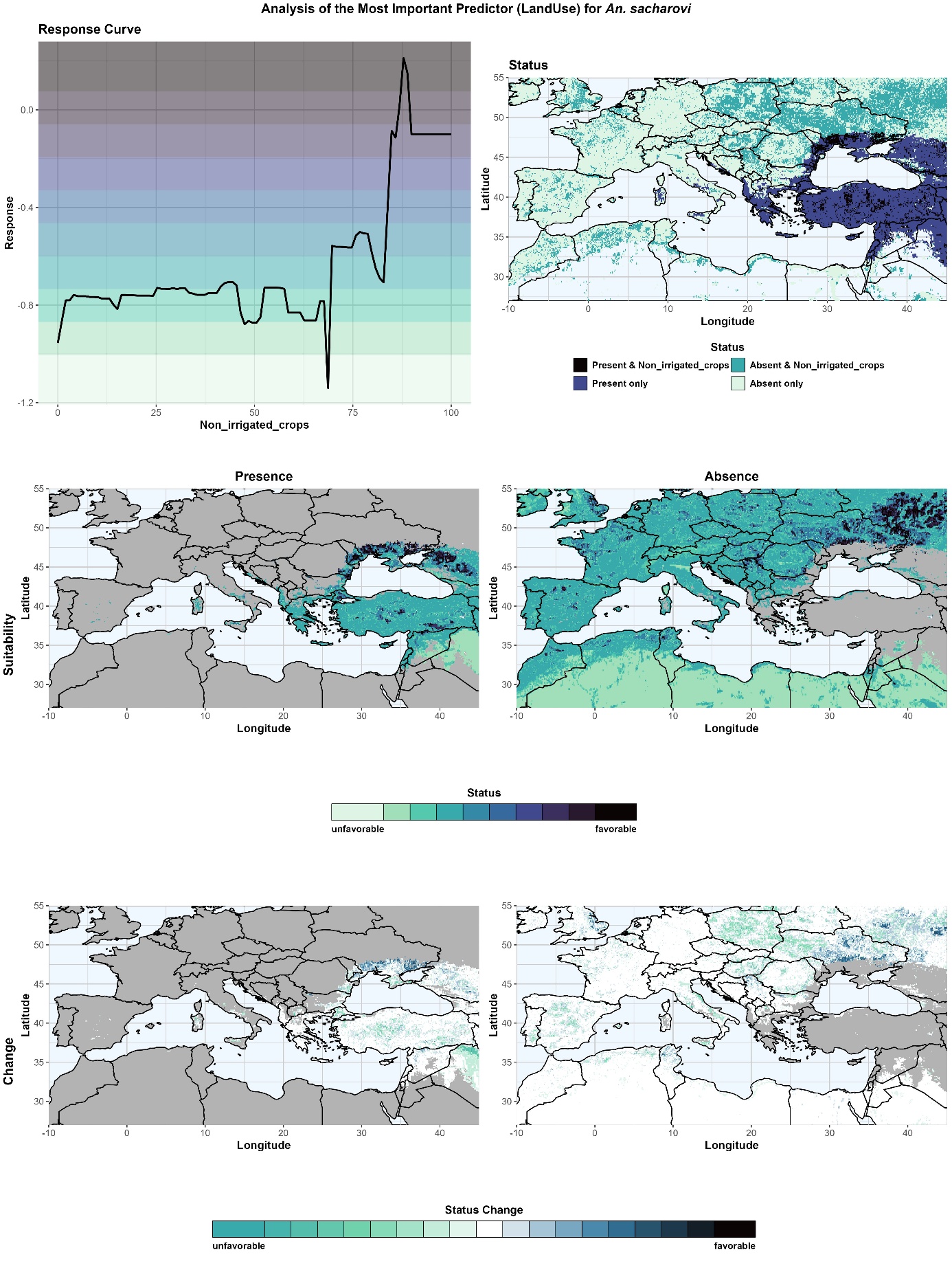


*Figure S4.4.2: Suitability under consideration of the most important land-use predictor for* ***An. sacharovi****. The response curves of the MIP and the status is presented at the top. Below, suitability maps are given for the presence (left) and absence areas (right), and changes in suitability at the bottom.*

S4.5 *An. sergentii*


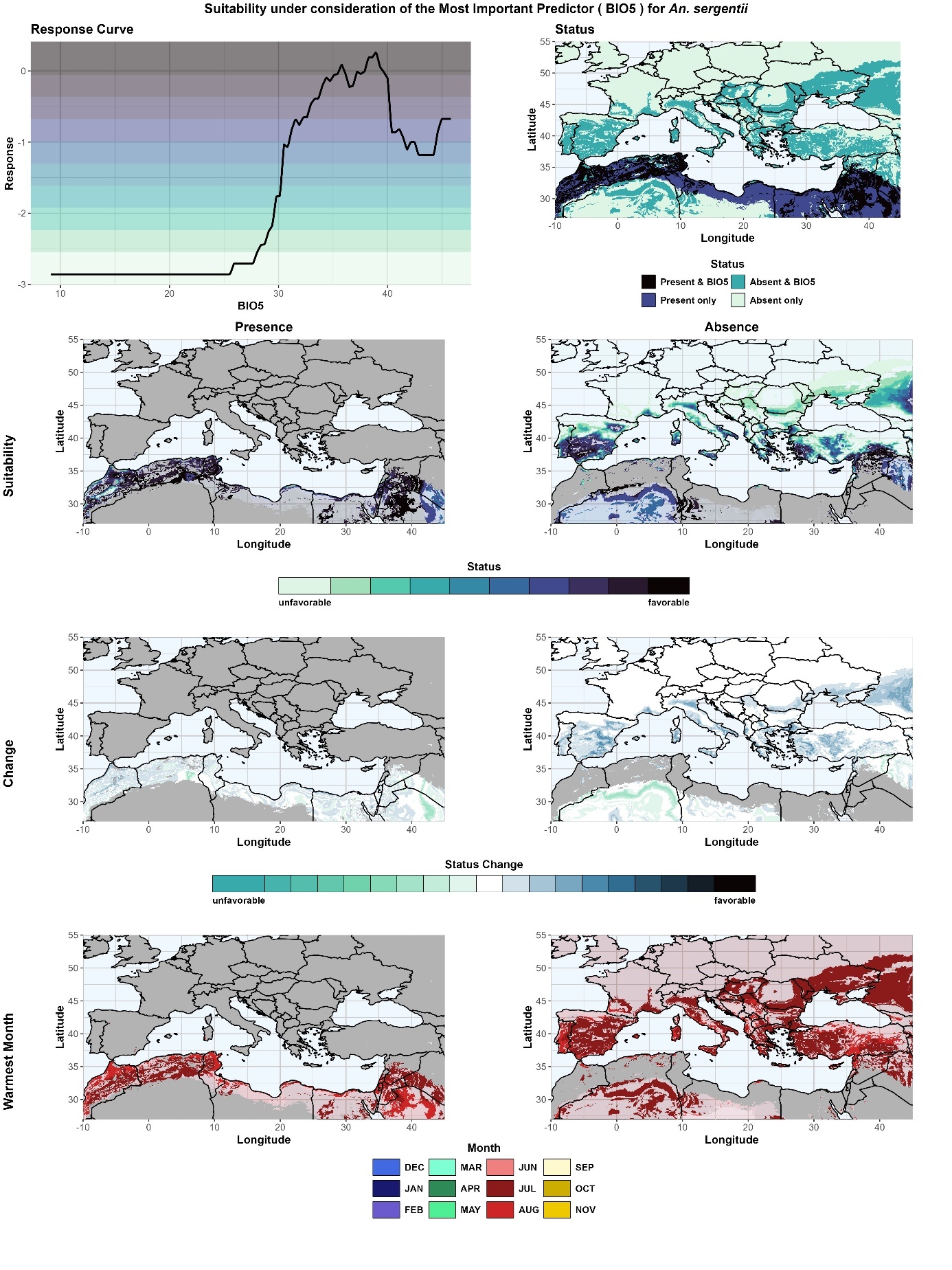


*Figure S4.5.1: Suitability under consideration of the most important climatic predictor for* ***An. sergentii****. The response curves of the MIP and the status is presented at the top. Below, suitability maps are given for the presence (left) and absence areas (right), changes in suitability (3^rd^ row) and the reference month representing the warmest month obtained from ERA5-Land (bottom).*


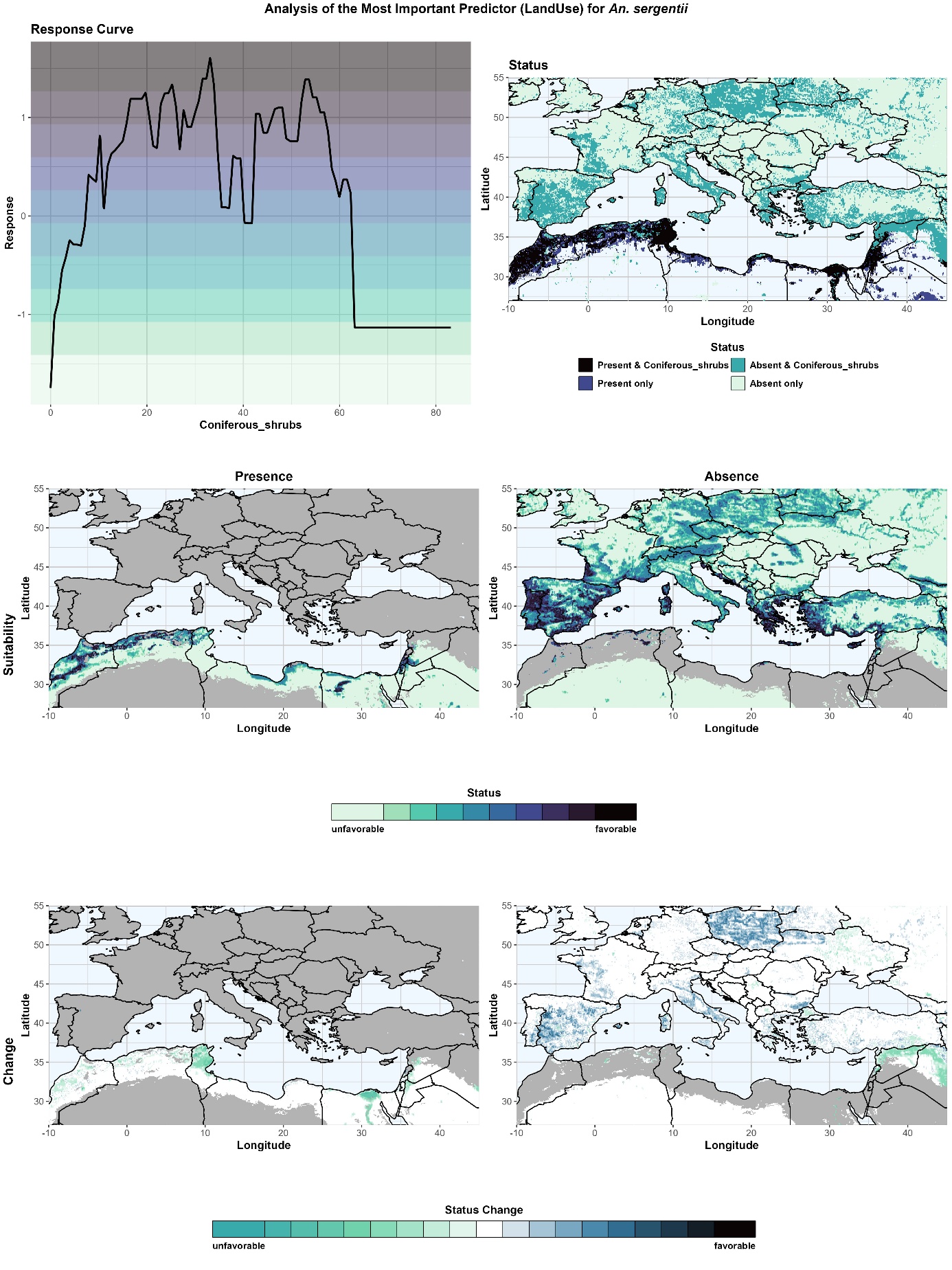


*Figure S4.5.2: Suitability under consideration of the most important land-use predictor for* ***An. sergentii****. The response curves of the MIP and the status is presented at the top. Below, suitability maps are given for the presence (left) and absence areas (right), and changes in suitability at the bottom.*

S4.6 *An. superpictus*


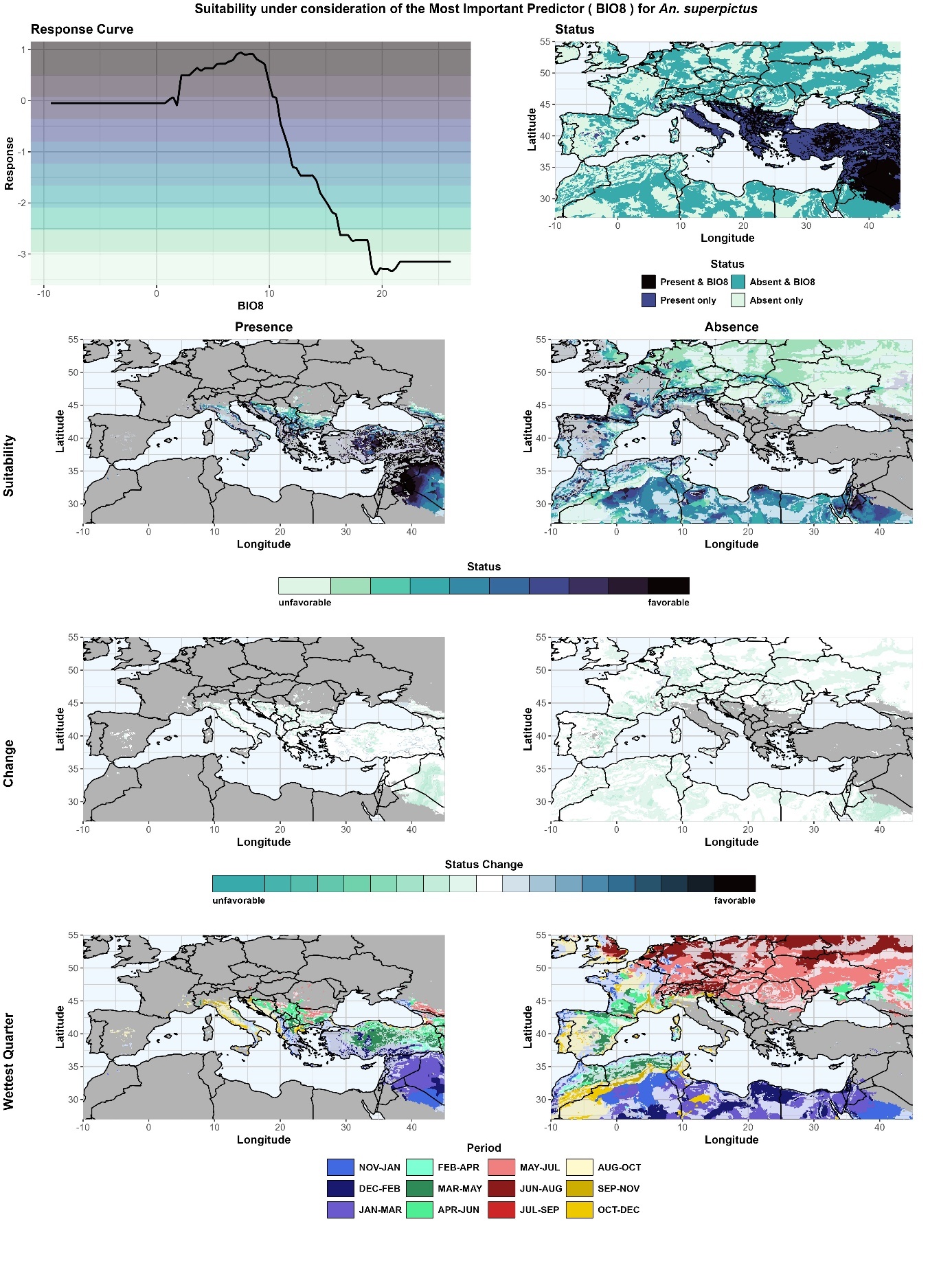


*Figure S4.6.1: Suitability under consideration of the most important climatic predictor for* ***An. superpictus****. The response curves of the MIP and the status is presented at the top. Below, suitability maps are given for the presence (left) and absence areas (right), changes in suitability (3^rd^ row) and the reference quarter representing the warmest quarter obtained from ERA5-Land (bottom).*


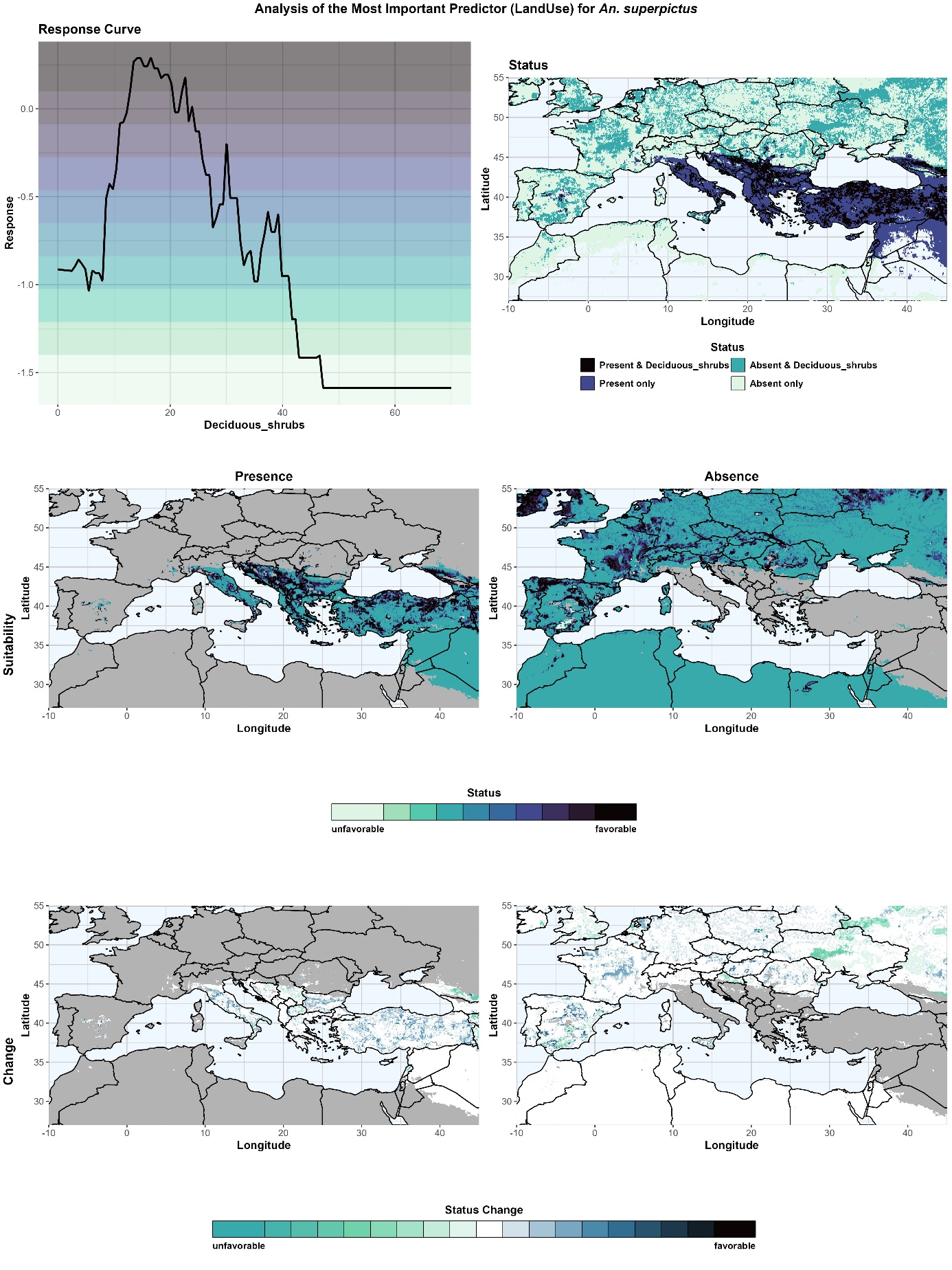


*Figure S4.6.2: Suitability under consideration of the most important land-use predictor for* ***An. superpictus****. The response curves of the MIP and the status is presented at the top. Below, suitability maps are given for the presence (left) and absence areas (right), and changes in suitability at the bottom.*
